# Spatial heterogeneity in intimate partner violence across the 640 districts of India: a secondary analysis of a cross-sectional, population-based survey by use of model-based small-area estimation

**DOI:** 10.1016/S2214-109X(23)00377-7

**Published:** 2023-09-19

**Authors:** Swati Srivastava, Kaushalendra Kumar, Lotus McDougal, Ajit Kumar Kannaujiya, Ankit Sikarwar, Anita Raj, Abhishek Singh

**Affiliations:** a Department of Public Health and Mortality Studies, Mumbai, India; b Center of Demography of Gender, Mumbai, India; c International Institute for Population Sciences, Mumbai, India; d Center on Gender Equity and Health, University of California San Diego, San Diego, CA, USA; e French Institute for Demographic Studies, Paris, France

## Abstract

**Background:**

Although intimate partner violence (IPV) against women is a substantial challenge in India, response is limited by little evidence on substate prevalence. District-level IPV estimates are essential in targeted response and prevention efforts, but cannot be directly calculated from the National Family Health Surveys (NFHS), which is the main source of nationally representative IPV estimates in India. We aimed to use small-area estimation techniques to derive reliable estimates of physical, emotional, and sexual IPV for the 640 districts of India.

**Methods:**

For this secondary analysis of a cross-sectional, population-based survey, we used model-based small-area estimation techniques linking data from the 2015–16 NFHS-4 and the 2011 Indian Population and Housing Census (2011 Indian Census) to derive district-level estimates of physical, emotional, and sexual IPV for the 640 districts of India in the previous 12 months. Only women who had ever been married aged 15–49 years, who were interviewed in NFHS-4, and who were included in the domestic violence module were eligible for inclusion in this analysis. Data collection occurred between Jan 20, 2015, and Dec 4, 2016. The 2011 Indian Census was conducted in all 640 districts from Feb 9 to Feb 28, 2011. It collected information on a range of data including sociodemographic data and housing characteristics. The primary outcomes of this analysis were the district-level mean proportions of women who experienced physical IPV, emotional IPV, and sexual IPV in the previous 12 months. This outcome was estimated for all women aged 15–49 years who had ever been married in the 640 districts of India that were included in the 2011 Indian Census.

**Findings:**

699 686 women aged 15–49 years were interviewed in NFHS-4. One woman per household in a randomly selected 15% of households was chosen for participation in the domestic violence module, resulting in 83 397 (11·9%) of 699 686 women included. Of these 83 397 women, 14 377 (17·2%) were excluded as they had never been married and 3007 (3·6%) were excluded due to privacy limitations. The mean prevalence of physical IPV in the previous 12 months was 22·5% (95% CI 21·9–23·2), of emotional IPV in the previous 12 months was 11·4% (11·0–11·9), and of sexual IPV in the previous 12 months was 5·2% (4·9–5·5). Model-based estimates revealed intrastate and interstate IPV variations. In Bihar, which had the highest state-level physical IPV prevalence (35·1%, 33·3–37·0), district-level estimates varied from 23·5% (23·0–23·9) in Siwan to 42·7% (42·3–43·1) in Purbi Champaran. In Tamil Nadu, which had the highest state-level emotional IPV prevalence (19·0%, 17·4–20·8), district estimates ranged between 13·7% (13·2–14·1) in Kanniyakumari and 30·2% (29·5–30·8) in Sivaganga. Bihar also had the highest state-level sexual IPV prevalence (11·1%, 9·9–12·4), with estimates ranging between 6·3% (6·1–6·6) in Siwan and 18·1% (17·6–18·6) in Saharsa. Across districts, there was substantial spatial clustering of IPV prevalence.

**Interpretation:**

This reliable district-level estimation of IPV prevalence in the 640 districts of India has important policy implications. The ability to track substate levels of IPV over time enables the identification of progress in reducing IPV; recognises the heterogeneity of culture and context in India; and informs the targeting of resources, interventions, and prevention programmes to districts with the greatest need.

**Funding:**

Bill & Melinda Gates Foundation.

## Introduction

Intimate partner violence (IPV) is a serious public health concern that curtails the safety, security, and wellbeing of women worldwide. Although IPV has substantial and harmful effects on men and people of other genders, this Article focuses specifically on women. IPV, which can include physical, sexual, and emotional abuse from partners, has many short-term and long-term effects on the health of women, including compromised sexual and reproductive health, sexually transmitted infections, and pregnancy complications.^1^, ^2^ Furthermore, IPV can cause social isolation, unemployment, income loss, and poor self-care.^3^

**Research in context**

**Evidence before this study**

We searched PubMed, Scopus, and Google Scholar using the search terms (“spatial heterogeneity” OR “spatial distribution”) AND (“intimate partner violence” OR “domestic violence” OR “physical violence” OR “emotional violence” OR “sexual violence”) AND “districts” AND “small-area estimation” AND “India” for studies published from database inception to March 25, 2023, without language restrictions. No previous studies that are indexed in these databases have examined spatial heterogeneity in intimate partner violence (IPV) across the districts of India.

**Added value of this study**

This analysis is the first to use model-based, small-area estimation techniques to generate district-level estimates of physical, emotional, and sexual IPV for the 640 districts of India. Diagnostic measures indicated that these estimates are unbiased, consistent, and reliable. District-level IPV estimates revealed considerable intrastate variations in IPV, which are often hidden by the state-level mean. For example, in Bihar, which has the highest prevalence of physical IPV (35·1%), district-level estimates in the previous 12 months varied from 23·5% in Siwan to 42·7% in Purbi Champaran. The prevalence of physical IPV was lowest in Sikkim (1·8%), where district-level estimates in the previous 12 months varied between 1·1% in East Sikkim and 2·0% in West Sikkim.

**Implications of all the available evidence**

Estimates of IPV in India have previously only reliably been estimable at the state level. In a nation as large and heterogeneous as India, this inhibits the ability to develop and refine responsive policies and services to target available resources as effectively as possible. Our analysis highlights substantial variation in the prevalence of physical, emotional, and sexual IPV across the 640 districts in India. Therefore, our findings inform the work of policy makers and programme managers to directly incorporate the consideration of local culture, context, and narratives of gender to address the burden of IPV in India. Our analysis also allows for the exploration of the relationship between IPV and other important health, social, and developmental factors at local levels, including gender inequality, consumption of alcohol or other substances, and natural disasters. Researchers and policy makers from other low-income and middle-income countries, where data are not readily available at the local level, might also benefit from our example of using commonly available household survey data (eg, the National Family Health Survey, which is the Indian implementation of the Demographic and Health Surveys) and national census data to apply small-area estimation to estimate key metrics (eg, IPV, the empowerment of women, and the digital and financial inclusion of women) that have, to date, been limited to more aggregate assessments.

Globally, 13% of women experienced IPV in the 12 months before they were surveyed; in south Asia, this estimate is 19%.^4^ In India, estimates from the 2019–21 National Family Health Survey (NFHS-5) revealed that approximately 32% of women who had ever been married experienced physical, emotional, or sexual IPV in the previous 12 months.^5^ These estimates, however, vary substantially across locations within India. There is substantial interstate disparity in the burden of IPV, ranging from 48% in Karnataka to 2% in Lakshadweep.^5^ IPV is also more prevalent in rural areas (34%) than in urban areas (27%).

The elimination of violence against women and girls is an important target (5.2) of Sustainable Development Goal (SDG) 5, which prioritises gender equality and empowerment and tracks progress on this Goal via the prevalence of physical and sexual IPV in the previous 12 months.^6^ To understand the extent of the problem, and to monitor global progress, estimates of these indicators at more local geographical levels, such as at the district level in India, are essential to have so that areas with a high burden of IPV can be effectively targeted. The focus of several development programmes in India has changed from the state level to the district level, including the creation of the Aspirational Districts Programme (ADP).^7^

Launched in January, 2018, the ADP aims to quickly and effectively improve the 112 most underdeveloped districts across the country. The broad aims of the programme are convergence of central and state schemes, collaboration between central and state-level nodal officers and district collectors, and competition among districts through Delta ranking once per month.

With states as the main drivers, the ADP focuses on the strength of each district, identifying areas for immediate improvement and measuring progress. The Delta ranking is based on the incremental progress made across 49 key performance indicators under five broad themes (ie, health and nutrition, education, agriculture and water resources, financial inclusion, and skill development and infrastructure). The Delta ranking and performance of all districts is available on the Champions of Change Dashboard.

Several efforts in the past 5 years have been made to monitor the progress of the SDGs and other indicators across the districts of India.^8^, ^9^, ^10^

In India, the NFHS is the main source of data providing estimates of IPV at the national and state levels. However, this survey is designed to provide representative estimates of IPV only at the state level. As a result, direct calculations of reliable district-level estimates of IPV from NFHS data are impossible, which is a substantial limitation in a nation where more than half of states and union territories have populations of 10 million people or more.^11^ Furthermore, most states include substantial ethnic, linguistic, and religious diversity, which contribute to the norms that influence the acceptability and perpetration of gender-based violence.^12^

To address this deficit in localised understanding of the prevalence of gender-based violence across India, we aimed to use small-area estimation techniques to derive reliable estimates of physical, emotional, and sexual IPV for the 640 districts of India. We also aimed to examine the spatial heterogeneity of IPV estimates to assess if our state estimates are hiding potential IPV hotspots (ie, clusters of districts that have a high prevalence of IPV in India). Identifying hotspots is essential when trying to address pervasive and persistent behaviours, such as IPV, that are sustained by patriarchal social norms and the long-term devaluation of women, particularly in a setting as large and diverse as India.

## Methods

### Study design and respondents

This secondary analysis used data from NFHS-4 (a cross-sectional, population-based survey for which data were collected during 2015–16) and the 2011 Indian Population and Housing Census (2011 Indian Census). NFHS-4 surveyed 601 509 households with women who had ever been married aged 15–49 years. NFHS-4 obtained data via in-person interviews conducted at visits with each individual. Data collection occurred between Jan 20, 2015, and Dec 4, 2016. The response rate of individual women was 97%.^13^ Sex data were reported by the head of the household; the options provided were male or female.

The most recent Indian Population and Housing Census, the 2011 Indian Census, was conducted in all 640 districts (spread across 28 states and eight union territories) from Feb 9 to Feb 28, 2011. This census collected information on a range of data including sociodemographic data, housing characteristics, fertility, and migration status.^11^ Data collectors visited households to collect information. Gender data were self-reported; the options provided were male, female, or other.

The questions in NFHS-4 to assess IPV are provided in the appendix (pp 1–2). The 2011 Indian Census did not ask any questions related to IPV.

A domestic violence module, consisting of a set of questions about each woman's experience of domestic violence, perpetrator of violence, reporting of domestic violence, and help-seeking behaviour, was conducted with one woman per household in a randomly selected 15% of households in NFHS-4. The 2011 Indian Census did not ask any question related to domestic violence.

Our analysis is based on a secondary dataset with no identifiable information on survey respondents. The dataset is available for research use from the Demographic and Health Surveys website. Ethics exemption for this analysis was provided by the University of California San Diego (180070XX). Ethics approval for the original data collection was provided by the International Institute for Population Sciences Institutional Review Board and the ICF Institutional Review Board. All respondents provided oral informed consent before being interviewed. This consent was taken for participation in the survey only; NFHS data is always made publicly available for secondary data analysis. The study protocol is available online.

### Auxiliary variables

In small-area estimation analysis, two types of variables are required: outcome variables and auxiliary variables. Outcome variables measure the outcome of interest and are generally derived from surveys. Auxiliary variables capture specific sociodemographic factors and are required for the entire population; typically, these auxiliary variables are available from a census or from administrative records.^14^ We therefore included district-level information from the 2011 Indian Census as auxiliary variables: scheduled castes or scheduled tribes (all language regarding caste used in this Article is official categories used by the Government of India), religion, amount of female workforce participation and sex gap relative to male workforce participation, female literacy and sex gap relative to male literacy, female head of household, household size, male migration status in the previous 12 months, female age at marriage, birth of a male child in the previous 12 months, sex ratio at birth, urban residence, socioeconomic status, and state of residence. The choice of auxiliary variables for our models was guided by the social, economic, and demographic determinants of IPV that have been identified in previous literature (appendix pp 2–3).^15^, ^16^

State borders are ultimately administrative and individuals living in districts that are close to either side of the border might be more similar to each other (with regard to norms relating to gender-based violence) than to people living in the same state but further from the border, as districts across the state boundaries often share common characteristics, cultural norms and practices, and increased cross-state mobility.^17^ To account for the potential influence of neighbouring districts on a district, we included a border proximity factor (BPF) in the small-area estimation models. BPF was calculated as the mean Euclidean distance of districts from state borders (appendix pp 3, 5–16, 29).

### Outcomes

The primary outcomes of this analysis were the district-level mean proportions of women who experienced physical IPV, emotional IPV, and sexual IPV in the previous 12 months. This outcome was estimated for all women aged 15–49 years who had ever been married in the 640 districts of India that were included in the 2011 Indian Census (appendix p 1).

### Statistical analysis

Weighted prevalences were calculated to estimate sample demographic characteristics (NFHS-4 provides weights that can be used to derive estimates of demographic data). Survey weights were used to ensure that these prevalences were representative at the population level.

Small-area estimation techniques are classified into two broad types: unit-level and area-level random-effects models. Unit-level random-effects models are used when auxiliary data are available at the individual level, whereas area-level random-effects models are used when auxiliary variables are only available at an aggregate level (eg, district level).^14^, ^18^, ^19^ We used the area-level small-area estimation approach as auxiliary variables were only available at the district level.

We first derived district-level estimates of physical, emotional, and sexual IPV directly from the NFHS-4 dataset (henceforth referred to as direct survey-based estimates), accounting for NFHS-4 domestic violence weights. These direct survey-based estimates were then linked to auxiliary variables via generalised linear mixed models (GLMM) with logit link functions to derive the model-based district-level estimates for the 640 districts of India.^14^, ^18^, ^20^ The GLMM accounted for area-specific random effects, which provided strength to the model-based district-level estimates of physical, emotional, and sexual IPV.^21^

Two types of diagnostic measures (ie, model diagnostic and diagnostic for the small-area estimates) were used to assess the validity of the fitted GLMM models and the reliability of the model-based district-level estimates of IPV.^14^, ^18^ Accordingly, we used model diagnostic and diagnostic for the small-area estimates to assess the validity and reliability of our estimates (appendix pp 3–4). A p value of less than 0·05 was considered significant in the model diagnostic.

Small-area estimation is even more important than usual if there is spatial heterogeneity in the outcomes of interest. Spatial-heterogeneity analysis can identify and target high-IPV clusters of districts, even in low-prevalence states. We used a univariate local indicator of spatial association (LISA) approach to obtain geographical clustering of IPV in India (appendix p 4).

Small-area estimation was done with Stata version 16. LISA was estimated with GeoDa version 1.12.1.161.

### Role of the funding source

The funder of the study had no role in study design, data collection, data analysis, data interpretation, or writing of the report.

## Results

699 686 women aged 15–49 years were interviewed in NFHS-4 between Jan 20, 2015, and Dec 4, 2016. One woman per household in a randomly selected 15% of households was chosen for participation in the domestic violence module, resulting in 83 397 (11·9%) of 699 686 women included. Of these 83 397 women, 14 377 (17·2%) were excluded as they had never been married and 3007 (3·6%) were excluded due to privacy limitations. This analysis includes all 66 013 women who had ever been married aged 15–49 years and who were eligible for participation in the domestic violence module. All women who were not selected for the domestic violence module were excluded from this analysis.

46 544 (weighted percentage 65·3%) of 66 013 women lived in a rural area, 23 794 (28·7%) were scheduled castes or scheduled tribes, 49 546 (81·1%) were Hindu, 12 838 (17·0%) were in the lowest wealth quintile,^13^ 13 970 (19·5%) were aged 25–29 years, 62 716 (94·5%) were currently married, and 22 028 (32·5%) had no formal education (table). There were no respondents with missing data. The mean prevalence of physical IPV in the previous 12 months was 22·5% (95% CI 21·9–23·2), of emotional IPV in the previous 12 months was 11·4% (11·0–11·9), and of sexual IPV in the previous 12 months was 5·2% (4·9–5·5).

**Table:** Sample characteristics and estimated prevalence of IPV by demographic characteristics

		**Weighted number of respondents**	**Weighted proportion of respondents**	**Estimated prevalence of physical IPV in the previous 12 months**	**Estimated prevalence of emotional IPV in the previous 12 months**	**Estimated prevalence of sexual IPV in the previous 12 months**
India		66 013	100·0%	22·5% (21·9–23·2)	11·4% (11·0–11·9)	5·2% (4·9–5·5)
Age of respondents, years						
	15–19	1642	3·5%	16·6% (14·1–19·2)	11·4% (8·8–14·0)	4·8% (3·2–6·3)
	20–24	8847	14·9%	22·3% (20·6–23·9)	10·8% (9·8–11·8)	5·0% (4·4–5·6)
	25–29	13 970	19·5%	23·2% (22·1–24·4)	11·1% (10·3–11·9)	5·7% (5·1–6·2)
	30–34	13 598	17·7%	23·5% (22·4–24·7)	11·7% (10·7–12·6)	5·6% (5·0–6·2)
	35–39	11 402	16·6%	23·2% (21·9–24·5)	12·0% (10·9–13·0)	5·3% (4·5–6·0)
	40–44	8677	14·3%	22·8% (21·3–24·4)	11·6% (10·5–12·7)	4·8% (4·1–5·5)
	45–49	7877	13·6%	20·9% (19·4–22·4)	11·5% (10·2–12·8)	4·5% (3·6–5·4)
Education						
	No education	22 028	32·5%	30·2% (29·2–31·2)	15·0% (14·2–15·8)	7·0% (6·4–7·6)
	Primary school	9669	14·3%	26·4% (24·6–28·3)	12·6% (11·5–13·7)	5·9% (5·2–6·6)
	Secondary school	28 187	42·8%	18·4% (17·6–19·2)	9·8% (9·1–10·4)	4·2% (3·8–4·6)
	Higher education	6129	10·4%	10·1% (8·7–11·5)	5·5% (4·6–6·5)	2·3% (1·7–3·0)
Marital status						
	Married	62 716	94·5%	22·5% (21·8–23·1)	11·2% (10·7–11·7)	5·1% (4·8–5·4)
	Widowed	2358	4·0%	18·3% (15·6–20·9)	9·3% (7·2–11·5)	4·9% (3·3–6·5)
	Divorced	341	0·5%	40·4% (31·3–49·5)	37·3% (28·3–46·2)	11·8% (6·6–17·1)
	No longer living together or separated	598	1·1%	37·5% (31·5–43·6)	29·6% (23·8–35·3)	11·6% (8·2–14·9)
Caste*						
	Scheduled castes	11 686	19·5%	28·4% (26·9–29·8)	14·6% (13·6–15·7)	6·7% (6·0–7·3)
	Scheduled tribes	12 108	9·2%	26·0% (24·3–27·8)	13·0% (11·3–14·7)	6·5% (5·5–7·6)
	Other backward class	25 574	44·2%	23·7% (22·9–24·6)	11·7% (11·0–12·3)	5·2% (4·7–5·6)
	Other†	16 645	27·2%	15·2% (13·9–16·6)	8·2% (7·4–9·0)	3·6% (3·1–4·2)
Religion						
	Hindu	49 546	81·1%	23·1% (22·4–23·9)	11·6% (11·1–12·1)	5·1% (4·8–5·5)
	Muslim	8614	13·7%	20·0% (18·5–21·6)	11·4% (10·2–12·6)	5·4% (4·4–6·4)
	Christian	4639	2·4%	22·2% (19·0–25·4)	11·6% (8·9–14·3)	5·2% (3·7–6·6)
	Sikh	1325	1·4%	16·7% (13·5–19·8)	6·1% (4·3–7·8)	4·1% (1·8–6·4)
	Other‡	1889	1·4%	18·2% (14·2–22·2)	7·3% (5·3–9·3)	4·9% (3·1–6·7)
Wealth quintile^13^						
	Lowest	12 838	17·0%	33·2% (31·9–34·4)	16·0% (15·1–17·0)	8·9% (8·2–9·7)
	Second	13 992	19·3%	28·0% (26·6–29·5)	13·7% (12·7–14·6)	6·1% (5·5–6·8)
	Middle	13 790	20·7%	23·4% (22·3–24·6)	12·1% (11·1–13·1)	5·2% (4·6–5·8)
	Fourth	13 142	21·2%	19·2% (18·0–20·4)	9·9% (9·0–10·8)	3·9% (3·4–4·5)
	Highest	12 251	21·7%	11·7% (10·7–12·8)	6·7% (5·7–7·7)	2·5% (1·9–3·1)
Residence						
	Urban	19 469	34·7%	18·5% (17·2–19·8)	10·1% (9·2–11·1)	4·0% (3·4–4·5)
	Rural	46 544	65·3%	24·7% (24·0–25·4)	12·1% (11·6–12·6)	5·8% (5·4–6·2)
Region of India						
	North	14 062	13·3%	15·9% (15·0–16·9)	7·4% (6·7–8·1)	3·5% (3·0–4·1)
	Central	14 941	21·2%	25·4% (24·4–26·5)	11·0% (10·2–11·7)	5·7% (5·2–6·2)
	East	11 614	22·4%	25·8% (24·2–27·5)	12·3% (11·3–13·2)	7·6% (6·8–8·5)
	Northeast	8766	3·4%	17·2% (15·4–19·0)	9·2% (8·0–10·4)	4·7% (3·8–5·6)
	West	6696	15·6%	14·2% (12·6–15·8)	8·7% (7·5–9·9)	2·2% (1·6–2·8)
	South	9934	24·0%	26·7% (25·1–28·3)	15·3% (13·9–16·7)	5·3% (4·5–6·0)

Data are % (95% CI) unless otherwise specified. IPV=intimate partner violence.

* All language regarding caste used in this Article is official categories used by the Government of India.

† Other includes anyone who does not identify as scheduled castes, scheduled tribes, or other backward class.

‡ Other includes Buddhist, Neo-Buddhist, Jain, Jewish, Parsi, Zoroastrian, and no religion.

**Figure 1:**
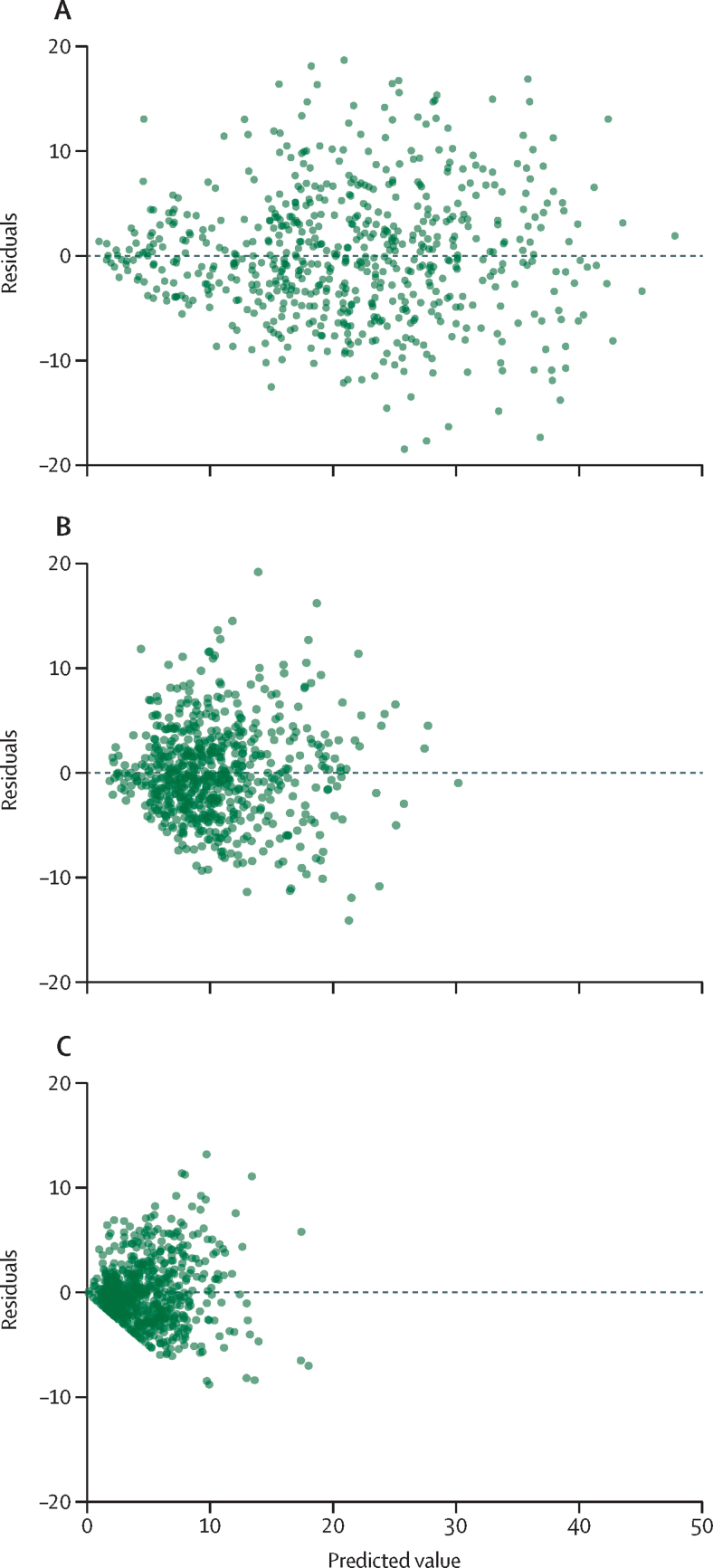
Model diagnostic plot showing scatterplots between predicted values and residuals for physical, emotional, and sexual violence experienced by women in the previous 12 months (India, 2015–16) (A) Physical violence. (B) Emotional violence. (C) Sexual violence.

**Figure 2:**
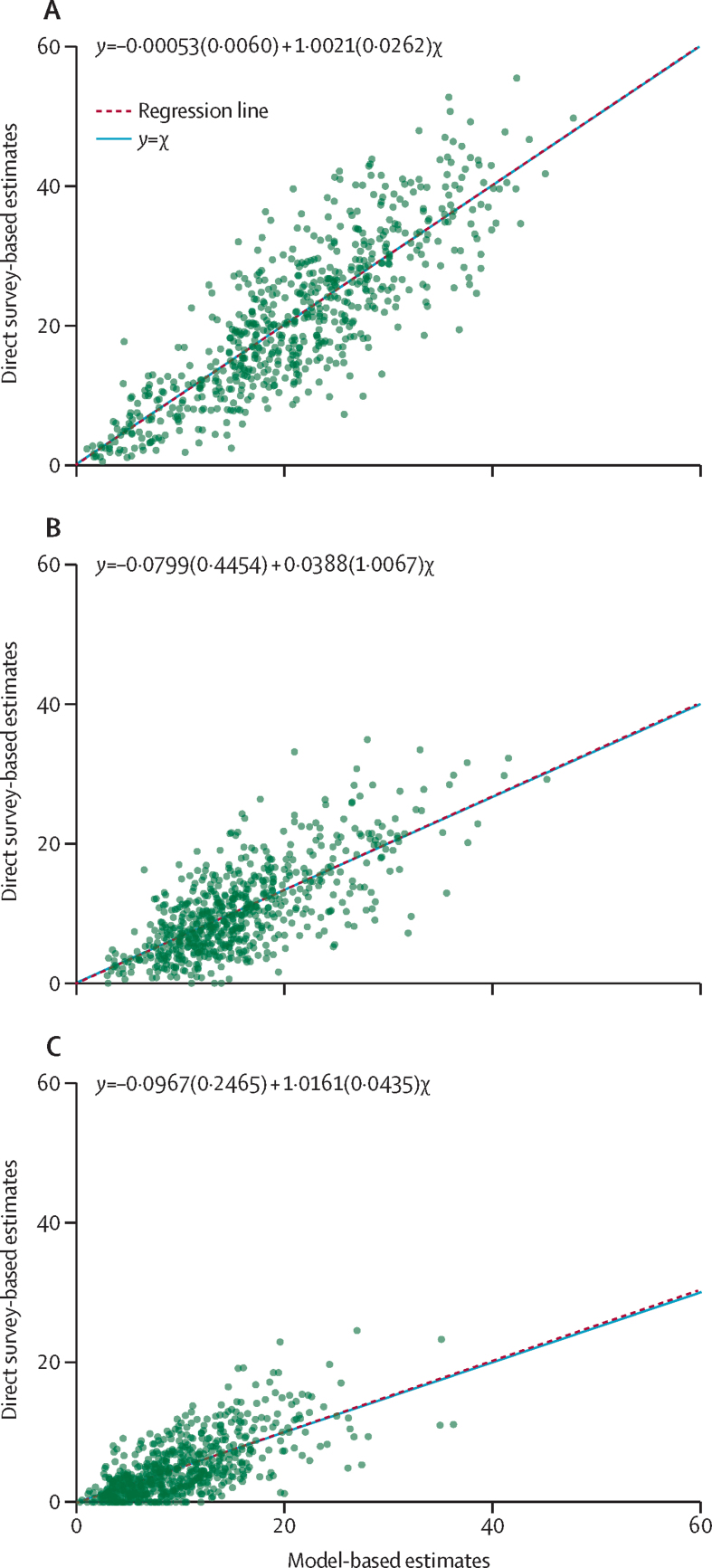
Scatterplots comparing the ordinary least-squares regression line and y=χ (India, 2015–16) (A) Proportion of women who experienced physical violence in the previous 12 months. (B) Proportion of women who experienced emotional violence in the previous 12 months. (C) Proportion of women who experienced sexual violence in the previous 12 months. Equations refer to the line of best fit.

The district-level residuals of physical and emotional IPV were randomly distributed (figure 1), which supports the normality assumption and suggests that the model-based estimates of IPV are robust (ie, close to their expected values). District-level residuals of sexual IPV deviated from the assumption of constant variance. When plotting direct survey-based district-level estimates of IPV against model-based district-level estimates (figure 2), we compared the closeness of the 45° line (*y=*χ) to the fitted regression line to examine the consistency between the two. The line of best fit was not significantly different from the line *y=*χ for the model-based estimates (at the p<0·05 level), indicating the consistency between the model-based and direct survey-based estimates.

**Figure 3:**
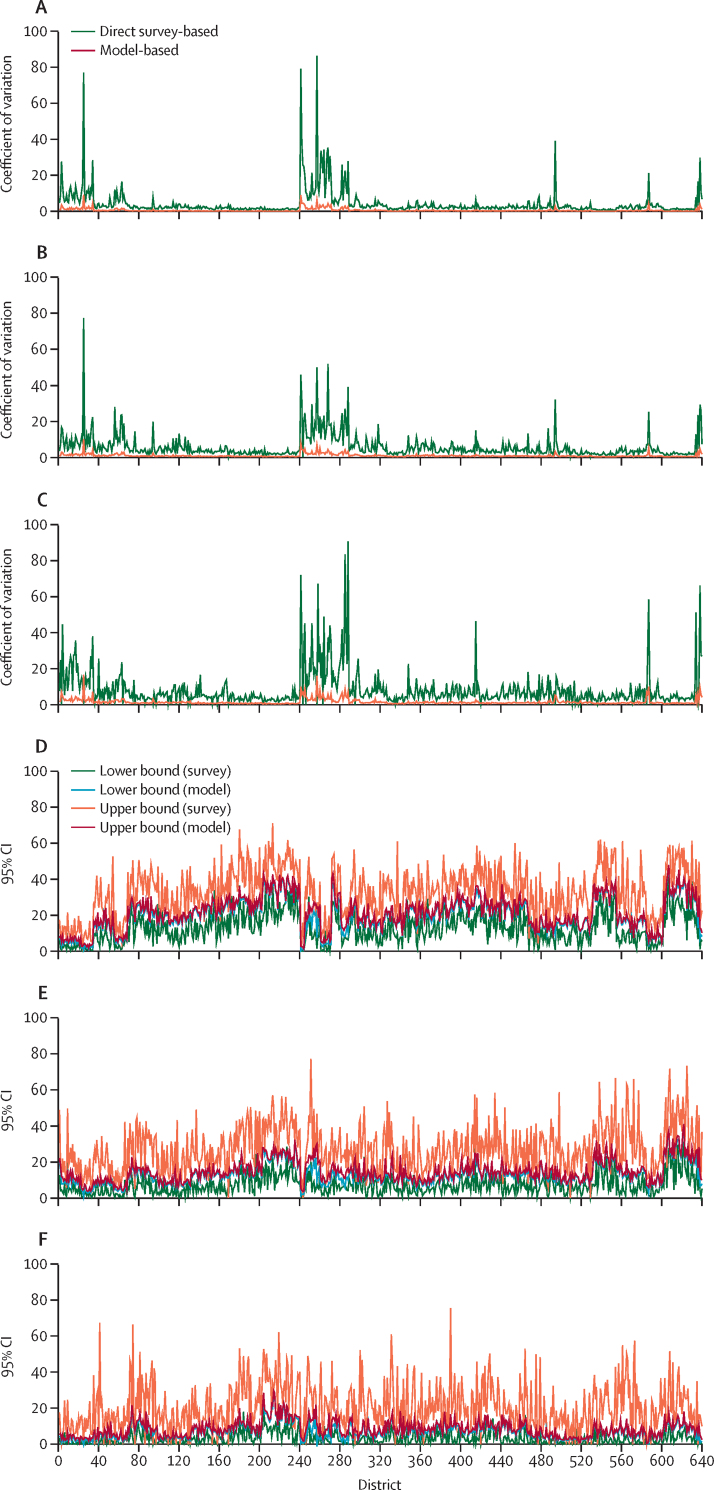
District-wise coefficients of variation and 95% CIs for model-based estimates and direct survey-based estimates of physical, emotional, and sexual violence experienced by women in the previous 12 months (India, 2015–16) (A) Physical violence. (B) Emotional violence. (C) Sexual violence. (D) Physical violence. (E) Emotional violence. (F) Sexual violence.

The coefficients of variation, and the fluctuations in the coefficients of variation of the direct survey-based estimates were larger than the coefficients of variation of the model-based estimates, indicating that the model-based estimates were more precise than the direct survey-based estimates (figure 3A–C). The direct survey-based estimates had wider 95% CIs than the model-based estimates, suggesting that the SEs of the direct survey-based estimates were large and unreliable (figure 3D–F). These diagnostics show that the model-based estimates were more robust than the direct survey-based estimates.

**Figure 4:**
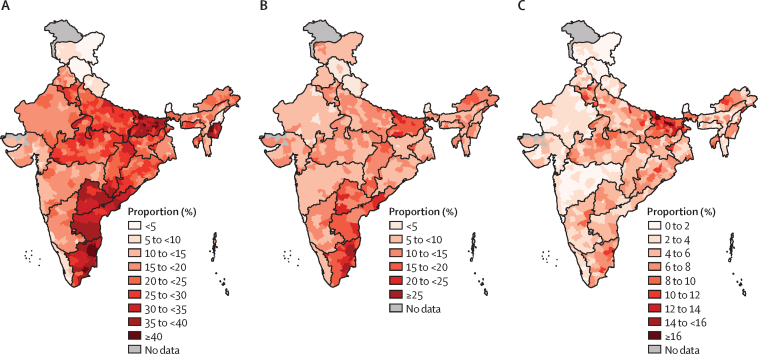
Model-based estimates of the proportion of physical, emotional, and sexual violence experienced by women in the previous 12 months (India, 2015–16) (A) Physical violence. (B) Emotional violence. (C) Sexual violence.

The model-based estimates of physical, emotional, and sexual IPV varied considerably across the 640 districts of India (figure 4). The estimated prevalence of physical IPV ranged from 1·1% (95% CI 0·7–1·4) in the East Sikkim district of Sikkim to 47·8% (47·3–48·2) in the Viluppuram district of Tamil Nadu. The estimated prevalence of emotional IPV ranged from 2·0% (0·5–3·5) in the Hamirpur district of Himanchal Pradesh to 30·2% (29·5–30·8) in the Sivaganga district of Tamil Nadu. The estimated prevalence of sexual IPV ranged from 0·1% (0·1–0·2) in North Goa to 18·1% (17·6–18·6) in the Saharsa district of Bihar.

We found considerable intrastate variations in IPV estimates (appendix pp 17–27). Regarding physical IPV, in Bihar, which had the highest state-level physical IPV prevalence (35·1%, 33·3–37·0), district-level estimates ranged from 23·5% (23·0–23·9) in Siwan to 42·7% (42·3–43·1) in Purbi Champaran. The state-level prevalence of physical IPV was lowest in Sikkim (1·8%, 0·7–4·6); district-level prevalence estimates ranged between 1·1% (0·7–1·4) in East Sikkim and 2·0% (1·3–2·7) in West Sikkim. Among larger states only, the lowest state-level prevalence of physical IPV was in Himanchal Pradesh (2·7%, 1·9–3·9); district-level prevalence estimates ranged between 1·6% (1·3–2·0) in Lahaul and Spiti and 3·9% (3·3–4·4) in Kullu. The state-level prevalence of physical IPV in Telangana (34·1%, 30·3–38·2) was similar to Bihar. The lowest district-level prevalence in Telangana was estimated in Hyderabad (27·9%, 27·5–28·3) and the highest district-level prevalence was estimated in Nizamabad (36·9%, 36·4–37·5).

Intrastate heterogeneity was also observed in prevalence estimates of emotional IPV. For example, in Tamil Nadu, with a state-level prevalence of 19·0% (17·4–20·8), the district-level prevalence estimates of emotional IPV ranged between 13·7% (13·2–14·1) in Kanniyakumari and 30·2% (29·5–30·8) in Sivaganga. In Bihar, with a state-level prevalence of emotional IPV of 18·1% (16·6–19·5), district-level prevalence estimates ranged between 12·4% (12·1–12·8) in Rohtas and 23·7% (23·3–24·2) in Aurangabad. The state-level prevalence of emotional IPV was lowest in Himanchal Pradesh (3·3%, 2·3–4·6), with district-level prevalence estimates ranging from 2·0% (0·5–3·5) in Hamirpur to 5·0% (4·4–5·6) in Kinnaur.

The state-level prevalence of sexual IPV in Bihar was 11·1% (9·9–12·4), the highest state-level prevalence of sexual IPV. District-level prevalence estimates ranged between 6·3% (6·1–6·6) in Siwan and 18·1% (17·6–18·6) in Saharsa. Among larger states only, the lowest state-level prevalence estimate of sexual IPV was estimated for Himanchal Pradesh (1·5%, 0·9–2·6); prevalence ranged between 0·7% (0·0–1·7) in Hamirpur and 2·3% (1·9–2·6) in Sirmaur.

Although the lowest state-level prevalence of physical IPV was in Himanchal Pradesh, variation across districts was the highest (0·223), as measured by coefficients of variation for the model-based estimates (appendix p 28). The National Capital Territory (NCT) of Delhi (0·213) and Jammu and Kashmir (0·200) were almost as high as Himanchal Pradesh in terms of variation in physical IPV across districts. Telangana had the lowest variation in physical IPV (0·096) across districts. The variation in emotional IPV was highest in West Bengal (0·358), then in Meghalaya (0·310) and Jammu and Kashmir (0·308). The lowest variation in emotional IPV across districts was in Bihar (0·134), then the NCT of Delhi (0·139), Manipur (0·145), and Punjab (0·156). For sexual IPV, the highest variation across districts was found in Nagaland (0·538), then in Arunachal Pradesh (0·454) and Punjab (0·451). The lowest variation in sexual IPV across districts was found in the NCT of Delhi (0·223).

**Figure 5:**
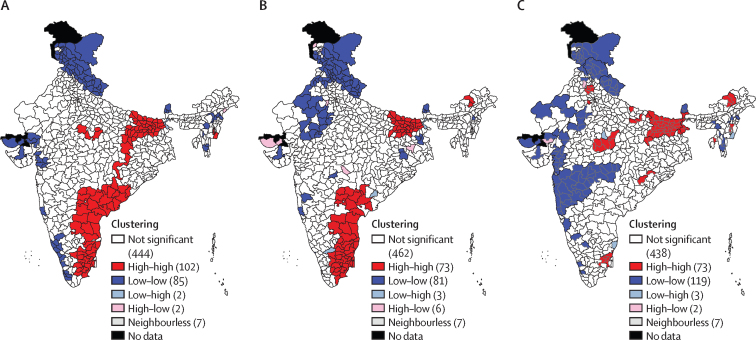
LISA cluster maps for the proportion of physical, emotional, and sexual violence experienced by women in the previous 12 months (India, 2015–16) (A) Physical violence. (B) Emotional violence. (C) Sexual violence. Numbers in keys indicate number of districts in each category. Neighbourless districts are districts that do not share boundaries with any other district of India. LISA=local indicators of spatial association.

We created univariate LISA maps to show the spatial heterogeneity of physical, emotional, and sexual IPV (figure 5). High–high spatial clusters of physical IPV were observed primarily in Andhra Pradesh, Bihar, Goa, Karnataka, Maharashtra, Chhattisgarh, Manipur, Tamil Nadu, and Uttar Pradesh. Some districts in Jharkhand, Madhya Pradesh, Odisha, and Puducherry also formed high–high spatial clusters of physical IPV. By contrast, coldspots (ie, clusters of districts that have a low prevalence of IPV in India) of physical IPV were located primarily in Jammu and Kashmir, Himanchal Pradesh, Assam, Gujarat, Karnataka, Kerala, Sikkim, Nagaland, Uttarakhand, and Mizoram.

High–high spatial clusters of emotional IPV were found in districts of Bihar, Andhra Pradesh, and Tamil Nadu and a few districts of Arunachal Pradesh, Karnataka, Puducherry, Chhattisgarh, and Uttar Pradesh. By contrast, low–low spatial clusters of emotional IPV were located in 81 districts, primarily from Jammu and Kashmir, Himanchal Pradesh, Sikkim, Punjab, Rajasthan, Uttarakhand, and a few districts of West Bengal, Puducherry, Nagaland, Maharashtra, Jharkhand, Haryana, and Gujarat. High–high clustering of sexual IPV was found primarily in districts of Bihar, Madhya Pradesh, Tamil Nadu, and Uttar Pradesh and a few districts of Arunachal Pradesh, Haryana, Jharkhand, Odisha, Puducherry, Manipur, and West Bengal. 119 districts showed low–low spatial clustering of sexual IPV; these districts primarily belonged to Gujarat, Himanchal Pradesh, Jammu and Kashmir, Rajasthan, Sikkim, and Uttarakhand and a few districts of Assam, Karnataka, Kerala, Mizoram, Meghalaya, and Nagaland.

## Discussion

Our secondary analysis of a cross-sectional, population-based survey conducted in 2015–16 provides unbiased, consistent, and reliable estimates of physical, emotional, and sexual IPV experienced by women in the previous 12 months in the 640 districts of India, and, to our knowledge, is the first to do so. We used area-level small-area estimation models, NFHS-4 survey data, and data from the 2011 Indian Census to generate these estimates, the calculation of which would be unreliable if directly estimated from NFHS surveys due to small sample sizes. Several diagnostic tests showed the power of small-area estimation to provide district-level estimates of physical, emotional, and sexual IPV in India. Our results also show the spatial heterogeneity in IPV prevalence across the 640 districts of India and the power of small-area estimation to identify hotspots with larger burdens of IPV. In the absence of complete civil-registration and vital-registration systems and little readily available data at local levels, many low-income and middle-income countries rely solely on household survey data to estimate indicators. This analysis offers an example of how commonly available household survey data (eg, the NFHS or the Indian implementation of the Demographic and Health Surveys) can be combined with national census data to estimate key metrics (eg, IPV, the empowerment of women, and the digital and financial inclusion of women) that have, to date, been limited to more aggregate assessments.

Existing demographic studies show a clear divide between the north and the south of India on indicators of patriarchy, the empowerment of women, and son preference.^22^, ^23^, ^24^ Our findings do not indicate any such divide for IPV. The districts with the highest prevalence of physical, emotional, and sexual IPV were spread across central, eastern, and southern India. Clusters of districts with high amounts of physical IPV transgressed the traditional administrative boundaries that are represented in state-level analyses, with examples seen for the borders between Bihar and Jharkhand, Bihar and Uttar Pradesh, Chhattisgarh and Telangana, Odisha and Andhra Pradesh, Odisha and Telangana, and others. Similarly, clusters of districts with high amounts of emotional IPV appeared to be concentrated around state borders, with this seen for the borders between Bihar and Jharkhand, Bihar and Uttar Pradesh, and Chhattisgarh and Telangana. This geographical heterogeneity emphasises the importance of substate estimation of IPV to be able to track fluctuations at more granular levels due to the lack of consistency at state and regional levels, as well as suggesting that state and regional boundaries might be inadequate to understand and represent IPV across India.

Comparing our findings with previous research reveals considerable overlap in hotspots of physical, emotional, and sexual IPV and their key predictors. For example, hotspots of physical, emotional, and sexual IPV in Bihar, the state with the highest prevalence of physical and sexual IPV, overlapped with hotspots of patriarchy and alcohol use among men.^23^ The majority of hotspots of physical and emotional IPV in Telangana, Andhra Pradesh, and Tamil Nadu overlapped with increased alcohol use among men. A hotspot of physical IPV in Manipur also overlapped with a hotspot of alcohol use among men. Several hotspots of sexual IPV overlapped with hotspots of alcohol use among men in Bihar, Arunachal Pradesh, Manipur, and Tamil Nadu. A few hotspots of physical, emotional, and sexual IPV in Bihar, Telangana, and Andhra Pradesh also overlapped with increased rates of child marriage.^25^ These confluences suggest that the geographical clustering of IPV in India aligns with many of the demographic determinants of IPV (eg, social and cultural norms surrounding kinship, marriage systems, and behaviours such as alcohol use) that promote and sustain violence. Identifying overlapping hotspots offers a unique opportunity to highlight districts with the greatest need for IPV prevention and mitigation support.

As the intrastate and interstate prevalence of the three forms of IPV are not uniformly distributed, and as predictors of IPV might also vary at the district level, any national-level or state-level interventions might not have the granularity needed for appropriate services, resources, and programmes that are adapted to the needs of each district. Our findings offer a direct way to inform the work of policy makers and programme managers to incorporate the consideration of local culture, context, and narratives of gender more directly to address the burden of IPV in India.

A key limitation of this analysis is that IPV data are self-reported, so there might be reporting bias. Furthermore, this analysis could not be replicated with the most recent NFHS data, which had data collection during 2019–21 and was interrupted by COVID-19 lockdowns. There are reports suggesting that the COVID-19 pandemic and its associated non-pharmaceutical responses could have affected the prevalence and reporting of IPV.^26^ Some reports indicate that IPV in India might have been exacerbated by the COVID-19 pandemic,^26^ whereas NFHS-5 data indicate that the prevalence of physical or sexual IPV among women who had ever been married in India declined from 31% in NFHS-4 to 29% in NFHS-5.^5^ A majority of states and union territories, irrespective of whether they were surveyed before COVID-19 lockdowns started in India or after the COVID-19 lockdowns ended, registered a decline in physical or sexual IPV between NFHS-4 and NFHS-5. There are no reliable means of estimating the effects of the COVID-19 pandemic on IPV prevalence and reporting at the district level with NFHS data. Our scatterplot of residual versus model-based estimates for sexual IPV deviated from the assumption of constant variance. We tried several data-transformation techniques to address heteroskedasticity in our dataset, but none eliminated the problem fully. The presence of heteroskedasticity does not cause bias in the estimates; however, it increases the variance of the estimates.^27^ Therefore, the estimates of sexual IPV should be interpreted with caution. Despite these limitations, our study provides compelling district-level estimates of IPV in India.

There is increasing literature and knowledge on ways to stop IPV. Programmes that offer cash transfers to women, bolster income-generation opportunities for women, transform attitudes justifying violence against women, and reduce exposure to violence and abuse during childhood are integral violence-prevention strategies in several countries.^28^, ^29^ The Government of India has launched several schemes to support skill development and employment opportunities for women, including the establishment of Mahila Shakti Kendra in the 115 least developed districts^30^ and the Support to Training and Employment Programme.^31^ Such schemes will be better positioned to accelerate progress if indicators such as IPV are considered when identifying implementation districts.

As well as the direct policy and programme applications for IPV prevention and mitigation, our analysis allows for exploration of the relationship between IPV and other important health, social, and developmental factors at local levels, including gender inequality, consumption of alcohol or other substances, and natural disasters. Future research should consider the use of small-area estimation to estimate the prevalence of IPV at regular intervals for monitoring the effectiveness of policy and programme interventions that are meant to reduce IPV in specific locations. Another important use of this technique in future research is a district-level analysis of the confluences of different types of IPV to identify and support districts that contain multiple burdens of IPV against women.

## Data sharing

National Family Health Survey data are currently available on request from the Demographic and Health Surveys website (https://dhsprogram.com/methodology/survey/survey-display-355.cfm). Census 2011 data can be downloaded from the Office of the Registrar General and Census Commissioner website (https://censusindia.gov.in/census.website). No additional, related documents will be available.

## Declaration of interests

We declare no competing interests.
