# Supplementary material for: Spatial heterogeneity in intimate partner violence across the 640 districts of India: a secondary analysis of a cross-sectional, population-based survey by use of model-based small-area estimation
Source: Lancet Glob Health. 2023 Sep 19;11(10):e1587–97. doi: 10.1016/S2214-109X(23)00377-7 (PMC10522783; doi:10.1016/S2214-109X(23)00377-7)

THE LANCET
Global Health

Supplementary appendix

This appendix formed part of the original submission and has been peer reviewed. We post it as supplied by the authors.

Supplement to: Srivastava S, Kumar K, McDougal L, et al. Spatial heterogeneity in intimate partner violence across the 640 districts of India: a secondary analysis of a cross-sectional, population-based survey by use of model-based small-area estimation. *Lancet Glob Health* 2023; **11:** e1587–97.

Contents of Appendix

Estimation of outcome variables	page 1
Definition of auxiliary variables	page 2
Diagnostic measures	page 3
LISA	Page 4
Table A1	Page 5-16
Table A2	Page 17-27
Table A3	Page 28
Figure A1	Page 29

Appendix

Estimation of outcome variables

The NFHS-4 asked the following questions to women who were administered domestic violence module to capture experience of physical IPV:

(Does/Did) your (last) husband ever do any of the following things to you:

a. Push you, shake you, or throw something at you?
b. Twist your arm or pull your hair?
c. Slap you?
d. Punch you with his fist or with something that could hurt you?
e. Kick you, drag you or beat you up?
f. Try to choke you or burn you on purpose?
g. Threaten or attack you with a knife, gun, or any other weapon?

If yes to any of the above, the women were further asked:

How often did this happen during the last 12 months: often, only sometimes, or not at all?

Women who reported “often” or “only sometimes” to one or more of above things were coded as having experienced physical IPV in the last 12 months.

Similarly, the NFHS-4 asked the following questions to women who were administered domestic violence module to capture experience of sexual IPV:

(Does/Did) your (last) husband ever do any of the following things to you:

a. Physically force you to have sexual intercourse with him even when you did not want to?
b. Physically force you to perform any other sexual acts you did not want to?
c. Force you with threats or in any other way to perform sexual acts you did not want to?

If yes to any of the above, the women were further asked:

How often did this happen during the last 12 months: often, only sometimes, or not at all?

Women who reported “often” or “only sometimes” to one or more of the above things were coded as having experienced sexual IPV in the last 12 months.

For capturing emotional violence, the NFHS-4 asked following questions:

Now if you will permit me, I need to ask some more questions about your relationship with your (last) husband. (Does/Did) your (last) husband ever:

a. Say or do something to humiliate you in front of others?
b. Threaten to hurt or harm you or someone close to you?
c. Insult you or make you feel bad about yourself?

If yes to any of the above, the women were further asked:
How often did this happen during the last 12 months: often, only sometimes, or not at all?

Women who reported "often" or "only sometimes" to one or more of the above things were coded as having experienced emotional IPV in the last 12 months.

We finally aggregated the women's experience of three forms of IPV at the district-level to estimate the district-level prevalence of the three forms of IPV.

Definition of auxiliary variables

Auxiliary variables were derived from the 2011 Indian Census (latest available Indian Census). The details of the auxiliary variables are given below:

a. Female workforce participation: The percentage of women age 15-49 who worked as main- or marginal- worker
b. Gender gap relative to male workforce participation: The difference between percentage of male workforce participation and female workforce participation
c. Female literacy: Percentage of women age 15-49 who were literate
d. Gender gap relative to male literacy: The difference between percentage of male and female literates
e. Female headed household: Percentage of households headed by a female member
f. Male migration in past 12 months: Percentage of male migrants in the last 12 months
g. Female mean age at marriage
h. Birth of a male child in last one year: Percentage of male births in last one year
i. Sex ratio at birth: Female births per 1000 male births
j. Mean household size in a district
k. Urban residence: Percentage of population residing in urban areas
l. Scheduled castes/tribes: Percentage of households belonging to the scheduled castes/tribes
m. Muslim: Percentage of households belonging to Muslim religion

n. Socio-economic status (SES)
o. Border Proximity Factor (BPF)

Estimation of district-level socio-economic status

To construct district-specific SES, we used consumer durables (radio, television, bicycle, motorcycle, car, telephone) and housing characteristics (type of wall, roof, floor, availability of electricity, sources of drinking water, toilet facility, availability of separate kitchen, clean fuel for cooking) (Filmer & Pritchett, 2001). Scores from these items were derived using principal components analysis at district-level, and further categorized into deciles, from lowest to highest; the lowest 10% of districts out of 640 districts are categorized as being in the lowest SES, second 10% of the districts being in the second lowest SES, and the highest 10% of districts are in the highest SES.

Border Proximity Factor (BPF)

The Border Proximity Factor (BPF) determines the direction and distance of districts from state borders, which is based on the mean euclidean distance. Along with considering district's proximity to the state borders, euclidean distance also considers the area of a district that actually has a greater proximity to the state border (longer the area connected to the border longer will be the proximity factor). Also, if a district in one state has connected borders with multiple districts of other states it will have a higher proximity score. In order to estimate the BPF, we first digitized the georeferenced state borders and created a raster surface of the distance of districts from the state borders. We then aggregated the proximity data for each district by taking the mean euclidean distance of districts from the state borders.

Diagnostic measures

The model diagnostic is used to verify the assumptions of the underlying model. Under the GLMM framework, the random area specific effects are assumed to follow a normal distribution with mean zero and a constant variance. If the model assumptions are upheld, then the area (district) level residuals are expected to be randomly distributed and not significantly different from the line y=0[18].

The diagnostics for small-area estimates are used to validate the reliability of model-based small area estimates obtained from GLMM models. These diagnostics include a) bias diagnostic, b) coefficient of variation (CV), and c) 95% confidence intervals (CIs) of model-based estimates[18]. The bias diagnostic is used to examine the deviation of the model-based district-level estimates from the direct survey estimates to validate the reliability of the model-based district-level estimates. The CVs are used to assess the improvement in the precision of the model-based estimates over the direct survey-based estimates.

Estimates with low CV are considered more reliable. The 95% CIs of the model-based estimates and direct survey-based estimates are compared to validate the robustness of the model-based estimates.

LISA

LISA measures the correlation of neighborhood values around a specific spatial location. It determines the extent of spatial non-stationery and clustering present in the data. It is given by

$$Moran's\, I = C \;\; X \;\; \frac{\sum_{ij} w_{ij} z_i z_j}{\sum_i z_i^2}$$

Z_i: standardized variable of interest;

W_{ij}: weight matrix;

LISA shows high-high clustering (high prevalent districts surrounded by high prevalent neibhourhood), low-low clustering (low prevalent districts surrounded by low prevalent neighborhood), and spatial outliers (low–high and high-low clusters). The high-high clusters are also called hot spots representing districts with high IPV surrounded by districts with high IPV. The low-low clusters are termed as cold spots and characterized by low IPV districts surrounded by low IPV districts. The districts marked as not significant are those which are surrounded by districts with different patterns of IPV. Queen's contiguity weight matrix was used for estimating LISA.

Table A1: District's proximity from state borders for India, 2011

State name	District name	Border Proximity Factor
Jammu & Kashmir	Jammu & Kashmir	0.956
Jammu & Kashmir	Kupwara	0.165
Jammu & Kashmir	Badgam	0.417
Jammu & Kashmir	Leh (Laddakh)	0.891
Jammu & Kashmir	Kargil	0.340
Jammu & Kashmir	Punch	0.110
Jammu & Kashmir	Rajouri	0.209
Jammu & Kashmir	Kathua	0.126
Jammu & Kashmir	Baramula	0.272
Jammu & Kashmir	Bandipore	0.182
Jammu & Kashmir	Srinagar	0.488
Jammu & Kashmir	Ganderbal	0.322
Jammu & Kashmir	Pulwama	0.679
Jammu & Kashmir	Shupiyan	0.599
Jammu & Kashmir	Anantnag	0.679
Jammu & Kashmir	Kulgam	0.681
Jammu & Kashmir	Doda	0.287
Jammu & Kashmir	Ramban	0.747
Jammu & Kashmir	Kishtwar	0.431
Jammu & Kashmir	Udhampur	0.448
Jammu & Kashmir	Reasi	0.515
Jammu & Kashmir	Jammu	0.347
Jammu & Kashmir	Samba	0.348
Himanchal Pradesh	Chamba	0.282
Himanchal Pradesh	Kangra	0.380
Himanchal Pradesh	Lahaul & Spiti	0.292
Himanchal Pradesh	Kullu	0.704
Himanchal Pradesh	Mandi	0.575
Himanchal Pradesh	Hamirpur	0.310
Himanchal Pradesh	Una	0.100
Himanchal Pradesh	Bilaspur	0.157
Himanchal Pradesh	Solan	0.142
Himanchal Pradesh	Sirmaur	0.129
Himanchal Pradesh	Shimla	0.258
Himanchal Pradesh	Kinnaur	0.355
Punjab	Gurdaspur	0.259
Punjab	Kapurthala	0.666
Punjab	Jalandhar	0.594
Punjab	Hoshiarpur	0.142
Punjab	Shahid Bhagat Singh Nagar	0.231
Punjab	Fatehgarh Sahib	0.342
Punjab	Ludhiana	0.610
Punjab	Moga	0.840
Punjab	Firozpur	0.547
Punjab	Muktsar	0.342
Punjab	Faridkot	0.645
Punjab	Bathinda	0.266
Punjab	Mansa	0.150
Punjab	Patiala	0.132
Punjab	Amritsar	0.757
Punjab	Tarn Taran	1.038
Punjab	Rupnagar	0.118
Punjab	Sahibzada Ajit Singh Nagar	0.068
Punjab	Sangrur	0.318
Punjab	Barnala	0.588
Chandigarh	Chandigarh	0.015

Continued…

Table A1: District's proximity from state borders for India, 2011-*Continued*		
State name	District name	Border Proximity Factor
Uttarakhand	Uttarkashi	0.244
Uttarakhand	Chamoli	0.860
Uttarakhand	Rudraprayag	0.597
Uttarakhand	Tehri Garhwal	0.488
Uttarakhand	Dehradun	0.152
Uttarakhand	Garhwal	0.318
Uttarakhand	Pithoragarh	1.256
Uttarakhand	Bageshwar	1.063
Uttarakhand	Almora	0.600
Uttarakhand	Champawat	0.466
Uttarakhand	Nainital	0.304
Uttarakhand	Udham Singh Nagar	0.079
Uttarakhand	Hardwar	0.100
Haryana	Panchkula	0.044
Haryana	Ambala	0.093
Haryana	Yamunanagar	0.090
Haryana	Kurukshetra	0.177
Haryana	Kaithal	0.199
Haryana	Karnal	0.224
Haryana	Panipat	0.202
Haryana	Sonipat	0.229
Haryana	Jind	0.385
Haryana	Fatehabad	0.125
Haryana	Sirsa	0.091
Haryana	Hisar	0.324
Haryana	Bhiwani	0.299
Haryana	Rohtak	0.428
Haryana	Jhajjar	0.211
Haryana	Mahendragarh	0.089
Haryana	Rewari	0.124
Haryana	Gurgaon	0.105
Haryana	Mewat	0.065
Haryana	Faridabad	0.067
Haryana	Palwal	0.136
NCT Of Delhi	North West	0.053
NCT Of Delhi	North	0.058
NCT Of Delhi	North East	0.019
NCT Of Delhi	East	0.025
NCT Of Delhi	New Delhi	0.097
NCT Of Delhi	Central	0.096
NCT Of Delhi	West	0.095
NCT Of Delhi	South West	0.045
NCT Of Delhi	South	0.032
Rajasthan	Ganganagar	0.708
Rajasthan	Hanumangarh	0.255
Rajasthan	Bikaner	1.855
Rajasthan	Churu	0.846
Rajasthan	Jhunjhunun	0.339
Rajasthan	Alwar	0.280
Rajasthan	Bharatpur	0.152
Rajasthan	Dhaulpur	0.079
Rajasthan	Karauli	0.311
Rajasthan	Sawai Madhopur	0.371
Rajasthan	Dausa	0.743
Rajasthan	Jaipur	0.918

Continued…

Table A1: District's proximity from state borders for India, 2011-*Continued*

State name	District name	Border Proximity Factor
Rajasthan	Sikar	0.745
Rajasthan	Nagaur	1.855
Rajasthan	Jodhpur	2.154
Rajasthan	Jaisalmer	2.414
Rajasthan	Barmer	1.058
Rajasthan	Jalor	0.483
Rajasthan	Sirohi	0.307
Rajasthan	Pali	1.223
Rajasthan	Ajmer	1.245
Rajasthan	Tonk	0.896
Rajasthan	Bundi	0.474
Rajasthan	Bhilwara	0.588
Rajasthan	Rajsamand	0.920
Rajasthan	Dungarpur	0.246
Rajasthan	Banswara	0.197
Rajasthan	Chittaurgarh	0.219
Rajasthan	Kota	0.276
Rajasthan	Baran	0.148
Rajasthan	Jhalawar	0.130
Rajasthan	Udaipur	0.441
Rajasthan	Pratapgarh	0.170
Uttar Pradesh	Saharanpur	0.104
Uttar Pradesh	Muzaffarnagar	0.221
Uttar Pradesh	Bijnor	0.207
Uttar Pradesh	Moradabad	0.437
Uttar Pradesh	Rampur	0.248
Uttar Pradesh	Jyotiba Phule Nagar	0.622
Uttar Pradesh	Meerut	0.471
Uttar Pradesh	Baghpat	0.130
Uttar Pradesh	Ghaziabad	0.366
Uttar Pradesh	Gautam Buddha Nagar	0.096
Uttar Pradesh	Bulandshahr	0.506
Uttar Pradesh	Aligarh	0.568
Uttar Pradesh	Mahamaya Nagar	0.572
Uttar Pradesh	Mathura	0.162
Uttar Pradesh	Agra	0.122
Uttar Pradesh	Firozabad	0.348
Uttar Pradesh	Mainpuri	0.493
Uttar Pradesh	Budaun	0.908
Uttar Pradesh	Bareilly	0.432
Uttar Pradesh	Pilibhit	0.258
Uttar Pradesh	Shahjahanpur	0.800
Uttar Pradesh	Kheri	0.932
Uttar Pradesh	Sitapur	1.506
Uttar Pradesh	Hardoi	1.269
Uttar Pradesh	Unnao	1.198
Uttar Pradesh	Lucknow	1.562
Uttar Pradesh	Rae Bareli	1.051
Uttar Pradesh	Farrukhabad	0.902
Uttar Pradesh	Kannauj	0.773
Uttar Pradesh	Etawah	0.175
Uttar Pradesh	Auraiya	0.435
Uttar Pradesh	Kanpur Dehat	0.738
Uttar Pradesh	Kanpur Nagar	0.892
Uttar Pradesh	Jalaun	0.338

Continued…

Table A1: District's proximity from state borders for India, 2011-*Continued*

State name	District name	Border Proximity Factor
Uttar Pradesh	Jhansi	0.101
Uttar Pradesh	Lalitpur	0.087
Uttar Pradesh	Hamirpur	0.403
Uttar Pradesh	Mahoba	0.115
Uttar Pradesh	Banda	0.230
Uttar Pradesh	Chitrakoot	0.118
Uttar Pradesh	Fatehpur	0.627
Uttar Pradesh	Pratapgarh	0.765
Uttar Pradesh	Kaushambi	0.323
Uttar Pradesh	Allahabad	0.324
Uttar Pradesh	Bara Banki	1.739
Uttar Pradesh	Faizabad	1.535
Uttar Pradesh	Ambedkar Nagar	1.237
Uttar Pradesh	Sultanpur	1.198
Uttar Pradesh	Bahraich	1.738
Uttar Pradesh	Shrawasti	1.958
Uttar Pradesh	Balrampur	1.499
Uttar Pradesh	Gonda	1.774
Uttar Pradesh	Siddharth nagar	0.993
Uttar Pradesh	Basti	1.296
Uttar Pradesh	Sant Kabir Nagar	0.902
Uttar Pradesh	Mahrajganj	0.367
Uttar Pradesh	Gorakhpur	0.581
Uttar Pradesh	Kushinagar	0.144
Uttar Pradesh	Deoria	0.152
Uttar Pradesh	Azamgarh	0.807
Uttar Pradesh	Mau	0.475
Uttar Pradesh	Ballia	0.115
Uttar Pradesh	Jaunpur	0.887
Uttar Pradesh	Ghazipur	0.277
Uttar Pradesh	Chandauli	0.149
Uttar Pradesh	Varanasi	0.500
Uttar Pradesh	Sant Ravidas Nagar (Bhadohi)	0.608
Uttar Pradesh	Mirzapur	0.285
Uttar Pradesh	Sonbhadra	0.138
Uttar Pradesh	Etah	0.700
Uttar Pradesh	Kanshiram Nagar	0.987
Bihar	Pashchim Champaran	0.232
Bihar	Purba Champaran	0.540
Bihar	Sheohar	0.886
Bihar	Sitamarhi	1.125
Bihar	Madhubani	1.491
Bihar	Supaul	1.054
Bihar	Araria	0.537
Bihar	Kishanganj	0.148
Bihar	Purnia	0.365
Bihar	Katihar	0.145
Bihar	Madhepura	0.747
Bihar	Saharsa	0.949
Bihar	Darbhanga	1.280
Bihar	Muzaffarpur	0.812
Bihar	Gopalganj	0.174
Bihar	Siwan	0.174
Bihar	Saran	0.274
Bihar	Vaishali	0.748
Bihar	Samastipur	0.978

Continued…

Table A1: District's proximity from state borders for India, 2011-*Continued*

State name	District name	Border Proximity Factor
Bihar	Begusarai	0.730
Bihar	Khagaria	0.717
Bihar	Bhagalpur	0.233
Bihar	Banka	0.167
Bihar	Munger	0.571
Bihar	Lakhisarai	0.400
Bihar	Sheikhpura	0.326
Bihar	Nalanda	0.522
Bihar	Patna	0.536
Bihar	Bhojpur	0.245
Bihar	Buxar	0.116
Bihar	Kaimur (Bhabua)	0.142
Bihar	Rohtas	0.301
Bihar	Aurangabad	0.279
Bihar	Gaya	0.229
Bihar	Nawada	0.143
Bihar	Jamui	0.163
Bihar	Jehanabad	0.630
Bihar	Arwal	0.543
Sikkim	North District	0.568
Sikkim	West District	0.181
Sikkim	South District	0.163
Sikkim	East District	0.132
Arunachal Pradesh	Tawang	0.776
Arunachal Pradesh	West Kameng	0.340
Arunachal Pradesh	East Kameng	0.458
Arunachal Pradesh	Papum Pare	0.230
Arunachal Pradesh	Upper Subansiri	0.763
Arunachal Pradesh	West Siang	0.660
Arunachal Pradesh	East Siang	0.295
Arunachal Pradesh	Upper Siang	1.040
Arunachal Pradesh	Changlang	0.419
Arunachal Pradesh	Tirap	0.124
Arunachal Pradesh	Lower Subansiri	0.239
Arunachal Pradesh	Kurung Kumey	0.840
Arunachal Pradesh	Dibang Valley	1.012
Arunachal Pradesh	Lower Dibang Valley	0.428
Arunachal Pradesh	Lohit	0.288
Arunachal Pradesh	Anjaw	0.885
Nagaland	Mon	0.126
Nagaland	Mokokchung	0.148
Nagaland	Zunheboto	0.316
Nagaland	Wokha	0.131
Nagaland	Dimapur	0.058
Nagaland	Phek	0.123
Nagaland	Tuensang	0.465
Nagaland	Longleng	0.211
Nagaland	Kiphire	0.319
Nagaland	Kohima	0.158
Nagaland	Peren	0.068
Manipur	Senapati	0.269
Manipur	Tamenglong	0.182
Manipur	Churachandpur	0.289
Manipur	Bishnupur	0.635
Manipur	Thoubal	0.728
Manipur	Imphal West	0.046

Continued…

Table A1: District's proximity from state borders for India, 2011-*Continued*

State name	District name	Border Proximity Factor
Manipur	Imphal East	0.520
Manipur	Ukhrul	0.472
Manipur	Chandel	0.844
Mizoram	Mamit	0.204
Mizoram	Kolasib	0.110
Mizoram	Aizawl	0.312
Mizoram	Champhai	0.505
Mizoram	Serchhip	0.776
Mizoram	Lunglei	0.887
Mizoram	Lawngtlai	1.412
Mizoram	Saiha	1.540
Tripura	West Tripura	0.796
Tripura	South Tripura	0.771
Tripura	Dhalai	0.345
Tripura	North Tripura	0.106
Meghalaya	West Garo Hills	0.132
Meghalaya	East Garo Hills	0.167
Meghalaya	South Garo Hills	0.131
Meghalaya	West Khasi Hills	0.179
Meghalaya	Ribhoi	0.087
Meghalaya	East Khasi Hills	0.181
Meghalaya	Jaintia Hills	0.107
Assam	Kokrajhar	0.272
Assam	Dhubri	0.122
Assam	Goalpara	0.097
Assam	Barpeta	0.418
Assam	Morigaon	0.236
Assam	Nagaon	0.448
Assam	Sonitpur	0.175
Assam	Lakhimpur	0.179
Assam	Dhemaji	0.101
Assam	Tinsukia	0.119
Assam	Dibrugarh	0.243
Assam	Sivasagar	0.144
Assam	Jorhat	0.209
Assam	Golaghat	0.203
Assam	Karbi Anglong	0.297
Assam	Dima Hasao	0.159
Assam	Cachar	0.135
Assam	Karimganj	0.094
Assam	Hailakandi	0.114
Assam	Bongaigaon	0.391
Assam	Chirang	0.636
Assam	Kamrup	0.183
Assam	Kamrup Metropolitan	0.056
Assam	Nalbari	0.402
Assam	Baksa	0.637
Assam	Darrang	0.310
Assam	Udalguri	0.222
West Bengal	Darjiling	0.110
West Bengal	Jalpaiguri	0.228
West Bengal	Koch Bihar	0.095
West Bengal	Uttar Dinajpur	0.060
West Bengal	Dakshin Dinajpur	0.088
West Bengal	Maldah	0.094
West Bengal	Murshidabad	0.214

Continued…

Table A1: District's proximity from state borders for India, 2011-Continued

State name	District name	Border Proximity Factor
West Bengal	Birbhum	0.191
West Bengal	Barddhaman	0.495
West Bengal	Nadia	0.159
West Bengal	North Twenty Four Parganas	0.194
West Bengal	Hugli	0.715
West Bengal	Bankura	0.476
West Bengal	Puruliya	0.116
West Bengal	Haora	0.824
West Bengal	Kolkata	0.581
West Bengal	South Twenty Four Parganas	0.576
West Bengal	Paschim Medinipur	0.358
West Bengal	Purba Medinipur	0.443
Jharkhand	Garhwa	0.125
Jharkhand	Chatra	0.218
Jharkhand	Kodarma	0.146
Jharkhand	Giridih	0.244
Jharkhand	Deoghar	0.180
Jharkhand	Godda	0.148
Jharkhand	Sahibganj	0.116
Jharkhand	Pakur	0.130
Jharkhand	Dhanbad	0.189
Jharkhand	Bokaro	0.183
Jharkhand	Lohardaga	0.563
Jharkhand	Purbi Singhbhum	0.079
Jharkhand	Palamu	0.212
Jharkhand	Latehar	0.371
Jharkhand	Hazaribagh	0.455
Jharkhand	Ramgarh	0.357
Jharkhand	Dumka	0.166
Jharkhand	Jamtara	0.135
Jharkhand	Ranchi	0.451
Jharkhand	Khunti	0.469
Jharkhand	Gumla	0.266
Jharkhand	Simdega	0.121
Jharkhand	Pashchimi Singhbhum	0.195
Jharkhand	Saraikela-Kharsawan	0.178
Odisha	Bargarh	0.183
Odisha	Jharsuguda	0.332
Odisha	Sambalpur	0.703
Odisha	Debagarh	0.659
Odisha	Sundargarh	0.178
Odisha	Kendujhar	0.485
Odisha	Mayurbhanj	0.267
Odisha	Baleshwar	0.421
Odisha	Bhadrak	0.974
Odisha	Kendrapara	1.358
Odisha	Jagatsinghapur	1.729
Odisha	Cuttack	1.479
Odisha	Jajapur	1.141
Odisha	Dhenkanal	1.176
Odisha	Anugul	1.065
Odisha	Nayagarh	1.118
Odisha	Khordha	1.239
Odisha	Puri	1.247
Odisha	Ganjam	0.516

Continued…

Table A1: District's proximity from state borders for India, 2011-Continued

State name	District name	Border Proximity Factor
Odisha	Gajapati	0.268
Odisha	Kandhamal	0.966
Odisha	Baudh	1.040
Odisha	Subarnapur	0.610
Odisha	Balangir	0.480
Odisha	Nuapada	0.130
Odisha	Kalahandi	0.460
Odisha	Rayagada	0.316
Odisha	Nabarangapur	0.171
Odisha	Koraput	0.176
Odisha	Malkangiri	0.097
Chhattisgarh	Koriya	0.174
Chhattisgarh	Surguja	0.396
Chhattisgarh	Jashpur	0.207
Chhattisgarh	Raigarh	0.272
Chhattisgarh	Korba	0.660
Chhattisgarh	Janjgir - Champa	0.654
Chhattisgarh	Bilaspur	0.399
Chhattisgarh	Kabeerdham	0.184
Chhattisgarh	Rajnandgaon	0.192
Chhattisgarh	Durg	0.628
Chhattisgarh	Raipur	0.436
Chhattisgarh	Mahasamund	0.184
Chhattisgarh	Dhamtari	0.440
Chhattisgarh	Uttar Bastar Kanker	0.389
Chhattisgarh	Bastar	0.270
Chhattisgarh	Narayanpur	0.279
Chhattisgarh	Dakshin Bastar Dantewada	0.264
Chhattisgarh	Bijapur	0.216
Madhya Pradesh	Sheopur	0.196
Madhya Pradesh	Morena	0.158
Madhya Pradesh	Bhind	0.188
Madhya Pradesh	Gwalior	0.418
Madhya Pradesh	Datia	0.185
Madhya Pradesh	Shivpuri	0.254
Madhya Pradesh	Tikamgarh	0.108
Madhya Pradesh	Chhatarpur	0.254
Madhya Pradesh	Panna	0.604
Madhya Pradesh	Sagar	0.420
Madhya Pradesh	Damoh	0.799
Madhya Pradesh	Satna	0.400
Madhya Pradesh	Rewa	0.208
Madhya Pradesh	Umaria	0.645
Madhya Pradesh	Neemuch	0.106
Madhya Pradesh	Mandsaur	0.139
Madhya Pradesh	Ratlam	0.195
Madhya Pradesh	Ujjain	0.480
Madhya Pradesh	Shajapur	0.479
Madhya Pradesh	Dewas	0.980
Madhya Pradesh	Dhar	0.623
Madhya Pradesh	Indore	1.007
Madhya Pradesh	West Nimar (Khargone)	0.543
Madhya Pradesh	Barwani	0.214
Madhya Pradesh	Rajgarh	0.310

Continued…

Table A1: District's proximity from state borders for India, 2011-Continued		
State name	District name	Border Proximity Factor
Madhya Pradesh	Vidisha	0.634
Madhya Pradesh	Bhopal	0.834
Madhya Pradesh	Sehore	1.032
Madhya Pradesh	Raisen	1.078
Madhya Pradesh	Betul	0.301
Madhya Pradesh	Harda	0.515
Madhya Pradesh	Hoshangabad	0.936
Madhya Pradesh	Katni	1.095
Madhya Pradesh	Jabalpur	1.282
Madhya Pradesh	Narsimhapur	1.161
Madhya Pradesh	Dindori	0.454
Madhya Pradesh	Mandla	0.637
Madhya Pradesh	Chhindwara	0.507
Madhya Pradesh	Seoni	0.680
Madhya Pradesh	Balaghat	0.227
Madhya Pradesh	Guna	0.202
Madhya Pradesh	Ashoknagar	0.321
Madhya Pradesh	Shahdol	0.244
Madhya Pradesh	Anuppur	0.213
Madhya Pradesh	Sidhi	0.268
Madhya Pradesh	Singrauli	0.168
Madhya Pradesh	Jhabua	0.171
Madhya Pradesh	Alirajpur	0.137
Madhya Pradesh	East Nimar (Khandwa)	0.444
Madhya Pradesh	Burhanpur	0.130
Gujarat	Kachchh	0.616
Gujarat	Banas Kantha	0.296
Gujarat	Patan	0.757
Gujarat	Mahesana	0.794
Gujarat	Sabar Kantha	0.277
Gujarat	Gandhinagar	0.860
Gujarat	Ahmadabad	1.513
Gujarat	Surendranagar	1.611
Gujarat	Rajkot	1.963
Gujarat	Jamnagar	1.953
Gujarat	Porbandar	2.530
Gujarat	Junagadh	2.413
Gujarat	Amreli	1.989
Gujarat	Bhavnagar	1.728
Gujarat	Anand	1.183
Gujarat	Kheda	0.886
Gujarat	Panch Mahals	0.377
Gujarat	Dohad	0.140
Gujarat	Vadodara	0.462
Gujarat	Narmada	0.180
Gujarat	Bharuch	0.847
Gujarat	The Dangs	0.094
Gujarat	Navsari	0.357
Gujarat	Valsad	0.114
Gujarat	Surat	0.550
Gujarat	Tapi	0.129
Daman & Diu	Diu	1.889
Daman & Diu	Daman	0.148
Dadra & Nagar Haveli	Dadra & Nagar Haveli	0.019

Continued…

Table A1: District's proximity from state borders for India, 2011-*Continued*

State name	District name	Border Proximity Factor
Maharashtra	Nandurbar	0.095
Maharashtra	Dhule	0.373
Maharashtra	Jalgaon	0.419
Maharashtra	Buldana	0.602
Maharashtra	Akola	0.616
Maharashtra	Washim	0.984
Maharashtra	Amravati	0.291
Maharashtra	Wardha	0.616
Maharashtra	Nagpur	0.371
Maharashtra	Bhandara	0.404
Maharashtra	Gondiya	0.197
Maharashtra	Gadchiroli	0.162
Maharashtra	Chandrapur	0.484
Maharashtra	Yavatmal	0.433
Maharashtra	Nanded	0.232
Maharashtra	Hingoli	0.835
Maharashtra	Parbhani	1.019
Maharashtra	Jalna	1.276
Maharashtra	Aurangabad	1.264
Maharashtra	Nashik	0.485
Maharashtra	Thane	0.510
Maharashtra	Mumbai Suburban	0.966
Maharashtra	Mumbai	1.139
Maharashtra	Raigarh	1.498
Maharashtra	Pune	1.437
Maharashtra	Ahmadnagar	1.405
Maharashtra	Bid	1.251
Maharashtra	Latur	0.312
Maharashtra	Osmanabad	0.631
Maharashtra	Solapur	0.482
Maharashtra	Satara	1.061
Maharashtra	Ratnagiri	1.128
Maharashtra	Sindhudurg	0.361
Maharashtra	Kolhapur	0.230
Maharashtra	Sangli	0.331
Telangana	Adilabad	0.202
Telangana	Nizamabad	0.369
Telangana	Karimnagar	0.652
Telangana	Medak	0.595
Telangana	Hyderabad	0.788
Telangana	Rangareddy	0.532
Telangana	Mahbubnagar	0.318
Telangana	Nalgonda	0.486
Telangana	Warangal	0.689
Telangana	Khammam	0.178
Andhra Pradesh	Srikakulam	0.274
Andhra Pradesh	Vizianagaram	0.239
Andhra Pradesh	Visakhapatnam	0.346
Andhra Pradesh	East Godavari	0.647
Andhra Pradesh	West Godavari	0.435
Andhra Pradesh	Krishna	0.393
Andhra Pradesh	Guntur	0.399
Andhra Pradesh	Prakasam	0.721

Continued…

Table A1: District's proximity from state borders for India, 2011-*Continued*

State name	District name	Border Proximity Factor
Andhra Pradesh	Sri Potti Sriramulu Nellore	0.906
Andhra Pradesh	Y.S.R.	0.843
Andhra Pradesh	Kurnool	0.351
Andhra Pradesh	Anantapur	0.272
Andhra Pradesh	Chittoor	0.209
Karnataka	Belgaum	0.311
Karnataka	Bagalkot	0.709
Karnataka	Bijapur	0.376
Karnataka	Bidar	0.122
Karnataka	Raichur	0.343
Karnataka	Koppal	0.747
Karnataka	Gadag	1.118
Karnataka	Dharwad	0.826
Karnataka	Uttara Kannada	0.474
Karnataka	Haveri	1.093
Karnataka	Bellary	0.347
Karnataka	Chitradurga	0.383
Karnataka	Davanagere	0.867
Karnataka	Shimoga	1.109
Karnataka	Udupi	0.687
Karnataka	Chikmagalur	0.850
Karnataka	Tumkur	0.353
Karnataka	Bangalore	0.232
Karnataka	Mandya	0.681
Karnataka	Hassan	0.782
Karnataka	Dakshina Kannada	0.216
Karnataka	Kodagu	0.208
Karnataka	Mysore	0.367
Karnataka	Chamarajanagar	0.130
Karnataka	Gulbarga	0.261
Karnataka	Yadgir	0.400
Karnataka	Kolar	0.124
Karnataka	Chikkaballapura	0.157
Karnataka	Bangalore Rural	0.367
Karnataka	Ramanagara	0.284
Goa	North Goa	0.099
Goa	South Goa	0.132
Lakshadweep	Lakshadweep	2.963
Kerala	Kasaragod	0.116
Kerala	Kannur	0.174
Kerala	Wayanad	0.110
Kerala	Kozhikode	0.364
Kerala	Malappuram	0.300
Kerala	Palakkad	0.176
Kerala	Thrissur	0.482
Kerala	Ernakulam	0.461
Kerala	Idukki	0.148
Kerala	Kottayam	0.521
Kerala	Alappuzha	0.720
Kerala	Pathanamthitta	0.317
Kerala	Kollam	0.294
Kerala	Thiruvananthapuram	0.176
Tamil Nadu	Thiruvallur	0.124
Tamil Nadu	Chennai	0.409

Continued…

Table A1: District's proximity from state borders for India, 2011-*Continued*

State name	District name	Border Proximity Factor
Tamil Nadu	Kancheepuram	0.573
Tamil Nadu	Vellore	0.170
Tamil Nadu	Tiruvannamalai	0.555
Tamil Nadu	Viluppuram	0.995
Tamil Nadu	Salem	0.631
Tamil Nadu	Namakkal	0.809
Tamil Nadu	Erode	0.288
Tamil Nadu	The Nilgiris	0.082
Tamil Nadu	Dindigul	0.586
Tamil Nadu	Karur	1.032
Tamil Nadu	Tiruchirappalli	1.304
Tamil Nadu	Perambalur	1.384
Tamil Nadu	Ariyalur	1.670
Tamil Nadu	Cuddalore	1.462
Tamil Nadu	Nagapattinam	2.176
Tamil Nadu	Thiruvarur	2.191
Tamil Nadu	Thanjavur	1.933
Tamil Nadu	Pudukkottai	1.614
Tamil Nadu	Sivaganga	1.271
Tamil Nadu	Madurai	0.715
Tamil Nadu	Theni	0.176
Tamil Nadu	Virudhunagar	0.553
Tamil Nadu	Ramanathapuram	1.286
Tamil Nadu	Thoothukkudi	0.721
Tamil Nadu	Tirunelveli	0.289
Tamil Nadu	Kanniyakumari	0.185
Tamil Nadu	Dharmapuri	0.403
Tamil Nadu	Krishnagiri	0.158
Tamil Nadu	Coimbatore	0.161
Tamil Nadu	Tiruppur	0.455
Puducherry	Yanam	1.135
Puducherry	Puducherry	1.247
Puducherry	Mahe	0.390
Puducherry	Karaikal	2.162
Andaman & Nicobar Islands	Nicobars	14.563
Andaman & Nicobar Islands	North & Middle Andaman	9.866
Andaman & Nicobar Islands	South Andaman	10.912

Table A2: Direct-survey based estimates and model-based estimates of physical,emotional and sexual violence in districts of India, 2015-16.

State name	District name	Physical violence		Emotional violence		Sexual violence	
		Direct-survey based estimates	Model-based estimates	Direct-survey based estimates	Model-based estimates	Direct-survey based estimates	Model-based estimates
		Estimates (Lower bound, Upper bound)	Estimates (Lower bound, Upper bound)	Estimates (Lower bound, Upper bound)	Estimates (Lower bound, Upper bound)	Estimates (Lower bound, Upper bound)	Estimates (Lower bound, Upper bound)
Jammu & Kashmir	Kupwara	11.4(7.2,17.6)	7.1(6.5,7.7)	19.7(9.4,36.7)	15.7(14.8,16.6)	5.0(2.1,11.0)	2.9(2.5,3.3)
Jammu & Kashmir	Badgam	3.8(1.5,9.2)	8.0(7.3,8.7)	6.4(3.0,13.2)	12.7(11.9,13.6)	0.5(0.1,3.6)	3.3(2.8,3.7)
Jammu & Kashmir	Leh(Ladakh)	2.1(0.8,5.3)	4.2(3.0,5.4)	5.7(3.2,9.7)	7.3(5.8,8.8)	0.0(0.0,0.0)	1.2(0.6,1.9)
Jammu & Kashmir	Kargil	6.5(2.7,14.5)	7.3(5.8,8.7)	6.4(2.4,16.3)	7.2(5.8,8.7)	0.8(0.2,2.9)	2.5(1.6,3.3)
Jammu & Kashmir	Punch	4.8(2.2,9.9)	4.6(4.0,5.1)	4.2(1.6,10.7)	6.5(5.8,7.1)	1.1(0.3,5.0)	1.2(0.9,1.5)
Jammu & Kashmir	Rajouri	7.4(3.8,13.9)	5.9(5.3,6.4)	9.3(5.5,15.3)	7.3(6.6,7.9)	8.0(4.1,15.1)	1.7(1.4,2.0)
Jammu & Kashmir	Kathua	6.9(3.4,13.7)	6.1(5.6,6.7)	3.3(1.2,8.4)	7.0(6.4,7.6)	1.5(0.3,7.1)	1.1(0.9,1.4)
Jammu & Kashmir	Baramula	2.3(1.1,5.0)	5.6(5.2,6.1)	1.5(0.5,4.2)	6.3(5.8,6.8)	0.4(0.1,2.9)	1.5(1.2,1.7)
Jammu & Kashmir	Bandipore	12.7(7.1,21.7)	7.0(6.1,7.8)	24.3(14.7,37.4)	10.6(9.6,11.7)	7.0(3.0,15.4)	3.6(3.0,4.3)
Jammu & Kashmir	Srinagar	5.0(2.0,11.8)	4.7(4.4,5.1)	6.5(3.0,13.5)	5.7(5.3,6.1)	0.9(0.1,6.3)	1.1(0.9,1.3)
Jammu & Kashmir	Ganderbal	4.4(2.3,8.4)	5.4(4.5,6.2)	7.9(3.9,15.2)	8.7(7.6,9.7)	1.2(0.3,5.0)	2.7(2.1,3.3)
Jammu & Kashmir	Pulwama	2.1(0.5,7.7)	7.7(7.0,8.4)	9.0(3.6,21.0)	10.6(9.8,11.4)	0.6(0.1,3.9)	3.0(2.5,3.5)
Jammu & Kashmir	Shupiyan	7.0(3.2,14.6)	5.2(4.3,6.0)	6.2(2.5,14.7)	6.5(5.6,7.4)	1.2(0.2,8.5)	1.5(1.1,2.0)
Jammu & Kashmir	Anantnag	3.1(1.2,7.6)	7.1(6.6,7.6)	12.7(6.9,22.3)	10.2(9.6,10.8)	2.8(1.0,7.9)	2.9(2.6,3.3)
Jammu & Kashmir	Kulgam	4.5(1.9,10.4)	5.4(4.7,6.1)	7.0(3.7,13.0)	7.5(6.7,8.3)	0.5(0.1,3.7)	2.8(2.3,3.3)
Jammu & Kashmir	Doda	9.2(4.4,18.4)	7.8(7.0,8.6)	10.5(5.4,19.3)	7.1(6.4,7.9)	0.5(0.1,3.7)	1.9(1.5,2.3)
Jammu & Kashmir	Ramban	3.3(1.3,8.2)	7.3(6.3,8.2)	4.7(2.3,9.7)	6.2(5.3,7.1)	0.5(0.1,3.8)	1.1(0.8,1.5)
Jammu & Kashmir	Kishtwar	5.8(3.1,10.7)	5.1(4.2,6.0)	6.7(3.2,13.2)	7.7(6.6,8.7)	2.3(0.7,6.6)	1.7(1.2,2.2)
Jammu & Kashmir	Udhampur	5.6(2.6,11.4)	5.3(4.7,5.8)	4.8(2.0,10.9)	6.4(5.8,7.0)	0.6(0.1,4.0)	1.8(1.4,2.1)
Jammu & Kashmir	Reasi	10.6(5.4,19.8)	6.6(5.8,7.5)	8.5(4.1,16.6)	7.8(6.9,8.7)	2.9(0.9,9.3)	1.7(1.3,2.2)
Jammu & Kashmir	Jammu	5.1(2.5,10.2)	4.0(3.7,4.3)	3.9(1.8,8.1)	5.3(5.0,5.6)	1.6(0.4,6.7)	0.6(0.5,0.7)
Jammu & Kashmir	Samba	9.9(5.6,16.9)	6.7(5.9,7.4)	8.4(4.3,15.9)	7.7(6.9,8.6)	3.4(1.2,9.4)	2.2(1.8,2.7)
Himanchal Pradesh	Lahaul & Spiti	2.7(0.7,9.3)	1.6(1.3,2.0)	2.4(0.6,9.6)	3.6(3.1,4.1)	1.4(0.2,9.8)	2.1(1.7,2.4)
Himanchal Pradesh	Bilaspur	2.8(1.0,7.5)	2.4(2.0,2.7)	1.4(0.3,5.7)	3.0(2.8,3.3)	0.9(0.1,6.3)	1.4(1.3,1.6)
Himanchal Pradesh	Hamirpur	1.1(0.2,7.2)	2.4(0.7,4.0)	1.1(0.2,7.2)	2.0(0.5,3.5)	0.0(0.0,0.0)	0.7(-0.2,1.7)
Himanchal Pradesh	Kinnaur	2.4(0.6,9.6)	2.5(2.1,2.9)	5.2(2.6,10.2)	5.0(4.4,5.6)	3.8(1.5,9.1)	2.0(1.6,2.4)
Himanchal Pradesh	Chamba	0.4(0.1,3.1)	2.6(2.4,2.8)	5.3(2.0,13.7)	4.7(4.3,5.1)	0.6(0.1,4.4)	2.1(1.8,2.3)
Himanchal Pradesh	Shimla	2.3(0.9,5.9)	2.8(2.4,3.2)	2.3(0.9,5.7)	2.6(2.2,3.0)	2.7(0.8,8.9)	1.7(1.4,2.1)
Himanchal Pradesh	Solan	2.3(0.7,6.8)	3.2(2.1,4.4)	4.8(2.0,11.1)	2.3(2.0,2.7)	1.5(0.4,5.4)	1.7(1.4,2.0)
Himanchal Pradesh	Una	2.7(1.0,6.9)	3.2(2.8,3.7)	2.5(0.7,8.9)	2.5(2.1,3.0)	2.1(0.5,9.1)	0.9(0.6,1.2)
Himanchal Pradesh	Sirmaur	1.8(0.6,5.5)	3.3(2.9,3.6)	7.4(3.1,16.4)	3.8(3.3,4.2)	3.6(0.8,14.9)	2.3(1.9,2.6)
Himanchal Pradesh	Mandi	3.9(1.3,11.2)	3.4(3.1,3.7)	2.3(0.9,5.9)	2.4(2.0,2.8)	0.5(0.1,3.5)	1.2(0.9,1.5)
Himanchal Pradesh	Kangra	6.2(2.1,17.0)	3.6(3.2,4.0)	0.5(0.1,3.7)	3.1(2.8,3.5)	0.5(0.1,3.5)	1.9(1.7,2.2)
Himanchal Pradesh	Kullu	5.9(3.4,10.1)	3.9(3.3,4.4)	3.6(1.3,10.0)	4.0(2.8,5.3)	1.3(0.3,5.0)	1.4(0.6,2.1)
Punjab	Gurdaspur	15.2(8.2,26.5)	16.2(15.8,16.7)	6.0(2.0,17.0)	6.6(6.3,6.9)	9.3(3.2,23.9)	3.9(3.6,4.1)
Punjab	Kapurthala	16.0(9.6,25.3)	14.7(14.0,15.5)	4.2(0.8,20.1)	5.3(4.8,5.7)	0.0(0.0,0.0)	4.3(3.9,4.7)
Punjab	Jalandhar	17.8(8.9,32.3)	17.2(16.7,17.6)	6.8(3.1,14.4)	6.8(6.5,7.1)	2.6(0.5,11.8)	1.6(1.4,1.7)
Punjab	Hoshiarpur	24.6(12.6,42.3)	13.1(12.6,13.5)	13.0(6.5,24.1)	5.7(5.3,6.0)	5.8(1.8,16.9)	1.8(1.6,2.0)
Punjab	Sangrur	14.3(5.4,32.8)	21.2(20.6,21.7)	9.7(4.9,18.4)	7.9(7.5,8.3)	9.1(4.2,18.5)	5.6(5.2,5.9)
Punjab	Fatehgarh Sahib	11.8(5.2,24.4)	13.7(12.9,14.5)	4.5(1.2,15.7)	6.0(5.4,6.5)	0.4(0.1,3.0)	1.7(1.4,2.0)
Punjab	Ludhiana	9.4(2.6,29.1)	14.3(13.9,14.6)	3.2(1.0,10.0)	5.3(5.0,5.5)	8.3(1.1,42.1)	4.0(3.8,4.2)
Punjab	Moga	23.7(12.0,41.4)	17.1(16.4,17.8)	4.5(1.6,12.0)	8.3(7.8,8.8)	3.4(0.9,12.6)	3.8(3.5,4.2)
Punjab	Firozpur	15.6(9.3,24.8)	16.0(15.5,16.4)	12.6(5.7,25.7)	5.7(5.5,6.0)	6.8(2.9,15.0)	3.7(3.4,3.9)
Punjab	Muktsar	11.2(5.0,23.4)	11.9(11.3,12.5)	4.0(1.1,13.7)	6.6(6.1,7.1)	2.6(0.7,9.6)	2.3(2.0,2.6)
Punjab	Faridkot	13.7(8.2,21.9)	22.0(21.1,23.0)	4.2(1.2,13.8)	5.9(5.4,6.4)	0.0(0.0,0.0)	3.0(2.6,3.3)
Punjab	Bathinda	14.4(9.3,21.7)	18.2(17.6,18.7)	5.7(2.4,12.8)	7.5(7.1,7.9)	2.0(0.5,8.2)	5.0(4.7,5.3)
Punjab	Mansa	21.3(14.8,29.5)	23.4(22.6,24.3)	11.4(6.9,18.4)	8.2(7.6,8.8)	5.4(2.5,11.3)	3.8(3.4,4.1)
Punjab	Patiala	16.5(9.2,28.0)	13.9(13.5,14.3)	7.7(2.2,23.1)	6.0(5.7,6.3)	0.8(0.1,5.6)	1.8(1.6,2.0)
Punjab	Amritsar	15.2(6.2,32.5)	17.4(17.0,17.9)	7.9(3.4,17.4)	8.0(7.7,8.3)	4.0(1.5,10.1)	6.2(5.9,6.4)
Punjab	Taran Taran	16.7(6.3,37.5)	17.6(16.9,18.2)	2.4(0.8,6.5)	6.4(6.0,6.8)	2.0(0.3,12.4)	4.8(4.4,5.2)
Punjab	Rupnagar	4.3(1.5,12.1)	13.4(12.6,14.1)	3.3(0.7,14.2)	5.8(5.3,6.3)	0.0(0.0,0.0)	1.9(1.6,2.2)
Punjab	Sahibzada Ajit Singh Nagar	6.8(3.8,11.9)	9.6(9.1,10.1)	3.7(1.6,8.1)	7.9(7.5,8.4)	3.7(1.6,8.1)	7.4(7.0,7.9)
Punjab	Shahid Bhagat Singh Nagar	21.1(13.5,31.4)	13.1(12.3,13.9)	11.2(8.0,15.6)	5.7(5.2,6.3)	3.9(1.3,10.8)	2.4(2.0,2.8)
Punjab	Barnala	35.9(21.8,53.0)	21.7(20.7,22.6)	5.5(3.4,8.8)	6.0(5.4,6.5)	1.7(0.3,8.6)	3.0(2.6,3.3)
Chandigarh	Chandigarh	12.1(5.9,23.2)	12.1(11.5,12.7)	3.5(1.4,8.6)	3.5(3.2,3.8)	3.4(0.9,11.4)	3.4(3.1,3.7)
Uttarakhand	Uttarkashi	3.8(1.7,8.2)	7.6(6.8,8.5)	0.7(0.1,5.3)	4.9(4.2,5.6)	0.0(0.0,0.0)	2.5(2.0,3.0)
Uttarakhand	Chamoli	9.7(3.7,22.9)	5.4(4.7,6.0)	4.5(1.7,11.4)	3.9(3.4,4.5)	2.4(0.5,9.5)	2.2(1.8,2.6)
Uttarakhand	Rudraprayag	4.6(1.9,10.7)	6.1(5.2,6.9)	3.4(1.6,7.0)	3.9(3.2,4.6)	3.3(1.1,9.7)	3.0(2.4,3.6)
Uttarakhand	Tehri Garhwal	4.8(1.7,13.0)	4.9(4.4,5.4)	3.8(1.3,10.3)	3.1(2.7,3.5)	4.8(1.7,13.0)	1.3(1.1,1.6)

Continued…

Table A2: Direct-survey based estimates and model-based estimates of physical,emotional and sexual violence in districts of India, 2015-16.

State name	District name	Physical violence		Emotional violence		Sexual violence	
		Direct-survey based estimates	Model-based estimates	Direct-survey based estimates	Model-based estimates	Direct-survey based estimates	Model-based estimates
		Estimates (Lower bound, Upper bound)	Estimates (Lower bound, Upper bound)	Estimates (Lower bound, Upper bound)	Estimates (Lower bound, Upper bound)	Estimates (Lower bound, Upper bound)	Estimates (Lower bound, Upper bound)
Uttarakhand	Dehradun	6.2(3.5,10.7)	8.1(7.7,8.5)	1.5(0.4,5.3)	3.5(3.3,3.8)	0.9(0.1,5.7)	1.6(1.4,1.7)
Uttarakhand	Garhwal	6.9(2.7,16.7)	7.3(6.7,7.9)	3.3(1.2,8.7)	4.1(3.6,4.5)	0.9(0.1,6.8)	2.9(2.5,3.2)
Uttarakhand	Pithoragarh	3.2(1.1,9.1)	7.2(6.5,7.8)	0.8(0.1,5.4)	5.2(4.6,5.7)	1.5(0.5,4.6)	4.3(3.8,4.8)
Uttarakhand	Bageshwar	2.6(1.3,5.1)	6.4(5.5,7.2)	1.3(0.2,8.0)	2.8(2.2,3.3)	1.3(0.2,8.0)	1.9(1.4,2.4)
Uttarakhand	Almora	7.4(4.1,13.0)	5.3(4.8,5.8)	4.2(1.2,13.2)	2.5(2.2,2.9)	0.0(0.0,0.0)	1.1(0.8,1.3)
Uttarakhand	Champawat	8.2(3.5,18.0)	8.0(7.0,9.0)	2.1(0.5,8.8)	3.0(2.4,3.6)	4.2(1.2,13.4)	2.2(1.7,2.8)
Uttarakhand	Nainital	11.0(6.0,19.3)	6.9(6.5,7.4)	16.2(7.5,31.8)	4.4(4.0,4.8)	3.0(1.4,6.3)	1.7(1.5,2.0)
Uttarakhand	Udham Singh Nagar	11.1(7.0,17.0)	9.4(9.0,9.8)	5.7(2.8,11.2)	6.1(5.8,6.5)	4.6(2.3,9.1)	3.7(3.5,4.0)
Uttarakhand	Hardwar	10.2(5.6,18.0)	7.1(6.7,7.4)	3.0(1.4,6.2)	3.3(3.1,3.6)	3.4(1.3,8.5)	2.1(1.9,2.3)
Haryana	Panchkula	26.6(14.2,44.2)	24.4(23.4,25.4)	10.9(4.3,24.9)	9.4(8.7,10.1)	6.4(2.4,15.9)	5.0(4.5,5.5)
Haryana	Ambala	12.2(5.4,25.2)	19.0(18.3,19.6)	6.6(3.4,12.4)	8.5(8.0,8.9)	2.5(1.1,5.6)	3.7(3.4,4.0)
Haryana	Yamunanagar	21.6(15.5,29.3)	23.4(22.7,24.1)	15.4(6.8,31.2)	11.4(10.9,11.9)	9.0(2.4,28.9)	6.3(5.9,6.7)
Haryana	Kurukshetra	36.3(30.0,43.1)	27.0(26.2,27.8)	12.6(6.2,24.1)	9.5(9.0,10.1)	12.9(5.6,27.1)	7.6(7.2,8.1)
Haryana	Kaithal	28.9(20.2,39.5)	29.4(28.6,30.2)	12.7(8.8,17.8)	16.5(15.8,17.1)	4.9(4.2,5.6)	13.0(12.4,13.6)
Haryana	Karnal	33.9(20.2,50.9)	26.9(26.2,27.5)	12.5(5.1,27.6)	12.4(11.9,12.8)	10.0(1.7,41.6)	9.9(9.4,10.3)
Haryana	Panipat	11.8(4.6,27.2)	23.4(22.7,24.1)	4.0(1.2,12.5)	11.7(11.2,12.2)	0.8(0.1,5.6)	6.5(6.1,6.9)
Haryana	Sonipat	11.3(7.8,16.2)	16.0(15.4,16.5)	0.6(0.1,3.8)	9.8(9.4,10.3)	0.0(0.0,0.0)	4.3(4.0,4.6)
Haryana	Jind	26.0(18.4,35.5)	24.1(23.4,24.7)	13.0(4.6,31.9)	13.9(13.4,14.4)	5.7(1.8,16.3)	5.9(5.5,6.2)
Haryana	Fatehabad	20.7(14.9,28.1)	25.9(25.1,26.7)	12.3(3.8,33.7)	10.4(9.8,11.0)	7.1(4.0,12.3)	7.6(7.1,8.1)
Haryana	Sirsa	27.9(17.2,41.7)	26.5(25.8,27.1)	13.0(8.8,18.8)	12.2(11.7,12.7)	14.3(8.5,22.9)	7.9(7.5,8.3)
Haryana	Hisar	35.7(22.8,51.0)	26.5(25.9,27.1)	13.9(6.1,28.6)	12.0(11.6,12.5)	7.5(5.0,11.1)	8.3(8.0,8.7)
Haryana	Bhiwani	28.1(17.1,42.7)	27.3(26.7,28.0)	14.8(6.1,31.7)	12.2(11.7,12.6)	11.7(3.6,32.0)	10.4(10.0,10.9)
Haryana	Rohtak	24.3(14.3,38.3)	21.9(21.2,22.6)	8.4(6.2,11.4)	9.7(9.1,10.2)	4.3(1.7,10.8)	7.3(6.8,7.7)
Haryana	Jhajjar	29.5(19.8,41.5)	23.3(22.5,24.0)	11.0(4.8,23.4)	11.5(11.0,12.1)	5.1(0.9,24.1)	6.5(6.0,6.9)
Haryana	Mahendragarh	25.5(20.3,31.5)	29.5(28.7,30.3)	19.5(13.0,28.3)	12.0(11.4,12.6)	12.4(7.9,18.8)	7.8(7.4,8.3)
Haryana	Rewari	24.5(13.4,40.6)	21.6(20.8,22.3)	11.6(4.7,25.6)	11.1(10.5,11.6)	7.2(2.7,17.8)	5.9(5.5,6.3)
Haryana	Gurgaon	23.0(9.6,45.6)	23.3(22.7,23.9)	4.1(1.5,10.5)	8.9(8.5,9.3)	0.0(0.0,0.0)	5.1(4.7,5.4)
Haryana	Mewat	25.9(17.3,36.9)	27.7(26.9,28.6)	16.5(7.7,32.1)	12.5(11.9,13.1)	15.4(9.2,24.7)	10.8(10.2,11.4)
Haryana	Faridabad	17.4(8.9,31.3)	23.8(23.2,24.3)	12.6(5.9,25.0)	10.6(10.2,11.0)	4.8(1.3,15.9)	3.3(3.1,3.5)
Haryana	Palwal	28.9(18.4,42.3)	29.3(28.5,30.2)	12.2(5.5,25.0)	12.2(11.6,12.8)	9.2(4.0,19.6)	8.1(7.6,8.6)
NCT Of Delhi	North West	15.7(7.6,29.5)	17.2(16.8,17.5)	17.1(9.6,28.5)	11.2(10.9,11.5)	2.8(0.7,10.2)	6.1(5.8,6.3)
NCT Of Delhi	North District	21.0(9.4,40.5)	16.1(15.4,16.8)	6.3(1.6,21.8)	10.5(9.9,11.1)	9.1(3.6,21.1)	6.0(5.6,6.5)
NCT Of Delhi	North East	29.8(15.9,48.8)	30.1(29.6,30.7)	13.2(6.0,26.4)	8.7(8.3,9.0)	7.5(2.0,24.6)	3.0(2.8,3.2)
NCT Of Delhi	East	29.2(17.3,45.0)	22.6(22.0,23.1)	8.2(2.9,21.3)	8.6(8.3,9.0)	11.3(3.8,29.0)	5.0(4.7,5.3)
NCT Of Delhi	New Delhi	11.9(2.7,39.4)	21.4(19.4,23.3)	2.2(0.3,12.8)	7.8(6.5,9.1)	0.0(0.0,0.0)	3.9(3.0,4.8)
NCT Of Delhi	Central	26.5(17.5,38.2)	20.0(19.0,20.9)	8.4(2.5,24.3)	7.5(6.8,8.1)	7.0(1.5,26.4)	3.9(3.4,4.4)
NCT Of Delhi	West	21.1(11.8,34.9)	22.3(21.8,22.7)	8.1(2.2,25.6)	8.6(8.3,8.9)	4.6(0.7,24.1)	4.8(4.6,5.1)
NCT Of Delhi	South West	21.2(11.0,37.0)	23.9(23.4,24.4)	14.7(7.7,26.4)	9.2(8.9,9.5)	0.0(0.0,0.0)	5.3(5.0,5.5)
NCT Of Delhi	South	12.9(4.3,32.9)	15.9(15.5,16.3)	4.3(1.5,11.9)	10.5(10.1,10.8)	1.5(0.2,10.1)	5.9(5.6,6.1)
Rajasthan	Ganganagar	36.3(26.4,47.4)	18.2(17.7,18.7)	6.6(2.1,18.5)	6.7(6.4,7.0)	2.6(0.6,11.7)	2.0(1.8,2.2)
Rajasthan	Hanumangarh	15.2(8.9,24.7)	16.0(15.5,16.5)	3.6(1.2,10.1)	4.8(4.5,5.1)	3.6(0.9,12.8)	1.4(1.2,1.5)
Rajasthan	Bikaner	14.6(8.7,23.6)	21.4(20.9,21.9)	4.7(2.5,8.8)	8.0(7.6,8.3)	2.6(0.9,7.2)	3.1(2.9,3.3)
Rajasthan	Churu	15.8(11.4,21.6)	18.8(18.3,19.3)	3.9(1.5,9.4)	6.8(6.5,7.1)	3.8(1.7,8.4)	2.4(2.2,2.6)
Rajasthan	Jhunjhunun	32.5(23.0,43.8)	17.9(17.4,18.3)	12.1(5.8,23.5)	5.2(4.9,5.4)	2.7(0.7,10.4)	1.7(1.5,1.9)
Rajasthan	Alwar	16.8(12.4,22.3)	15.6(15.3,16.0)	4.1(2.1,8.1)	4.4(4.3,4.6)	1.4(0.5,3.4)	1.5(1.4,1.6)
Rajasthan	Bharatpur	20.0(15.2,25.8)	19.0(18.5,19.4)	12.9(8.0,20.0)	8.2(7.9,8.5)	4.3(1.5,11.4)	3.8(3.6,4.0)
Rajasthan	Dhaulpur	24.7(15.9,36.2)	22.6(21.9,23.3)	5.1(2.4,10.6)	7.2(6.7,7.6)	6.8(2.3,18.4)	4.1(3.8,4.5)
Rajasthan	Karauli	15.1(9.5,23.1)	20.6(20.0,21.2)	5.0(1.9,12.4)	5.1(4.8,5.5)	3.4(1.2,9.3)	1.9(1.7,2.1)
Rajasthan	Sawai Madhopur	27.1(17.4,39.7)	21.3(20.7,21.9)	10.8(4.9,22.0)	5.3(5.0,5.7)	2.7(0.9,8.0)	2.7(2.5,2.9)
Rajasthan	Dausa	13.6(7.4,23.7)	21.2(20.6,21.7)	2.5(0.6,10.4)	6.1(5.8,6.4)	0.0(0.0,0.0)	2.9(2.7,3.1)
Rajasthan	Jaipur	14.7(9.3,22.6)	15.2(14.9,15.4)	3.7(1.6,8.2)	5.9(5.7,6.1)	3.4(1.6,7.0)	2.5(2.4,2.6)
Rajasthan	Sikar	10.9(5.9,19.1)	14.7(14.4,15.1)	3.4(1.2,9.2)	6.6(6.3,6.9)	3.0(1.1,7.8)	1.7(1.6,1.9)
Rajasthan	Nagaur	15.2(9.3,23.9)	17.2(16.8,17.5)	8.0(2.4,23.6)	5.6(5.3,5.8)	0.0(0.0,0.0)	2.1(1.9,2.2)
Rajasthan	Jodhpur	20.3(11.2,34.0)	16.6(16.3,17.0)	10.4(4.8,21.0)	8.4(8.1,8.6)	1.9(0.3,12.3)	2.4(2.3,2.5)
Rajasthan	Jaisalmer	10.7(4.0,25.5)	15.9(15.1,16.7)	3.1(0.9,9.9)	5.6(5.1,6.1)	5.7(2.5,12.5)	4.3(3.8,4.7)
Rajasthan	Barmer	11.4(5.8,21.2)	19.1(18.6,19.5)	4.5(1.6,12.5)	8.8(8.5,9.1)	0.8(0.1,5.6)	1.8(1.7,2.0)
Rajasthan	Jalor	19.7(13.3,28.1)	18.7(18.2,19.2)	10.9(4.7,23.4)	11.5(11.0,11.9)	0.8(0.1,5.2)	2.6(2.4,2.8)
Rajasthan	Sirohi	11.2(6.2,19.4)	17.0(16.3,17.7)	1.6(0.4,6.2)	4.7(4.3,5.1)	0.0(0.0,0.0)	3.7(3.4,4.1)
Rajasthan	Pali	22.9(13.3,36.4)	19.0(18.5,19.5)	17.0(8.0,32.5)	6.6(6.3,6.9)	2.3(0.3,15.3)	2.6(2.4,2.8)

Continued…

Table A2: Direct-survey based estimates and model-based estimates of physical,emotional and sexual violence in districts of India, 2015-16.

State name	District name	Physical violence		Emotional violence		Sexual violence	
		Direct-survey based estimates	Model-based estimates	Direct-survey based estimates	Model-based estimates	Direct-survey based estimates	Model-based estimates
		Estimates (Lower bound, Upper bound)	Estimates (Lower bound, Upper bound)	Estimates (Lower bound, Upper bound)	Estimates (Lower bound, Upper bound)	Estimates (Lower bound, Upper bound)	Estimates (Lower bound, Upper bound)
Rajasthan	Ajmer	8.8(4.6,16.1)	15.2(14.8,15.5)	3.2(1.2,8.2)	4.4(4.2,4.6)	2.3(0.8,6.6)	2.3(2.1,2.4)
Rajasthan	Tonk	7.8(2.4,22.3)	15.8(15.2,16.3)	0.8(0.1,6.2)	6.5(6.1,6.8)	1.7(0.4,7.0)	2.2(2.0,2.4)
Rajasthan	Bundi	12.0(7.6,18.3)	18.3(17.7,19.0)	5.1(2.0,12.3)	9.5(9.1,10.0)	0.0(0.0,0.0)	2.3(2.0,2.5)
Rajasthan	Bhilwara	14.3(5.4,32.8)	19.4(19.0,19.8)	1.9(0.8,4.7)	5.8(5.6,6.1)	1.0(0.1,6.8)	3.2(3.0,3.4)
Rajasthan	Rajsamand	25.4(12.2,45.5)	15.6(15.0,16.2)	7.5(3.2,16.8)	5.1(4.7,5.4)	1.6(0.2,10.9)	1.6(1.4,1.8)
Rajasthan	Dungarpur	21.8(11.4,37.7)	21.1(20.5,21.7)	8.4(3.1,20.8)	4.8(4.5,5.1)	3.9(1.2,12.2)	2.3(2.0,2.5)
Rajasthan	Banswara	17.8(8.6,33.4)	21.0(20.5,21.6)	7.9(3.9,15.5)	6.3(6.0,6.6)	0.8(0.1,5.3)	2.5(2.3,2.8)
Rajasthan	Chittaurgarh	8.0(3.8,16.1)	18.4(17.9,18.9)	0.9(0.1,6.0)	6.1(5.8,6.4)	3.2(0.7,13.1)	3.0(2.7,3.2)
Rajasthan	Kota	21.9(14.0,32.7)	16.9(16.4,17.4)	7.7(4.0,14.4)	6.7(6.4,7.0)	6.1(2.6,13.7)	3.4(3.2,3.7)
Rajasthan	Baran	25.3(12.1,45.6)	19.0(18.4,19.6)	15.4(8.1,27.4)	7.3(6.9,7.7)	2.2(0.5,9.1)	1.9(1.7,2.1)
Rajasthan	Jhalawar	13.6(8.5,21.0)	18.5(17.9,19.0)	1.7(0.2,12.4)	7.0(6.6,7.3)	0.0(0.0,0.0)	1.5(1.3,1.7)
Rajasthan	Udaipur	28.4(21.6,36.2)	21.4(21.0,21.8)	10.2(6.7,15.3)	5.7(5.4,5.9)	6.6(2.7,15.4)	2.2(2.1,2.4)
Rajasthan	Pratapgarh	33.9(22.4,47.6)	21.3(20.5,22.0)	6.2(3.5,10.5)	4.7(4.3,5.1)	0.0(0.0,0.0)	1.7(1.5,2.0)
Uttar Pradesh	Saharanpur	20.5(14.8,27.6)	21.0(20.6,21.4)	13.5(8.1,21.5)	11.0(10.7,11.4)	4.5(2.2,8.9)	4.4(4.2,4.6)
Uttar Pradesh	Muzaffarpur	19.2(8.7,37.2)	18.4(18.0,18.7)	12.7(5.0,28.6)	8.9(8.7,9.2)	6.0(1.7,19.5)	5.6(5.4,5.8)
Uttar Pradesh	Bijnor	17.0(6.5,37.5)	23.3(22.9,23.8)	5.7(1.5,19.1)	10.0(9.7,10.3)	5.0(2.0,12.1)	4.3(4.1,4.5)
Uttar Pradesh	Moradabad	18.2(12.6,25.7)	23.2(22.8,23.6)	10.8(6.0,18.8)	10.1(9.8,10.4)	4.8(2.6,8.8)	6.5(6.3,6.7)
Uttar Pradesh	Rampur	26.1(18.1,36.1)	24.8(24.2,25.3)	8.9(5.0,15.3)	11.6(11.2,12.0)	5.6(1.3,20.5)	5.8(5.5,6.1)
Uttar Pradesh	Jyotiba Phule Nagar	15.0(6.4,31.1)	24.4(23.8,25.0)	10.3(2.2,36.8)	11.3(10.9,11.8)	1.3(0.2,8.8)	6.4(6.1,6.8)
Uttar Pradesh	Meerut	24.0(15.9,34.6)	22.2(21.8,22.6)	11.2(7.7,16.0)	10.0(9.7,10.3)	7.5(4.7,11.8)	5.4(5.2,5.6)
Uttar Pradesh	Baghpat	14.4(8.3,23.9)	20.8(20.2,21.5)	6.3(2.5,15.1)	7.7(7.3,8.2)	1.3(0.2,8.7)	2.6(2.4,2.9)
Uttar Pradesh	Ghaziabad	16.7(11.2,24.1)	21.5(21.1,21.8)	7.0(3.8,12.5)	11.6(11.3,11.8)	4.1(1.5,10.9)	6.5(6.3,6.7)
Uttar Pradesh	Gautam Buddha Nagar	23.3(15.0,34.4)	18.0(17.5,18.6)	7.6(3.7,15.1)	7.5(7.1,7.8)	0.8(0.2,3.7)	4.5(4.3,4.8)
Uttar Pradesh	Bulandshahr	21.5(14.4,31.0)	22.2(21.7,22.6)	6.0(2.1,15.9)	10.4(10.1,10.8)	2.6(1.0,6.5)	3.6(3.4,3.8)
Uttar Pradesh	Aligarh	19.8(13.1,28.7)	26.3(25.8,26.7)	9.7(4.9,18.2)	11.5(11.2,11.8)	3.7(1.9,6.9)	8.0(7.7,8.3)
Uttar Pradesh	Mahamaya Nagar	15.8(8.2,28.0)	25.6(24.9,26.3)	11.3(4.2,27.0)	11.0(10.5,11.4)	4.4(2.0,9.3)	4.3(4.0,4.6)
Uttar Pradesh	Mathura	8.6(4.2,16.7)	20.8(20.3,21.3)	8.4(2.6,23.7)	8.9(8.6,9.3)	2.4(0.6,8.6)	3.0(2.8,3.2)
Uttar Pradesh	Agra	21.5(14.3,31.1)	25.5(25.1,25.9)	6.8(3.6,12.5)	10.9(10.6,11.2)	4.7(2.5,8.5)	5.7(5.5,5.9)
Uttar Pradesh	Firozabad	22.5(16.7,29.5)	22.6(22.1,23.1)	10.0(5.6,17.3)	8.4(8.1,8.7)	7.9(3.5,17.0)	7.3(7.0,7.6)
Uttar Pradesh	Mainpuri	21.3(12.1,34.9)	25.8(25.2,26.4)	10.9(6.1,18.5)	11.6(11.1,12.0)	10.2(5.0,19.8)	6.1(5.7,6.4)
Uttar Pradesh	Budaun	16.9(10.6,25.7)	25.6(25.2,26.1)	3.5(1.7,7.1)	11.0(10.7,11.3)	3.4(1.2,9.1)	5.9(5.6,6.1)
Uttar Pradesh	Bareilly	34.1(24.9,44.7)	25.4(25.0,25.8)	9.9(5.5,17.4)	13.0(12.7,13.3)	5.5(2.9,10.2)	8.1(7.9,8.4)
Uttar Pradesh	Pilibhit	25.4(15.5,38.8)	24.4(23.8,25.0)	14.1(8.6,22.4)	8.6(8.3,9.0)	7.4(2.3,21.1)	5.3(5.0,5.6)
Uttar Pradesh	Shahjahanpur	21.2(13.0,32.5)	20.4(19.9,20.8)	4.3(2.1,8.8)	7.8(7.5,8.1)	4.1(1.7,9.9)	4.7(4.5,5.0)
Uttar Pradesh	Kheri	28.7(19.9,39.5)	30.1(29.6,30.5)	12.2(6.6,21.2)	10.1(9.8,10.3)	0.0(0.0,0.0)	3.8(3.6,4.0)
Uttar Pradesh	Sitapur	24.9(17.7,33.9)	27.9(27.5,28.3)	10.5(6.8,15.9)	14.1(13.8,14.4)	2.4(0.6,9.7)	7.0(6.7,7.2)
Uttar Pradesh	Hardoi	43.0(34.3,52.2)	28.3(27.8,28.7)	6.7(3.1,13.9)	9.1(8.8,9.4)	3.4(1.7,6.7)	4.6(4.4,4.8)
Uttar Pradesh	Unnao	27.4(19.1,37.6)	22.5(22.0,22.9)	5.5(2.0,14.5)	9.6(9.2,9.9)	2.3(0.3,14.3)	4.9(4.6,5.1)
Uttar Pradesh	Lucknow	9.3(4.8,17.2)	18.2(17.9,18.5)	8.2(3.8,16.6)	10.2(10.0,10.5)	1.9(0.8,4.3)	4.1(3.9,4.2)
Uttar Pradesh	Rae Bareli	15.6(8.0,28.3)	21.1(20.7,21.6)	5.0(1.7,14.1)	11.4(11.1,11.7)	0.0(0.0,0.0)	3.6(3.4,3.7)
Uttar Pradesh	Farrukhabad	29.4(19.4,41.9)	26.9(26.2,27.5)	9.9(4.3,21.2)	10.5(10.0,10.9)	6.5(3.5,11.9)	5.3(5.0,5.6)
Uttar Pradesh	Kannauj	18.4(10.2,30.8)	21.8(21.2,22.4)	10.5(6.4,16.7)	11.1(10.7,11.6)	5.1(2.7,9.5)	3.6(3.3,3.9)
Uttar Pradesh	Etawah	11.5(7.7,16.9)	20.9(20.3,21.5)	4.4(1.4,12.7)	7.8(7.4,8.2)	5.1(1.9,13.1)	5.0(4.7,5.3)
Uttar Pradesh	Auraiya	43.7(28.9,59.7)	28.4(27.7,29.2)	14.8(7.7,26.6)	8.9(8.4,9.3)	5.9(2.5,13.3)	4.3(3.9,4.6)
Uttar Pradesh	Kanpur Dehat	23.2(15.5,33.3)	26.0(25.4,26.7)	8.2(2.7,22.6)	8.2(7.9,8.6)	1.8(0.4,7.0)	2.2(2.0,2.4)
Uttar Pradesh	Kanpur Nagar	21.1(11.4,35.8)	22.7(22.4,23.1)	8.8(2.6,25.8)	10.1(9.8,10.3)	4.1(1.7,9.3)	3.8(3.6,4.0)
Uttar Pradesh	Jalaun	23.9(17.8,31.4)	24.5(23.9,25.1)	10.3(4.9,20.4)	8.4(8.0,8.8)	1.2(0.1,8.7)	5.1(4.8,5.4)
Uttar Pradesh	Jhansi	24.7(15.4,37.1)	26.3(25.8,26.8)	4.3(2.2,8.4)	9.9(9.6,10.3)	1.1(0.3,4.3)	4.8(4.5,5.1)
Uttar Pradesh	Lalitpur	21.8(12.7,35.0)	29.6(28.9,30.4)	3.7(1.1,11.7)	9.2(8.7,9.7)	0.8(0.1,5.7)	4.0(3.7,4.4)
Uttar Pradesh	Hamirpur	31.5(20.5,45.1)	26.9(26.1,27.7)	8.0(3.0,19.6)	8.8(8.3,9.3)	4.3(1.1,15.5)	4.0(3.6,4.3)
Uttar Pradesh	Mahoba	13.0(7.3,22.0)	29.4(28.5,30.3)	0.0(0.0,0.0)	8.9(8.3,9.4)	0.0(0.0,0.0)	5.9(5.5,6.4)
Uttar Pradesh	Banda	33.7(21.2,49.0)	31.0(30.3,31.6)	15.3(9.5,23.7)	11.7(11.3,12.2)	9.9(4.4,20.6)	6.0(5.6,6.3)
Uttar Pradesh	Chitrakoot	17.4(9.6,29.4)	23.3(22.5,24.1)	12.1(5.0,26.3)	10.1(9.6,10.7)	6.8(2.0,20.4)	4.5(4.1,4.9)
Uttar Pradesh	Fatehpur	42.0(31.0,53.9)	25.3(24.8,25.8)	11.3(5.1,23.2)	11.2(10.8,11.5)	9.2(4.1,19.1)	6.4(6.1,6.6)
Uttar Pradesh	Pratapgarh	33.7(21.9,48.0)	22.2(21.8,22.6)	5.4(1.7,15.5)	11.2(10.8,11.5)	1.5(0.4,4.9)	6.2(6.0,6.5)
Uttar Pradesh	Kaushambi	30.1(19.4,43.6)	30.1(29.4,30.8)	8.0(3.9,15.5)	10.3(9.9,10.8)	4.1(1.5,10.7)	4.1(3.8,4.4)
Uttar Pradesh	Allahabad	25.6(14.5,41.1)	22.7(22.3,23.0)	11.9(4.6,27.2)	14.2(14.0,14.5)	7.6(3.0,17.8)	6.8(6.6,7.0)
Uttar Pradesh	Bara Banki	27.4(16.4,41.9)	27.1(26.6,27.5)	12.0(7.3,19.0)	12.6(12.2,12.9)	7.9(4.5,13.6)	7.9(7.6,8.2)
Uttar Pradesh	Faizabad	30.2(20.5,42.0)	28.5(27.9,29.0)	15.6(6.7,32.3)	10.2(9.8,10.5)	9.6(3.9,21.7)	5.3(5.0,5.5)

Continued…

Table A2: Direct-survey based estimates and model-based estimates of physical,emotional and sexual violence in districts of India, 2015-16.

State name	District name	Physical violence: Direct-survey based estimates	Physical violence: Model-based estimates	Emotional violence: Direct-survey based estimates	Emotional violence: Model-based estimates	Sexual violence: Direct-survey based estimates	Sexual violence: Model-based estimates
		Estimates (Lower bound, Upper bound)	Estimates (Lower bound, Upper bound)	Estimates (Lower bound, Upper bound)	Estimates (Lower bound, Upper bound)	Estimates (Lower bound, Upper bound)	Estimates (Lower bound, Upper bound)
Uttar Pradesh	Ambedkar Nagar	18.3(9.1,33.5)	26.3(25.8,26.9)	11.8(6.1,21.7)	11.6(11.2,11.9)	6.9(3.2,14.1)	8.3(7.9,8.6)
Uttar Pradesh	Sultanpur	26.6(15.0,42.7)	27.8(27.4,28.2)	7.1(2.3,20.0)	9.3(9.1,9.6)	3.8(1.3,10.8)	7.0(6.8,7.3)
Uttar Pradesh	Bahraich	52.6(36.7,68.1)	35.8(35.3,36.3)	23.1(15.7,32.7)	14.0(13.7,14.3)	19.7(10.7,33.3)	12.1(11.8,12.5)
Uttar Pradesh	Shrawasti	37.2(23.5,53.3)	29.3(28.5,30.1)	16.8(9.6,27.8)	13.6(13.0,14.2)	10.6(3.9,25.6)	7.8(7.3,8.2)
Uttar Pradesh	Balrampur	37.6(23.2,54.7)	29.3(28.7,29.9)	21.1(14.1,30.4)	18.3(17.8,18.8)	10.4(4.6,22.2)	6.9(6.6,7.2)
Uttar Pradesh	Gonda	23.4(13.0,38.5)	30.0(29.5,30.4)	15.9(6.9,32.5)	14.9(14.6,15.3)	6.3(2.5,15.1)	7.6(7.3,7.8)
Uttar Pradesh	Siddharth nagar	39.9(28.1,52.9)	29.7(29.2,30.2)	16.1(8.5,28.5)	10.4(10.0,10.8)	19.1(11.2,30.6)	7.7(7.4,8.1)
Uttar Pradesh	Basti	22.6(13.0,36.3)	30.7(30.2,31.3)	5.8(2.3,14.0)	11.4(11.0,11.7)	5.7(1.4,20.7)	6.1(5.9,6.4)
Uttar Pradesh	Sant Kabir Nagar	26.4(18.1,36.9)	28.0(27.3,28.6)	16.5(9.6,26.8)	9.8(9.4,10.2)	4.2(1.0,16.0)	5.4(5.1,5.7)
Uttar Pradesh	Mahrajganj	32.4(19.9,48.0)	29.2(28.7,29.7)	17.8(8.7,32.8)	13.5(13.1,13.9)	5.5(1.7,16.0)	6.3(6.1,6.6)
Uttar Pradesh	Gorakhpur	40.1(24.8,57.5)	26.9(26.5,27.3)	21.8(12.0,36.1)	13.3(13.0,13.6)	17.1(9.9,27.9)	9.2(9.0,9.5)
Uttar Pradesh	Kushinagar	38.3(24.3,54.6)	29.5(29.0,29.9)	19.9(11.3,32.6)	13.8(13.4,14.1)	12.4(4.4,30.4)	6.9(6.6,7.1)
Uttar Pradesh	Deoria	18.5(12.2,26.9)	19.8(19.4,20.2)	5.7(1.6,18.6)	8.2(8.0,8.5)	4.1(1.8,8.9)	5.9(5.7,6.2)
Uttar Pradesh	Azamgarh	38.7(26.8,52.0)	28.6(28.2,29.0)	24.1(14.1,37.9)	14.0(13.7,14.3)	14.4(8.1,24.2)	10.5(10.3,10.8)
Uttar Pradesh	Mau	24.5(11.0,45.9)	21.5(21.0,22.0)	16.2(8.3,29.3)	12.3(11.9,12.8)	2.4(1.1,5.4)	3.4(3.2,3.6)
Uttar Pradesh	Ballia	16.8(10.0,26.8)	25.2(24.7,25.6)	6.6(2.5,16.5)	9.1(8.8,9.4)	3.6(1.1,11.0)	5.9(5.7,6.1)
Uttar Pradesh	Jaunpur	17.6(10.1,28.8)	21.9(21.5,22.3)	7.4(3.2,16.4)	8.6(8.3,8.8)	1.1(0.2,7.7)	6.0(5.8,6.2)
Uttar Pradesh	Ghazipur	22.5(13.3,35.6)	27.1(26.7,27.5)	3.3(0.8,12.9)	9.1(8.8,9.3)	4.2(1.9,8.9)	4.4(4.2,4.6)
Uttar Pradesh	Chandauli	38.3(27.9,49.8)	24.2(23.6,24.7)	26.3(18.2,36.5)	11.8(11.4,12.2)	5.2(1.7,15.0)	3.4(3.1,3.6)
Uttar Pradesh	Varanasi	24.9(17.0,34.8)	28.4(27.9,28.8)	11.3(6.8,18.4)	12.8(12.5,13.1)	7.9(4.7,13.0)	6.4(6.2,6.7)
Uttar Pradesh	Sant Ravidas Nagar (Bhadohi)	26.4(15.8,40.5)	25.1(24.4,25.7)	21.4(12.6,34.0)	18.2(17.6,18.7)	6.9(2.6,17.0)	6.1(5.7,6.4)
Uttar Pradesh	Mirzapur	23.5(13.5,37.6)	28.2(27.7,28.7)	7.9(4.0,14.9)	10.2(9.9,10.6)	8.3(4.7,14.3)	3.7(3.5,3.9)
Uttar Pradesh	Sonbhadra	38.8(22.4,58.1)	30.5(29.9,31.1)	18.5(8.3,36.2)	13.7(13.2,14.1)	7.9(2.4,23.2)	6.7(6.3,7.0)
Uttar Pradesh	Etah	15.3(8.9,25.0)	23.4(22.8,24.1)	8.3(3.7,17.5)	11.7(11.2,12.1)	3.2(1.0,10.0)	3.5(3.3,3.8)
Uttar Pradesh	Kanshiram Nagar	27.4(17.1,40.8)	22.8(22.1,23.5)	10.1(6.1,16.3)	8.6(8.1,9.0)	9.2(5.1,16.2)	5.5(5.1,5.8)
Bihar	Pashchim Champaran	25.4(16.9,36.3)	33.7(33.3,34.1)	21.1(12.9,32.5)	20.6(20.2,21.0)	12.0(5.2,25.1)	13.0(12.7,13.3)
Bihar	Purba Champaran	34.5(20.9,51.2)	42.7(42.3,43.1)	20.8(12.1,33.3)	20.6(20.2,20.9)	23.2(15.7,33.0)	17.5(17.2,17.8)
Bihar	Sheohar	32.4(22.3,44.3)	38.5(37.4,39.6)	10.5(5.0,20.5)	18.6(17.7,19.5)	15.0(6.7,30.4)	11.3(10.5,12.0)
Bihar	Sitamarhi	49.1(38.6,59.7)	37.9(37.4,38.4)	22.5(14.6,33.0)	19.6(19.2,20.0)	12.8(7.3,21.3)	11.2(10.8,11.5)
Bihar	Madhubani	25.0(15.6,37.6)	32.0(31.6,32.4)	20.7(13.5,30.2)	18.8(18.5,19.2)	15.2(6.6,31.4)	11.1(10.8,11.4)
Bihar	Supaul	25.8(17.1,36.9)	37.8(37.2,38.4)	10.3(3.4,27.4)	16.3(15.8,16.7)	10.0(4.2,21.9)	10.2(9.8,10.5)
Bihar	Araria	46.3(31.7,61.5)	36.2(35.7,36.7)	22.2(13.2,34.9)	15.6(15.2,16.0)	10.4(4.6,21.8)	9.0(8.6,9.3)
Bihar	Kishanganj	36.9(24.1,51.9)	30.5(29.8,31.1)	18.4(10.2,30.7)	15.2(14.7,15.7)	12.6(5.9,24.9)	8.2(7.8,8.6)
Bihar	Purnia	39.5(23.5,58.1)	36.9(36.4,37.4)	14.8(7.4,27.3)	15.6(15.2,16.0)	12.1(6.1,22.4)	8.9(8.6,9.2)
Bihar	Katihar	32.9(20.5,48.4)	33.8(33.3,34.3)	21.5(10.9,37.9)	18.7(18.3,19.2)	5.3(2.5,11.1)	13.7(13.3,14.0)
Bihar	Madhepura	55.3(37.8,71.6)	42.3(41.7,43.0)	28.4(17.3,42.9)	17.8(17.3,18.3)	10.5(4.9,21.1)	13.1(12.7,13.5)
Bihar	Saharsa	34.5(24.6,45.9)	37.9(37.3,38.6)	21.4(10.8,38.0)	21.1(20.5,21.6)	11.1(6.1,19.2)	18.1(17.6,18.6)
Bihar	Darbhanga	28.1(15.0,46.3)	38.9(38.4,39.3)	8.1(3.1,19.8)	17.8(17.5,18.2)	11.0(5.2,21.7)	17.4(17.1,17.8)
Bihar	Muzaffarnagar	32.2(22.4,43.9)	33.7(33.3,34.1)	19.0(10.9,31.2)	20.3(20.0,20.6)	15.2(7.3,29.1)	10.2(10.0,10.5)
Bihar	Gopalganj	29.7(20.8,40.4)	32.1(31.6,32.7)	13.7(6.2,27.8)	18.5(18.0,18.9)	11.8(6.3,21.2)	10.5(10.2,10.9)
Bihar	Siwan	26.4(16.3,39.9)	23.5(23.0,23.9)	10.2(6.6,15.4)	15.1(14.7,15.4)	3.4(1.2,9.3)	6.3(6.1,6.6)
Bihar	Saran	36.3(21.9,53.7)	36.3(35.8,36.7)	13.7(9.3,19.8)	18.2(17.8,18.6)	24.5(14.2,38.8)	13.5(13.1,13.8)
Bihar	Vaishali	39.5(27.6,52.8)	42.2(41.8,42.7)	18.4(9.7,32.3)	18.0(17.6,18.4)	11.4(4.0,28.4)	8.0(7.8,8.3)
Bihar	Samastipur	25.8(17.1,37.0)	33.3(32.9,33.8)	19.8(13.8,27.7)	19.3(19.0,19.7)	9.3(3.9,20.4)	13.3(13.0,13.6)
Bihar	Begusarai	33.6(22.7,46.6)	39.9(39.4,40.4)	20.9(8.6,42.5)	17.0(16.6,17.4)	5.0(1.1,19.0)	8.3(8.0,8.6)
Bihar	Khagaria	34.6(21.4,50.7)	40.3(39.6,41.0)	19.2(9.9,33.8)	19.8(19.3,20.4)	8.3(4.7,14.1)	12.0(11.6,12.5)
Bihar	Bhagalpur	37.3(25.7,50.4)	38.9(38.4,39.4)	17.9(10.7,28.5)	19.5(19.1,20.0)	9.3(4.3,19.3)	14.0(13.6,14.4)
Bihar	Banka	35.2(26.3,45.2)	33.9(33.3,34.5)	19.8(11.3,32.4)	19.1(18.6,19.6)	12.3(6.5,22.1)	12.5(12.0,12.9)
Bihar	Munger	40.0(27.6,53.9)	27.5(26.8,28.2)	21.2(9.0,42.1)	16.7(16.1,17.3)	7.1(3.3,14.5)	7.0(6.6,7.4)
Bihar	Lakhisarai	42.8(34.0,52.2)	39.9(39.0,40.8)	26.8(21.4,33.0)	18.2(17.5,18.9)	17.0(9.0,29.8)	12.7(12.1,13.3)
Bihar	Sheikhpura	47.6(33.5,62.2)	41.2(40.1,42.3)	13.0(6.9,23.1)	18.9(18.0,19.8)	13.6(5.9,28.2)	11.8(11.1,12.6)
Bihar	Nalanda	36.5(22.9,52.8)	38.1(37.6,38.6)	14.6(9.2,22.3)	15.7(15.3,16.1)	4.4(1.7,11.4)	6.8(6.5,7.0)
Bihar	Patna	45.6(33.7,58.0)	37.1(36.7,37.4)	20.5(12.9,30.9)	19.2(18.9,19.5)	10.6(5.5,19.4)	10.1(9.9,10.4)
Bihar	Bhojpur	31.7(20.3,45.9)	32.2(31.6,32.7)	21.0(12.8,32.6)	15.6(15.2,16.0)	11.3(5.7,21.3)	9.5(9.2,9.9)
Bihar	Buxar	39.1(32.3,46.3)	32.4(31.7,33.1)	25.6(16.1,38.1)	16.0(15.5,16.5)	18.5(10.4,30.8)	9.7(9.3,10.1)
Bihar	Kaimur (Bhabua)	40.4(25.5,57.3)	41.4(40.7,42.1)	15.8(8.0,29.0)	16.3(15.8,16.8)	2.1(0.7,5.8)	7.5(7.1,7.8)
Bihar	Rohtas	28.4(18.5,40.9)	26.5(26.0,26.9)	16.0(6.9,32.7)	12.4(12.1,12.8)	4.0(1.7,9.1)	8.0(7.7,8.3)
Bihar	Aurangabad	33.0(21.1,47.7)	38.3(37.8,38.9)	12.9(6.7,23.6)	23.7(23.3,24.2)	1.2(0.3,5.2)	10.0(9.6,10.3)
Bihar	Gaya	41.5(33.4,50.2)	35.7(35.2,36.1)	20.3(10.7,35.1)	20.7(20.3,21.0)	7.3(3.2,15.6)	9.9(9.6,10.1)

Continued…

Table A2: Direct-survey based estimates and model-based estimates of physical,emotional and sexual violence in districts of India, 2015-16.

State name	District name	Physical violence: Direct-survey based estimates	Physical violence: Model-based estimates	Emotional violence: Direct-survey based estimates	Emotional violence: Model-based estimates	Sexual violence: Direct-survey based estimates	Sexual violence: Model-based estimates
		Estimates (Lower bound, Upper bound)	Estimates (Lower bound, Upper bound)	Estimates (Lower bound, Upper bound)	Estimates (Lower bound, Upper bound)	Estimates (Lower bound, Upper bound)	Estimates (Lower bound, Upper bound)
Bihar	Nawada	38.6(30.7,47.2)	35.8(35.3,36.4)	13.8(6.8,25.9)	17.7(17.3,18.2)	4.2(0.8,18.8)	9.3(9.0,9.7)
Bihar	Jamui	29.4(18.5,43.4)	28.4(27.8,29.0)	12.1(6.6,21.1)	13.0(12.6,13.5)	11.9(5.4,24.2)	9.1(8.7,9.5)
Bihar	Jehanabad	28.2(21.8,35.7)	37.3(36.4,38.1)	13.5(6.0,27.8)	14.3(13.8,14.9)	14.6(9.0,22.9)	9.0(8.6,9.5)
Bihar	Arwal	37.3(25.0,51.5)	31.6(30.6,32.6)	14.6(6.8,28.4)	15.2(14.4,15.9)	10.2(3.7,25.1)	8.4(7.8,9.0)
Sikkim	North	1.1(0.1,7.5)	1.8(0.5,3.0)	3.2(1.1,8.7)	2.5(1.0,3.9)	1.3(0.4,4.3)	1.5(0.3,2.6)
Sikkim	West District	1.2(0.3,4.8)	2.0(1.3,2.7)	1.7(0.4,7.4)	2.3(1.5,3.0)	1.2(0.3,4.8)	1.4(0.8,2.0)
Sikkim	South District	1.6(0.1,16.1)	1.8(1.1,2.4)	0.0(0.0,0.0)	2.1(1.4,2.8)	0.0(0.0,0.0)	0.8(0.4,1.3)
Sikkim	East District	2.2(0.7,7.1)	1.1(0.7,1.4)	3.6(1.3,9.1)	2.1(1.6,2.6)	1.3(0.2,8.6)	0.8(0.5,1.1)
Arunachal Pradesh	Tawang	11.6(4.2,28.5)	11.3(8.2,14.3)	8.1(5.7,11.5)	6.7(4.3,9.1)	2.5(0.3,16.3)	2.0(0.7,3.3)
Arunachal Pradesh	West Kameng	18.1(7.8,36.6)	19.7(17.2,22.3)	8.3(3.8,17.4)	12.3(10.2,14.4)	0.0(0.0,0.0)	3.5(2.4,4.7)
Arunachal Pradesh	East Kameng	19.2(12.1,29.2)	15.3(13.0,17.7)	16.6(12.6,21.6)	12.7(10.5,14.9)	7.1(2.3,19.8)	4.7(3.3,6.1)
Arunachal Pradesh	Papum Pare	30.7(19.5,44.8)	17.5(15.8,19.1)	12.9(6.4,24.3)	13.3(11.8,14.7)	13.9(5.5,30.6)	6.9(5.8,8.0)
Arunachal Pradesh	Upper Subansiri	41.2(30.4,52.8)	24.8(22.1,27.6)	20.5(11.2,34.4)	15.4(13.0,17.7)	18.5(11.8,27.8)	9.4(7.5,11.3)
Arunachal Pradesh	West Siang	17.4(8.8,31.3)	19.7(17.5,21.9)	15.5(6.9,31.4)	15.6(13.6,17.7)	2.9(0.4,18.4)	8.0(6.4,9.5)
Arunachal Pradesh	East Siang	28.9(19.2,41.1)	21.7(19.3,24.1)	33.1(15.1,58.0)	14.0(11.9,16.0)	7.3(2.5,19.3)	6.5(5.0,7.9)
Arunachal Pradesh	Upper Siang	17.4(6.9,37.5)	18.3(14.3,22.3)	10.1(2.3,35.0)	13.7(10.1,17.3)	4.5(1.1,16.5)	6.0(3.5,8.5)
Arunachal Pradesh	Changlang	14.8(7.1,28.1)	22.4(20.4,24.4)	9.9(5.7,16.7)	14.6(12.8,16.3)	2.8(0.7,10.4)	7.1(5.8,8.3)
Arunachal Pradesh	Tirap	20.4(8.7,40.9)	24.9(22.3,27.4)	17.2(6.8,37.3)	18.6(16.3,20.9)	7.7(2.9,18.8)	10.4(8.6,12.2)
Arunachal Pradesh	Lower Subansiri	18.5(11.2,29.0)	23.1(20.3,25.9)	6.8(2.5,17.1)	14.3(12.0,16.6)	6.3(1.8,19.9)	7.0(5.3,8.7)
Arunachal Pradesh	Kurung Kumey	13.8(8.8,21.1)	24.1(21.5,26.7)	9.0(3.4,21.7)	19.2(16.7,21.6)	8.0(3.5,17.1)	11.7(9.7,13.6)
Arunachal Pradesh	Dibang Valley	5.8(1.6,18.5)	16.0(7.9,24.1)	15.7(6.0,35.1)	14.7(6.9,22.5)	0.0(0.0,0.0)	3.1(-0.7,6.9)
Arunachal Pradesh	Lower Dibang Valley	16.0(8.7,27.4)	21.4(18.1,24.6)	7.5(4.0,13.5)	11.9(9.4,14.5)	0.9(0.1,5.6)	4.3(2.7,5.9)
Arunachal Pradesh	Lohit	13.5(8.1,21.8)	17.0(15.2,18.8)	9.1(3.6,21.2)	13.3(11.7,14.9)	3.1(1.2,7.5)	3.1(2.3,3.9)
Arunachal Pradesh	Anjaw	29.5(21.4,39.0)	20.7(15.2,26.2)	19.4(12.9,28.0)	11.3(7.0,15.6)	9.8(4.9,18.8)	4.5(1.7,7.4)
Nagaland	Mon	1.0(0.1,6.6)	4.9(4.0,5.8)	10.7(3.5,28.4)	6.5(5.5,7.5)	5.1(0.7,28.6)	3.0(2.3,3.7)
Nagaland	Mokokchung	1.7(0.3,9.6)	4.1(3.2,5.0)	3.9(1.1,13.2)	5.7(4.6,6.8)	1.7(0.3,9.6)	2.7(1.9,3.4)
Nagaland	Zunheboto	3.2(0.8,11.6)	3.8(2.7,4.8)	5.0(1.7,13.6)	7.7(6.2,9.2)	0.0(0.0,0.0)	1.3(0.7,1.9)
Nagaland	Wokha	2.1(0.4,10.3)	6.5(5.2,7.7)	4.5(1.2,15.4)	6.7(5.5,8.0)	1.0(0.1,7.6)	3.1(2.3,4.0)
Nagaland	Dimapur	17.6(11.6,25.7)	4.6(4.0,5.3)	11.2(6.1,19.4)	5.6(4.9,6.3)	8.7(4.4,16.6)	3.1(2.5,3.6)
Nagaland	Phek	6.5(2.8,14.0)	5.4(4.3,6.6)	8.3(2.9,21.3)	5.7(4.5,6.9)	5.9(2.0,16.2)	4.1(3.1,5.1)
Nagaland	Tuensang	1.2(0.2,8.9)	5.4(4.3,6.4)	3.6(1.9,6.8)	5.7(4.6,6.8)	4.7(1.5,13.6)	4.1(3.1,5.0)
Nagaland	Longleng	3.8(1.0,12.9)	6.4(4.2,8.5)	1.8(0.4,7.9)	6.9(4.6,9.1)	3.4(1.0,11.3)	4.0(2.2,5.7)
Nagaland	Kiphire	10.3(5.0,20.0)	8.4(6.4,10.5)	9.0(3.9,19.4)	8.4(6.4,10.4)	3.6(0.9,13.2)	8.8(6.7,10.8)
Nagaland	Kohima	2.1(0.5,8.4)	4.6(3.8,5.5)	6.0(2.4,14.0)	7.2(6.1,8.2)	1.0(0.3,3.1)	3.1(2.4,3.8)
Nagaland	Peren	9.5(6.1,14.3)	5.3(3.8,6.7)	11.0(5.3,21.4)	9.4(7.5,11.3)	3.3(0.9,10.7)	2.1(1.1,3.0)
Manipur	Senapati	36.9(24.2,51.8)	39.6(38.1,41.1)	13.2(5.3,29.4)	12.6(11.6,13.6)	13.2(5.4,28.9)	7.4(6.6,8.2)
Manipur	Tamenglong	39.5(27.6,52.7)	40.7(38.0,43.3)	12.4(7.4,20.2)	12.7(10.9,14.6)	5.7(2.4,12.6)	7.8(6.4,9.3)
Manipur	Churachandpur	40.0(27.2,54.4)	36.4(34.6,38.2)	3.3(1.1,9.8)	10.9(9.7,12.0)	5.4(2.4,11.7)	7.9(6.9,8.9)
Manipur	Bishnupur	31.9(24.3,40.6)	28.4(26.6,30.1)	16.5(11.0,23.9)	12.1(10.8,13.3)	10.0(5.2,18.3)	7.0(6.0,8.0)
Manipur	Thoubal	24.0(17.0,32.7)	24.2(22.9,25.4)	11.5(6.5,19.6)	9.1(8.3,9.9)	8.7(4.3,17.0)	9.8(8.9,10.6)
Manipur	Imphal West	22.3(16.1,30.1)	23.4(22.3,24.5)	7.8(3.5,16.5)	10.5(9.7,11.3)	10.9(5.6,20.2)	6.1(5.5,6.7)
Manipur	Imphal East	38.0(28.1,49.0)	31.1(29.8,32.4)	10.3(5.0,19.9)	12.1(11.2,13.0)	4.4(2.0,9.4)	7.7(7.0,8.5)
Manipur	Ukhrul	25.4(12.3,45.1)	30.4(28.1,32.6)	12.2(5.6,24.6)	9.0(7.6,10.4)	3.3(1.1,10.0)	7.8(6.5,9.2)
Manipur	Chandel	26.6(13.0,46.6)	30.5(28.2,32.9)	10.4(3.1,29.5)	9.0(7.5,10.4)	3.2(1.1,9.0)	3.5(2.6,4.5)
Mizoram	Mamit	21.5(14.8,30.0)	15.7(13.3,18.1)	7.6(3.8,14.6)	10.0(8.1,12.0)	3.3(0.9,10.9)	2.8(1.7,3.9)
Mizoram	Kolasib	7.9(3.1,18.9)	11.3(9.1,13.5)	8.9(4.3,17.4)	7.9(6.1,9.8)	3.2(1.3,7.6)	1.8(0.9,2.7)
Mizoram	Aizawl	9.5(4.4,19.3)	11.7(10.7,12.7)	9.1(4.5,17.7)	8.7(7.8,9.6)	1.1(0.2,6.5)	2.8(2.3,3.3)
Mizoram	Champhai	12.7(7.2,21.3)	10.2(8.5,11.8)	9.5(5.1,17.0)	6.8(5.4,8.2)	3.8(1.4,10.1)	2.4(1.6,3.3)
Mizoram	Serchhip	14.2(8.3,23.2)	10.9(8.4,13.4)	10.5(5.1,20.1)	6.1(4.2,8.1)	1.2(0.3,4.6)	1.5(0.5,2.4)
Mizoram	Lunglei	11.4(7.2,17.5)	12.1(10.5,13.7)	7.4(4.3,12.4)	9.3(7.9,10.7)	1.6(0.4,6.4)	2.1(1.4,2.8)
Mizoram	Lawngtlai	13.5(6.9,24.8)	16.2(14.2,18.2)	9.8(3.1,26.9)	10.2(8.5,11.9)	2.9(0.5,15.3)	3.5(2.5,4.5)
Mizoram	Saiha	10.5(6.5,16.5)	13.4(10.6,16.2)	5.6(3.0,10.4)	9.9(7.4,12.3)	1.1(0.1,7.5)	2.4(1.1,3.6)
Tripura	West Tripura	17.3(10.7,26.7)	20.9(20.4,21.4)	12.7(8.2,19.2)	10.2(9.8,10.6)	8.9(4.1,18.2)	7.1(6.8,7.5)
Tripura	South Tripura	19.9(13.3,28.7)	19.5(18.7,20.2)	10.8(6.2,18.2)	11.0(10.4,11.5)	8.8(3.9,18.7)	8.6(8.1,9.1)
Tripura	Dhalai	26.6(17.8,37.9)	28.9(27.6,30.2)	10.8(5.1,21.7)	12.3(11.4,13.3)	8.2(3.1,20.0)	7.5(6.8,8.3)
Tripura	North Tripura	24.3(15.9,35.3)	18.9(18.0,19.7)	9.7(5.4,16.9)	10.6(10.0,11.3)	6.3(2.6,14.2)	8.9(8.3,9.5)
Meghalaya	West Garo Hills	29.4(19.6,41.6)	22.9(21.9,23.9)	5.7(2.4,12.8)	7.4(6.8,8.0)	1.5(0.3,7.5)	1.6(1.3,1.9)
Meghalaya	East Garo Hills	40.9(26.5,57.0)	25.4(23.9,26.8)	15.9(10.3,23.6)	13.7(12.5,14.8)	10.2(5.1,19.2)	4.3(3.7,5.0)
Meghalaya	South Garo Hills	26.3(15.1,41.7)	27.3(25.0,29.6)	14.6(9.9,20.9)	15.0(13.2,16.9)	0.0(0.0,0.0)	4.5(3.4,5.5)

Continued…

Table A2: Direct-survey based estimates and model-based estimates of physical,emotional and sexual violence in districts of India, 2015-16.

State name	District name	Physical violence: Direct-survey based estimates	Physical violence: Model-based estimates	Emotional violence: Direct-survey based estimates	Emotional violence: Model-based estimates	Sexual violence: Direct-survey based estimates	Sexual violence: Model-based estimates
		Estimates (Lower bound, Upper bound)	Estimates (Lower bound, Upper bound)	Estimates (Lower bound, Upper bound)	Estimates (Lower bound, Upper bound)	Estimates (Lower bound, Upper bound)	Estimates (Lower bound, Upper bound)
Meghalaya	West Khasi Hills	7.2(3.2,15.3)	25.8(24.3,27.2)	3.5(1.8,6.7)	9.8(8.8,10.8)	3.1(0.6,13.7)	3.8(3.1,4.4)
Meghalaya	Ribhoi	18.2(9.8,31.4)	20.9(19.4,22.5)	12.8(6.9,22.5)	10.4(9.2,11.6)	2.7(1.0,7.5)	3.7(3.0,4.5)
Meghalaya	East Khasi Hills	10.9(6.2,18.6)	12.0(11.2,12.7)	6.9(4.2,11.1)	6.1(5.5,6.6)	0.8(0.2,3.2)	1.4(1.1,1.7)
Meghalaya	Jaintia Hills	18.6(9.0,34.6)	17.4(16.1,18.6)	16.3(7.7,31.3)	13.3(12.2,14.4)	3.3(0.7,13.6)	2.4(1.9,2.9)
Assam	Kokrajhar	25.9(14.0,42.7)	16.5(15.9,17.2)	16.1(6.9,33.1)	8.3(7.8,8.8)	7.2(1.2,32.6)	3.8(3.5,4.2)
Assam	Dhubri	14.2(7.1,26.3)	17.7(17.2,18.2)	7.5(3.3,16.2)	7.0(6.6,7.3)	4.7(1.9,11.3)	3.5(3.3,3.7)
Assam	Goalpara	14.5(5.2,34.1)	15.8(15.2,16.5)	7.6(2.4,21.4)	9.5(9.0,10.0)	9.0(2.3,29.2)	4.5(4.1,4.9)
Assam	Barpeta	25.9(12.1,46.9)	20.7(20.1,21.2)	14.0(6.7,26.8)	9.1(8.7,9.5)	7.5(2.9,18.0)	7.9(7.6,8.3)
Assam	Morigaon	26.1(16.5,38.8)	22.8(22.1,23.6)	26.3(17.1,38.3)	16.0(15.3,16.6)	6.1(2.7,13.3)	7.3(6.8,7.8)
Assam	Nagaon	16.2(6.6,34.4)	12.8(12.4,13.1)	9.7(3.7,23.4)	7.4(7.1,7.7)	2.2(0.5,8.8)	3.1(2.9,3.3)
Assam	Sonitpur	16.8(8.4,30.7)	19.1(18.6,19.6)	0.8(0.1,5.1)	8.0(7.7,8.4)	1.6(0.4,6.4)	2.8(2.6,3.0)
Assam	Lakhimpur	10.3(5.2,19.5)	19.5(18.8,20.2)	2.3(0.7,7.8)	9.3(8.8,9.8)	2.2(0.5,9.6)	5.2(4.8,5.6)
Assam	Dhemaji	23.4(13.4,37.7)	25.8(24.9,26.8)	10.6(4.4,23.6)	10.8(10.1,11.5)	7.4(3.0,17.0)	8.3(7.7,8.9)
Assam	Tinsukia	12.6(6.3,23.5)	15.3(14.8,15.9)	9.5(4.3,19.7)	10.0(9.5,10.5)	6.8(2.2,19.3)	4.7(4.4,5.0)
Assam	Dibrugarh	25.8(17.4,36.4)	12.8(12.3,13.3)	11.7(6.5,20.2)	7.3(6.9,7.7)	4.2(0.8,18.7)	4.4(4.1,4.7)
Assam	Sivasagar	14.8(8.4,25.0)	14.9(14.3,15.4)	14.7(8.9,23.3)	11.2(10.7,11.8)	6.8(2.9,15.3)	2.8(2.5,3.1)
Assam	Jorhat	12.6(6.8,22.4)	16.5(15.8,17.1)	2.0(0.6,6.2)	7.8(7.4,8.3)	1.9(0.3,12.4)	3.1(2.8,3.4)
Assam	Golaghat	19.1(8.9,36.3)	15.7(15.1,16.3)	5.8(2.0,15.5)	6.6(6.2,7.0)	3.5(0.9,12.4)	1.5(1.3,1.7)
Assam	Karbi Anglong	20.4(14.4,28.1)	20.3(19.6,21.1)	12.9(6.5,24.1)	12.0(11.4,12.6)	4.1(2.0,8.2)	6.1(5.7,6.6)
Assam	Dima Hasao	20.1(13.4,29.0)	14.7(13.3,16.2)	13.6(7.3,23.9)	10.2(8.9,11.4)	3.5(0.7,15.6)	2.7(2.0,3.4)
Assam	Cachar	9.7(5.5,16.6)	12.1(11.7,12.6)	15.2(7.7,27.8)	8.2(7.8,8.6)	2.6(1.0,6.9)	2.5(2.3,2.7)
Assam	Karimganj	13.6(7.9,22.6)	15.7(15.1,16.3)	4.1(1.5,10.5)	7.6(7.1,8.0)	3.3(1.1,9.6)	3.7(3.4,4.0)
Assam	Hailakandi	7.9(2.8,20.5)	15.5(14.7,16.4)	0.9(0.1,6.2)	7.7(7.1,8.4)	0.8(0.1,5.5)	4.1(3.6,4.5)
Assam	Bongaigaon	9.8(4.4,20.7)	16.8(16.1,17.6)	4.2(1.3,12.2)	8.5(8.0,9.1)	3.1(0.6,13.3)	5.4(5.0,5.9)
Assam	Chirang	21.6(8.5,45.0)	17.0(16.0,17.9)	7.0(2.9,16.0)	8.7(7.9,9.4)	3.3(0.8,13.0)	4.0(3.5,4.6)
Assam	Kamrup	8.0(3.4,17.7)	10.7(10.3,11.2)	2.7(0.9,7.3)	5.8(5.5,6.1)	2.4(0.8,6.6)	3.1(2.8,3.3)
Assam	Kamrup Metropolitan	6.4(3.3,12.1)	9.8(9.3,10.2)	2.3(0.6,8.9)	6.3(5.9,6.7)	2.5(0.6,10.0)	2.8(2.5,3.0)
Assam	Nalbari	24.7(12.2,43.6)	17.8(17.0,18.6)	11.4(4.4,26.2)	7.1(6.5,7.6)	2.2(0.8,5.9)	5.0(4.6,5.5)
Assam	Baksa	18.5(8.8,34.9)	17.3(16.6,18.0)	10.3(3.0,29.9)	8.7(8.2,9.2)	8.3(2.6,23.7)	3.7(3.3,4.0)
Assam	Darrang	23.3(13.3,37.6)	21.9(21.1,22.6)	15.9(9.2,26.1)	15.9(15.2,16.5)	7.0(3.7,12.7)	7.7(7.2,8.2)
Assam	Udalguri	16.5(7.1,33.7)	23.0(22.2,23.9)	5.3(2.2,12.2)	9.6(9.1,10.2)	2.9(0.8,9.6)	3.5(3.2,3.9)
West Bengal	Darjiling	20.1(8.4,40.8)	20.2(19.7,20.7)	21.4(9.9,40.5)	9.9(9.5,10.3)	11.9(5.7,23.1)	7.6(7.2,7.9)
West Bengal	Jalpaiguri	28.7(18.2,42.1)	22.0(21.6,22.4)	12.2(6.6,21.3)	10.0(9.7,10.3)	9.4(6.1,14.3)	8.1(7.9,8.3)
West Bengal	Koch Bihar	25.7(17.7,35.8)	22.0(21.5,22.4)	15.4(5.7,35.7)	8.9(8.6,9.2)	3.8(1.6,8.8)	5.0(4.8,5.3)
West Bengal	Uttar Dinajpur	13.8(6.2,27.9)	16.1(15.7,16.5)	7.4(3.2,16.3)	7.9(7.6,8.2)	7.3(2.5,19.4)	7.5(7.3,7.8)
West Bengal	Dakshin Dinajpur	26.3(16.0,40.2)	24.7(24.2,25.3)	18.9(10.3,32.0)	15.0(14.5,15.4)	16.8(6.2,38.2)	8.6(8.2,8.9)
West Bengal	Maldah	25.4(18.8,33.3)	25.0(24.6,25.4)	12.9(6.8,23.0)	17.5(17.2,17.9)	12.4(4.7,28.8)	10.7(10.4,10.9)
West Bengal	Murshidabad	18.8(11.0,30.1)	16.4(16.2,16.6)	11.5(5.9,21.2)	9.1(8.9,9.3)	8.5(3.8,18.1)	5.2(5.1,5.4)
West Bengal	Birbhum	16.9(9.5,28.1)	17.5(17.1,17.8)	6.8(3.7,12.4)	6.3(6.1,6.5)	3.4(1.4,8.0)	5.7(5.5,6.0)
West Bengal	Barddhaman	12.8(8.2,19.5)	15.3(15.1,15.5)	6.0(3.2,11.2)	6.7(6.6,6.9)	0.0(0.0,0.0)	3.9(3.8,4.1)
West Bengal	Nadia	13.4(7.7,22.3)	13.5(13.2,13.7)	5.6(2.8,10.9)	7.8(7.6,8.0)	1.8(0.4,6.7)	3.3(3.1,3.4)
West Bengal	North Twenty Four Parganas	29.3(9.8,61.4)	18.9(18.7,19.1)	11.2(5.6,21.3)	9.0(8.8,9.1)	7.9(2.0,27.0)	5.1(5.0,5.2)
West Bengal	Hugli	7.5(2.8,18.2)	16.3(16.0,16.5)	3.5(1.4,8.7)	6.5(6.3,6.6)	1.9(0.5,7.1)	3.7(3.5,3.8)
West Bengal	Bankura	13.7(6.4,26.7)	16.6(16.3,17.0)	6.6(3.7,11.4)	8.4(8.2,8.7)	3.0(0.8,10.9)	4.7(4.5,4.9)
West Bengal	Puruliya	12.7(7.8,20.0)	26.3(25.9,26.8)	8.3(3.7,17.7)	17.4(17.0,17.8)	5.9(2.7,12.3)	11.2(10.9,11.5)
West Bengal	Haora	12.1(7.7,18.4)	14.8(14.5,15.1)	4.6(2.0,10.2)	8.6(8.3,8.8)	2.3(0.3,15.4)	5.9(5.8,6.1)
West Bengal	Kolkata	4.2(1.4,12.0)	8.6(8.3,8.8)	2.1(0.3,13.8)	5.7(5.5,5.9)	0.9(0.3,2.9)	1.6(1.5,1.7)
West Bengal	South Twenty Four Parganas	27.1(20.6,34.7)	21.4(21.2,21.7)	16.9(10.6,25.9)	12.3(12.1,12.5)	12.6(6.6,22.6)	9.6(9.5,9.8)
West Bengal	Paschim Medinipur	27.3(14.9,44.7)	15.7(15.4,15.9)	7.0(3.6,13.4)	8.1(7.9,8.3)	5.4(2.7,10.7)	3.9(3.8,4.0)
West Bengal	Purba Medinipur	16.2(7.7,30.9)	20.8(20.5,21.1)	9.4(5.2,16.7)	12.7(12.5,12.9)	1.5(0.4,5.9)	5.3(5.1,5.4)
Jharkhand	Garhwa	33.7(23.7,45.4)	27.2(26.4,27.9)	13.2(6.7,24.3)	8.8(8.4,9.3)	7.5(3.0,17.4)	6.2(5.8,6.6)
Jharkhand	Chatra	35.4(26.5,45.6)	24.2(23.5,25.0)	7.6(3.4,16.0)	8.0(7.5,8.5)	12.9(5.4,27.8)	7.3(6.8,7.7)
Jharkhand	Kodarma	12.4(4.7,28.9)	20.1(19.3,20.9)	1.9(0.7,5.5)	7.6(7.1,8.2)	0.6(0.1,4.0)	2.7(2.4,3.0)
Jharkhand	Giridih	27.5(19.8,37.0)	25.3(24.8,25.8)	9.2(4.1,19.4)	7.7(7.4,8.0)	2.5(0.6,10.2)	6.3(6.0,6.5)
Jharkhand	Deoghar	14.5(8.2,24.4)	25.0(24.4,25.6)	5.5(1.9,15.0)	9.4(9.0,9.9)	1.7(0.4,7.2)	4.6(4.3,4.9)
Jharkhand	Godda	26.6(17.1,38.9)	24.3(23.6,25.0)	4.3(1.6,11.0)	7.6(7.1,8.0)	4.4(1.8,10.3)	7.1(6.7,7.5)
Jharkhand	Sahibganj	24.6(18.6,31.9)	26.1(25.4,26.9)	6.3(2.3,16.2)	5.8(5.4,6.2)	5.6(1.7,17.0)	5.1(4.7,5.4)
Jharkhand	Pakur	26.6(15.6,41.5)	24.2(23.4,25.0)	18.9(9.5,34.0)	7.8(7.3,8.3)	11.8(5.1,25.1)	4.8(4.4,5.2)
Jharkhand	Dhanbad	23.2(13.9,36.1)	22.5(22.0,23.0)	5.5(2.7,10.8)	8.6(8.3,8.9)	14.1(6.7,27.3)	8.6(8.3,8.9)

Continued…

Table A2: Direct-survey based estimates and model-based estimates of physical,emotional and sexual violence in districts of India, 2015-16.							
		**Physical violence**		**Emotional violence**		**Sexual violence**	
		**Direct-survey based estimates**	**Model-based estimates**	**Direct-survey based estimates**	**Model-based estimates**	**Direct-survey based estimates**	**Model-based estimates**
**State name**	**District name**	Estimates (Lower bound, Upper bound)	Estimates (Lower bound, Upper bound)	Estimates (Lower bound, Upper bound)	Estimates (Lower bound, Upper bound)	Estimates (Lower bound, Upper bound)	Estimates (Lower bound, Upper bound)
Jharkhand	Bokaro	26.6(19.2,35.6)	24.9(24.4,25.5)	5.3(2.3,11.8)	6.8(6.5,7.1)	2.9(1.3,6.3)	5.5(5.3,5.8)
Jharkhand	Lohardaga	11.9(4.1,30.0)	13.4(12.4,14.3)	3.3(1.0,10.4)	4.1(3.5,4.6)	2.0(0.6,6.8)	4.0(3.4,4.5)
Jharkhand	Purbi Singhbhum	4.2(1.7,9.8)	14.5(14.1,14.9)	2.3(0.8,6.2)	5.1(4.8,5.4)	2.7(0.8,8.2)	2.5(2.3,2.7)
Jharkhand	Palamu	32.1(19.0,48.8)	23.5(22.9,24.0)	16.1(7.7,30.6)	7.8(7.5,8.2)	6.8(3.3,13.5)	7.3(7.0,7.7)
Jharkhand	Latehar	18.7(9.2,34.2)	27.3(26.4,28.3)	9.3(3.9,20.7)	8.6(8.0,9.2)	3.9(1.2,11.5)	6.3(5.8,6.9)
Jharkhand	Hazaribagh	19.3(12.8,27.8)	18.0(17.5,18.5)	7.3(2.9,17.0)	7.1(6.8,7.5)	1.3(0.2,8.6)	2.9(2.6,3.1)
Jharkhand	Ramgarh	32.4(23.0,43.5)	24.3(23.5,25.1)	6.7(3.6,12.4)	6.7(6.3,7.2)	11.9(5.2,24.8)	6.2(5.8,6.7)
Jharkhand	Dumka	20.6(10.7,35.9)	26.0(25.3,26.6)	2.4(0.8,6.9)	5.2(4.9,5.6)	3.1(0.9,10.4)	4.1(3.8,4.4)
Jharkhand	Jamtara	16.7(9.0,28.8)	20.4(19.6,21.3)	4.1(1.4,11.1)	5.6(5.1,6.0)	0.0(0.0,0.0)	3.4(3.0,3.7)
Jharkhand	Ranchi	9.3(5.6,15.1)	16.5(16.1,16.9)	5.2(2.3,11.3)	7.6(7.3,7.9)	2.3(0.7,7.7)	5.5(5.3,5.7)
Jharkhand	Khunti	15.7(9.5,24.8)	20.8(19.8,21.9)	5.5(2.2,12.7)	6.7(6.0,7.3)	4.1(1.0,15.1)	4.4(3.9,4.9)
Jharkhand	Gumla	18.5(10.9,29.6)	23.2(22.4,24.0)	5.8(2.9,11.3)	5.2(4.8,5.6)	4.9(3.3,7.1)	4.5(4.1,4.9)
Jharkhand	Simdega	39.5(29.3,50.8)	20.9(19.9,21.9)	9.9(4.8,19.3)	6.9(6.2,7.5)	6.1(3.1,12.0)	4.3(3.8,4.8)
Jharkhand	Pashchimi Singhbhum	29.1(19.0,41.8)	22.2(21.6,22.9)	8.9(4.2,18.1)	9.5(9.0,9.9)	3.1(0.9,10.0)	5.9(5.5,6.2)
Jharkhand	Saraikela-Kharsawan	14.9(7.9,26.2)	19.4(18.7,20.1)	5.1(2.3,10.9)	5.6(5.2,6.0)	8.0(2.6,22.2)	5.0(4.6,5.4)
Odisha	Bargarh	15.1(7.9,26.9)	18.6(18.0,19.2)	6.3(3.3,11.8)	7.3(6.9,7.7)	2.5(0.5,11.1)	3.1(2.9,3.4)
Odisha	Jharsuguda	20.2(11.3,33.3)	17.1(16.2,18.0)	7.7(3.6,15.8)	6.2(5.6,6.8)	6.6(3.7,11.7)	5.1(4.6,5.6)
Odisha	Sambalpur	21.0(10.6,37.2)	21.9(21.2,22.7)	9.7(4.0,21.4)	8.1(7.6,8.6)	4.7(1.2,16.4)	4.7(4.4,5.1)
Odisha	Debagarh	21.6(14.0,31.6)	18.8(17.6,20.1)	8.6(4.4,16.2)	6.9(6.1,7.7)	3.0(1.0,8.3)	5.6(4.9,6.3)
Odisha	Sundargarh	23.9(16.7,32.9)	20.1(19.6,20.6)	3.3(1.6,6.9)	10.4(10.0,10.8)	5.4(2.1,13.2)	3.7(3.5,4.0)
Odisha	Kendujhar	18.0(8.5,34.0)	25.9(25.3,26.5)	9.9(4.3,21.2)	12.6(12.1,13.0)	4.2(1.5,11.1)	5.9(5.6,6.2)
Odisha	Mayurbhanj	20.7(11.3,34.8)	23.8(23.3,24.3)	10.4(5.9,17.6)	9.4(9.0,9.7)	14.3(8.4,23.5)	7.7(7.4,8.0)
Odisha	Baleshwar	25.9(16.0,39.0)	27.1(26.6,27.6)	12.4(6.8,21.8)	9.6(9.2,9.9)	5.9(2.8,11.9)	6.8(6.5,7.1)
Odisha	Bhadrak	26.1(17.5,37.1)	26.6(25.9,27.2)	14.9(7.5,27.5)	13.5(13.0,14.0)	6.0(3.0,11.8)	6.5(6.1,6.9)
Odisha	Kendrapara	15.2(7.9,27.3)	16.1(15.6,16.7)	9.4(4.1,20.1)	6.3(5.9,6.6)	10.7(5.6,19.2)	5.3(4.9,5.6)
Odisha	Jagatsinghapur	11.8(6.9,19.4)	21.0(20.3,21.6)	5.7(3.1,10.5)	7.9(7.4,8.3)	9.3(3.4,23.3)	7.1(6.7,7.6)
Odisha	Cuttack	13.0(8.6,19.1)	21.2(20.8,21.7)	4.5(1.4,13.9)	9.0(8.6,9.3)	1.6(0.4,5.5)	4.7(4.5,4.9)
Odisha	Jajapur	25.7(14.6,41.1)	24.9(24.3,25.5)	9.9(5.2,18.0)	7.5(7.1,7.8)	2.3(0.8,6.2)	3.0(2.8,3.2)
Odisha	Dhenkanal	41.4(30.0,53.9)	29.3(28.6,30.1)	17.0(9.7,28.0)	9.8(9.3,10.3)	8.8(4.1,18.1)	7.0(6.6,7.4)
Odisha	Anugul	32.1(24.6,40.6)	27.7(27.0,28.4)	6.5(2.8,14.8)	11.8(11.3,12.3)	6.7(2.5,16.6)	10.8(10.3,11.3)
Odisha	Nayagarh	31.6(22.5,42.4)	29.8(29.0,30.7)	9.4(5.0,16.9)	9.7(9.2,10.2)	5.3(3.3,8.4)	6.8(6.4,7.3)
Odisha	Khordha	21.1(12.8,32.9)	18.1(17.6,18.5)	8.4(4.1,16.5)	8.6(8.3,8.9)	1.0(0.3,3.7)	4.2(4.0,4.4)
Odisha	Puri	25.4(16.3,37.4)	21.8(21.2,22.4)	11.6(6.2,20.8)	7.7(7.3,8.1)	9.9(5.8,16.1)	6.0(5.7,6.3)
Odisha	Ganjam	20.1(13.6,28.6)	19.0(18.7,19.4)	11.4(5.0,23.8)	7.2(6.9,7.4)	1.4(0.3,5.8)	5.4(5.2,5.7)
Odisha	Gajapati	21.5(12.9,33.8)	22.3(21.3,23.3)	10.7(4.6,23.0)	12.3(11.5,13.1)	8.2(3.2,19.4)	5.3(4.8,5.8)
Odisha	Kandhamal	27.8(15.8,44.1)	23.7(22.7,24.6)	9.7(5.4,16.9)	9.8(9.2,10.5)	22.9(8.9,47.2)	9.8(9.1,10.4)
Odisha	Baudh	29.2(19.2,41.7)	27.7(26.5,28.9)	5.3(2.6,10.5)	8.2(7.4,8.9)	5.4(2.6,10.6)	5.7(5.1,6.3)
Odisha	Subarnapur	20.5(11.8,33.2)	23.4(22.4,24.4)	3.9(1.8,8.3)	9.0(8.3,9.7)	2.1(0.3,12.8)	5.5(5.0,6.1)
Odisha	Balangir	32.8(23.1,44.1)	22.9(22.3,23.5)	11.9(5.3,24.7)	6.7(6.3,7.0)	3.8(0.8,16.8)	6.3(6.0,6.7)
Odisha	Nuapada	26.7(15.0,42.8)	25.6(24.6,26.6)	9.7(2.8,28.6)	10.6(9.9,11.3)	7.4(1.9,24.7)	9.2(8.5,9.9)
Odisha	Kalahandi	18.1(11.4,27.5)	27.6(27.0,28.2)	6.2(2.2,16.2)	10.0(9.5,10.4)	1.1(0.2,7.0)	6.0(5.6,6.3)
Odisha	Rayagada	24.1(13.8,38.7)	28.6(27.8,29.4)	11.9(4.6,27.7)	10.1(9.5,10.6)	1.4(0.4,5.5)	6.8(6.3,7.3)
Odisha	Nabarangapur	28.7(20.8,38.0)	33.6(32.8,34.3)	5.0(2.1,11.1)	9.3(8.8,9.8)	7.0(2.5,17.9)	6.8(6.4,7.2)
Odisha	Koraput	28.2(20.4,37.5)	27.2(26.5,27.9)	8.8(4.2,17.7)	9.5(9.1,10.0)	10.9(5.3,21.4)	6.6(6.3,7.0)
Odisha	Malkangiri	34.5(24.6,45.9)	30.6(29.6,31.7)	12.5(7.3,20.6)	8.4(7.8,9.1)	2.9(1.0,8.3)	5.1(4.6,5.6)
Chhattisgarh	Koriya	25.3(16.6,36.5)	23.3(22.3,24.2)	14.5(9.6,21.2)	12.6(11.9,13.4)	5.0(2.3,10.5)	3.3(2.9,3.7)
Chhattisgarh	Surguja	35.1(24.3,47.7)	30.1(29.6,30.7)	16.6(9.6,27.1)	16.1(15.7,16.6)	11.5(5.2,23.7)	7.8(7.5,8.1)
Chhattisgarh	Jashpur	34.3(22.5,48.5)	30.9(30.0,31.8)	6.4(2.3,16.3)	10.9(10.3,11.5)	6.9(2.5,17.5)	4.4(4.0,4.8)
Chhattisgarh	Raigarh	28.6(17.3,43.3)	28.1(27.4,28.7)	9.8(5.3,17.3)	10.0(9.6,10.4)	6.2(2.8,13.4)	3.9(3.6,4.2)
Chhattisgarh	Korba	22.6(15.7,31.3)	28.0(27.3,28.7)	10.8(6.3,17.9)	11.2(10.7,11.7)	4.2(1.8,9.6)	3.7(3.4,4.0)
Chhattisgarh	Janjgir - Champa	28.3(15.4,46.2)	27.8(27.1,28.4)	14.5(7.7,25.6)	12.6(12.1,13.1)	3.8(1.1,12.4)	5.4(5.1,5.7)
Chhattisgarh	Bilaspur	27.7(20.8,35.8)	24.9(24.4,25.4)	8.2(3.6,17.4)	10.6(10.2,10.9)	3.3(1.2,9.3)	5.3(5.0,5.5)
Chhattisgarh	Kabeerdham	31.1(22.7,41.0)	29.7(28.8,30.6)	19.5(11.4,31.5)	16.5(15.7,17.2)	6.1(2.7,13.2)	6.8(6.4,7.3)
Chhattisgarh	Rajnandgaon	22.2(14.4,32.7)	18.4(17.9,19.0)	9.0(4.8,16.2)	8.6(8.2,9.0)	1.2(0.4,3.3)	3.5(3.3,3.8)
Chhattisgarh	Durg	24.1(15.9,34.7)	24.9(24.5,25.3)	9.7(5.7,16.2)	12.3(12.0,12.6)	1.4(0.5,3.9)	4.3(4.1,4.5)
Chhattisgarh	Raipur	17.7(11.2,26.8)	24.0(23.6,24.3)	4.7(2.6,8.4)	10.4(10.1,10.7)	2.3(1.2,4.7)	4.1(3.9,4.3)
Chhattisgarh	Mahasamund	29.3(18.1,43.8)	27.2(26.5,28.0)	14.8(7.8,26.1)	11.9(11.4,12.5)	4.8(1.1,18.6)	4.3(3.9,4.6)
Chhattisgarh	Dhamtari	17.6(9.0,31.4)	24.4(23.5,25.2)	17.7(9.5,30.5)	13.9(13.2,14.6)	7.8(3.2,18.1)	5.2(4.8,5.7)
Chhattisgarh	Uttar Bastar Kanker	28.2(17.9,41.5)	24.4(23.5,25.3)	7.7(2.9,19.1)	8.9(8.3,9.5)	1.7(0.4,6.7)	4.7(4.2,5.1)

Continued…

Table A2: Direct-survey based estimates and model-based estimates of physical,emotional and sexual violence in districts of India, 2015-16.

State name	District name	Physical violence: Direct-survey based estimates	Physical violence: Model-based estimates	Emotional violence: Direct-survey based estimates	Emotional violence: Model-based estimates	Sexual violence: Direct-survey based estimates	Sexual violence: Model-based estimates
		Estimates (Lower bound, Upper bound)	Estimates (Lower bound, Upper bound)	Estimates (Lower bound, Upper bound)	Estimates (Lower bound, Upper bound)	Estimates (Lower bound, Upper bound)	Estimates (Lower bound, Upper bound)
Chhattisgarh	Bastar	32.9(20.6,48.1)	29.5(28.8,30.2)	30.7(20.6,43.1)	18.0(17.4,18.6)	7.3(3.6,14.2)	4.6(4.3,4.9)
Chhattisgarh	Narayanpur	22.7(13.2,36.2)	33.8(31.4,36.2)	5.5(2.0,14.6)	16.6(14.7,18.5)	0.6(0.1,4.2)	6.5(5.2,7.7)
Chhattisgarh	Dakshin Bastar Dantewada	39.8(23.4,59.0)	35.5(34.3,36.7)	22.5(10.5,41.8)	14.4(13.6,15.3)	8.9(2.3,28.7)	4.2(3.7,4.7)
Chhattisgarh	Bijapur	32.0(20.7,45.7)	34.8(33.0,36.5)	10.3(5.2,19.3)	17.3(15.9,18.7)	5.1(1.6,15.0)	6.7(5.8,7.6)
Madhya Pradesh	Sheopur	33.2(24.9,42.7)	31.6(30.6,32.6)	7.9(3.0,19.2)	11.9(11.2,12.6)	3.5(1.3,9.0)	9.2(8.6,9.9)
Madhya Pradesh	Morena	39.8(25.5,56.1)	33.1(32.5,33.7)	12.9(5.9,26.0)	10.9(10.5,11.3)	8.8(4.1,17.8)	6.7(6.4,7.1)
Madhya Pradesh	Bhind	25.0(16.2,36.3)	30.0(29.3,30.6)	5.1(2.2,11.7)	9.8(9.4,10.3)	0.0(0.0,0.0)	4.8(4.5,5.0)
Madhya Pradesh	Gwalior	35.0(24.2,47.6)	18.7(18.2,19.2)	13.2(6.6,24.7)	7.5(7.2,7.8)	7.5(2.2,22.4)	4.9(4.7,5.2)
Madhya Pradesh	Datia	31.3(21.3,43.4)	24.5(23.7,25.4)	13.5(7.4,23.4)	8.6(8.1,9.2)	8.4(2.6,24.4)	6.7(6.2,7.2)
Madhya Pradesh	Shivpuri	24.7(16.0,36.2)	21.3(20.7,21.9)	9.9(5.4,17.5)	11.2(10.7,11.6)	7.0(3.6,13.1)	5.0(4.7,5.3)
Madhya Pradesh	Tikamgarh	30.7(15.9,51.0)	32.8(32.1,33.5)	11.7(6.4,20.4)	13.4(12.9,13.9)	15.6(8.4,27.1)	9.5(9.1,9.9)
Madhya Pradesh	Chhatarpur	33.4(21.5,48.0)	29.7(29.1,30.4)	17.8(10.3,29.0)	12.5(12.1,13.0)	3.8(1.1,11.6)	9.4(9.0,9.8)
Madhya Pradesh	Panna	19.3(10.0,34.0)	25.3(24.5,26.1)	7.8(2.7,20.5)	10.7(10.1,11.2)	2.8(0.7,11.2)	6.2(5.8,6.7)
Madhya Pradesh	Sagar	9.8(3.9,22.4)	27.6(27.0,28.1)	6.9(3.6,12.9)	13.0(12.6,13.4)	7.3(2.2,21.5)	10.0(9.6,10.3)
Madhya Pradesh	Damoh	32.0(17.9,50.4)	24.8(24.1,25.5)	16.1(6.6,34.4)	9.9(9.4,10.4)	12.9(5.3,28.1)	5.5(5.1,5.9)
Madhya Pradesh	Satna	16.0(7.8,30.2)	18.9(18.4,19.4)	7.1(2.7,17.3)	7.6(7.3,7.9)	7.8(1.5,31.4)	5.6(5.3,5.8)
Madhya Pradesh	Rewa	31.2(19.2,46.4)	28.1(27.5,28.6)	12.1(6.1,22.6)	10.9(10.5,11.3)	9.5(5.8,15.1)	7.8(7.5,8.1)
Madhya Pradesh	Umaria	27.0(17.5,39.2)	28.2(27.2,29.1)	16.4(10.6,24.5)	14.6(13.8,15.4)	12.3(5.9,23.6)	6.7(6.1,7.2)
Madhya Pradesh	Neemuch	30.9(24.3,38.3)	20.8(20.1,21.6)	9.9(5.4,17.4)	8.1(7.6,8.6)	13.7(8.9,20.5)	5.6(5.1,6.0)
Madhya Pradesh	Mandsaur	17.6(12.5,24.3)	23.6(22.9,24.2)	10.1(5.0,19.5)	10.2(9.7,10.6)	5.8(2.0,15.9)	5.2(4.8,5.5)
Madhya Pradesh	Ratlam	37.7(26.1,51.0)	24.8(24.2,25.4)	23.6(10.9,43.8)	10.8(10.4,11.3)	6.8(3.5,12.6)	5.9(5.5,6.2)
Madhya Pradesh	Ujjain	16.8(10.2,26.3)	21.5(21.0,22.0)	11.9(7.2,19.0)	7.8(7.5,8.2)	8.3(4.9,13.8)	3.0(2.8,3.3)
Madhya Pradesh	Shajapur	22.7(13.6,35.5)	26.5(25.9,27.1)	15.7(6.3,34.1)	10.4(10.0,10.9)	3.5(1.2,9.7)	5.8(5.5,6.1)
Madhya Pradesh	Dewas	31.7(20.2,46.0)	24.8(24.2,25.4)	14.3(7.7,25.0)	10.7(10.3,11.2)	11.0(4.8,23.2)	6.9(6.6,7.3)
Madhya Pradesh	Dhar	36.3(25.2,49.1)	26.3(25.8,26.9)	7.9(3.7,16.2)	10.6(10.2,10.9)	11.4(6.5,19.1)	7.4(7.1,7.7)
Madhya Pradesh	Indore	19.0(12.2,28.5)	16.2(15.8,16.5)	14.8(7.1,28.2)	8.1(7.8,8.3)	5.5(2.9,10.3)	2.8(2.6,3.0)
Madhya Pradesh	West Nimar (Khargone)	41.4(30.3,53.4)	28.4(27.8,28.9)	15.2(9.6,23.1)	11.5(11.1,11.9)	2.6(0.7,9.2)	4.5(4.2,4.7)
Madhya Pradesh	Barwani	19.4(11.2,31.4)	36.8(36.1,37.5)	6.8(2.4,18.3)	15.6(15.0,16.1)	1.3(0.3,6.2)	9.8(9.3,10.2)
Madhya Pradesh	Rajgarh	16.8(9.7,27.6)	28.1(27.5,28.7)	1.6(0.4,5.9)	13.0(12.5,13.5)	0.9(0.1,6.5)	6.9(6.6,7.3)
Madhya Pradesh	Vidisha	29.6(17.6,45.2)	30.1(29.4,30.8)	8.0(4.0,15.5)	9.6(9.2,10.1)	7.2(2.5,19.0)	8.4(8.0,8.9)
Madhya Pradesh	Bhopal	23.6(12.7,39.7)	17.4(16.9,17.8)	19.0(8.3,37.8)	9.2(8.9,9.6)	6.3(2.8,13.5)	6.0(5.7,6.2)
Madhya Pradesh	Sehore	20.3(12.0,32.2)	26.6(25.9,27.3)	4.1(1.5,10.3)	12.6(12.1,13.2)	4.6(1.9,10.7)	7.9(7.5,8.4)
Madhya Pradesh	Raisen	21.9(11.9,36.8)	24.8(24.1,25.5)	9.7(3.8,22.6)	9.6(9.1,10.0)	3.6(1.1,10.6)	7.5(7.1,7.9)
Madhya Pradesh	Betul	20.6(12.5,31.9)	26.6(26.0,27.3)	8.9(5.0,15.3)	9.5(9.0,9.9)	7.2(4.1,12.6)	4.4(4.1,4.7)
Madhya Pradesh	Harda	18.4(8.2,36.1)	26.7(25.7,27.8)	4.7(1.8,11.9)	10.9(10.2,11.6)	5.0(1.7,14.1)	7.5(6.9,8.1)
Madhya Pradesh	Hoshangabad	22.4(14.1,33.8)	24.0(23.3,24.7)	6.0(2.7,12.8)	10.8(10.3,11.3)	2.4(0.7,7.5)	5.4(5.0,5.7)
Madhya Pradesh	Katni	14.9(6.8,29.8)	23.2(22.5,23.8)	7.6(3.7,14.8)	10.0(9.6,10.5)	3.9(1.1,12.3)	7.0(6.6,7.4)
Madhya Pradesh	Jabalpur	27.1(20.4,35.1)	17.4(17.0,17.9)	10.0(5.2,18.4)	9.2(8.9,9.5)	4.1(1.7,9.7)	4.0(3.8,4.2)
Madhya Pradesh	Narsimhapur	11.3(7.5,16.7)	19.0(18.3,19.7)	13.2(5.5,28.5)	10.0(9.5,10.5)	3.7(1.2,10.9)	4.2(3.8,4.5)
Madhya Pradesh	Dindori	25.2(13.7,41.5)	27.8(26.9,28.7)	7.3(3.5,14.6)	8.8(8.2,9.3)	1.5(0.5,4.6)	4.6(4.2,5.1)
Madhya Pradesh	Mandla	42.7(26.8,60.4)	28.1(27.3,28.9)	3.6(1.3,9.6)	10.0(9.5,10.5)	1.4(0.4,5.2)	4.2(3.8,4.5)
Madhya Pradesh	Chhindwara	28.9(19.7,40.2)	21.3(20.8,21.8)	17.7(10.4,28.5)	12.4(12.0,12.9)	7.8(4.3,13.6)	3.2(3.0,3.4)
Madhya Pradesh	Seoni	9.7(4.7,18.8)	24.3(23.7,25.0)	3.7(1.6,8.0)	8.5(8.1,8.9)	2.5(0.5,10.9)	4.6(4.3,4.9)
Madhya Pradesh	Balaghat	16.3(7.3,32.5)	19.5(18.9,20.0)	7.2(2.8,16.9)	6.8(6.5,7.1)	0.8(0.1,5.5)	4.8(4.5,5.1)
Madhya Pradesh	Guna	30.6(16.8,49.1)	29.8(29.0,30.5)	10.8(5.8,19.2)	10.9(10.4,11.4)	2.1(0.7,6.4)	5.0(4.6,5.3)
Madhya Pradesh	Ashoknagar	40.2(26.5,55.6)	31.6(30.7,32.5)	18.9(12.1,28.2)	12.2(11.6,12.8)	14.9(8.6,24.6)	9.8(9.3,10.4)
Madhya Pradesh	Shahdol	20.5(12.0,32.6)	25.7(24.9,26.4)	8.4(4.0,16.9)	9.7(9.2,10.2)	5.7(1.9,15.9)	5.3(4.9,5.6)
Madhya Pradesh	Anuppur	25.0(13.7,41.2)	24.6(23.7,25.4)	11.6(6.1,21.1)	9.8(9.2,10.4)	4.9(1.3,16.8)	7.1(6.6,7.6)
Madhya Pradesh	Sidhi	14.6(8.9,23.0)	25.7(25.0,26.5)	7.4(5.4,10.2)	10.3(9.8,10.8)	3.6(1.2,10.2)	5.9(5.5,6.3)
Madhya Pradesh	Singrauli	33.2(26.1,41.1)	27.6(26.8,28.3)	13.5(7.4,23.4)	10.9(10.4,11.4)	1.9(0.6,6.0)	6.5(6.1,7.0)
Madhya Pradesh	Jhabua	22.7(13.9,34.8)	28.5(27.7,29.3)	6.9(4.2,11.2)	9.9(9.3,10.4)	19.2(10.2,33.1)	8.0(7.5,8.5)
Madhya Pradesh	Alirajpur	31.5(19.3,47.0)	27.7(26.7,28.7)	8.2(3.6,17.7)	11.9(11.2,12.6)	8.1(3.5,17.5)	8.3(7.7,8.9)
Madhya Pradesh	East Nimar(Khandwa)	33.8(19.9,51.1)	27.1(26.3,27.8)	14.6(6.6,29.2)	10.5(10.0,11.0)	7.0(2.4,18.8)	4.2(3.9,4.6)
Madhya Pradesh	Burhanpur	14.3(10.2,19.6)	20.9(20.0,21.7)	3.0(1.5,5.9)	11.0(10.3,11.6)	1.6(0.5,5.2)	3.3(2.9,3.7)
Gujarat	Kachchh	2.3(0.7,7.2)	14.9(14.5,15.4)	10.6(4.7,22.3)	14.7(14.3,15.2)	1.1(0.3,4.3)	4.1(3.9,4.4)
Gujarat	Banas Kantha	18.5(12.1,27.3)	15.8(15.4,16.1)	8.3(3.6,18.0)	9.1(8.8,9.4)	1.1(0.2,7.5)	3.1(2.9,3.2)
Gujarat	Patan	26.7(16.4,40.2)	16.2(15.7,16.8)	18.0(10.1,30.0)	10.9(10.4,11.4)	12.3(6.6,22.0)	5.2(4.9,5.5)
Gujarat	Mahesana	14.7(8.9,23.3)	13.4(13.0,13.8)	3.8(1.1,12.2)	8.2(7.9,8.6)	2.0(0.5,8.3)	2.8(2.6,3.0)
Gujarat	Sabar Kantha	19.4(11.1,31.7)	15.0(14.6,15.4)	5.3(2.4,11.1)	8.0(7.7,8.3)	3.0(1.0,8.4)	2.5(2.3,2.7)

Continued…

State name	District name	Physical violence: Direct-survey based estimates – Estimates (Lower bound, Upper bound)	Physical violence: Model-based estimates – Estimates (Lower bound, Upper bound)	Emotional violence: Direct-survey based estimates – Estimates (Lower bound, Upper bound)	Emotional violence: Model-based estimates – Estimates (Lower bound, Upper bound)	Sexual violence: Direct-survey based estimates – Estimates (Lower bound, Upper bound)	Sexual violence: Model-based estimates – Estimates (Lower bound, Upper bound)
Gujarat	Gandhinagar	5.0(2.2,11.0)	12.1(11.7,12.6)	11.5(4.3,27.1)	9.2(8.8,9.6)	2.1(0.7,6.6)	2.2(2.0,2.4)
Gujarat	Ahmadabad	10.8(6.1,18.5)	9.7(9.5,9.9)	7.0(4.1,11.8)	6.7(6.5,6.8)	1.5(0.4,5.7)	2.6(2.5,2.7)
Gujarat	Surendranagar	27.8(17.1,41.7)	17.8(17.3,18.3)	14.9(6.4,31.0)	12.7(12.2,13.1)	10.2(2.8,31.2)	4.9(4.6,5.2)
Gujarat	Rajkot	9.2(5.3,15.4)	10.3(10.0,10.6)	1.4(0.2,8.9)	8.7(8.4,8.9)	0.7(0.1,5.2)	1.8(1.6,1.9)
Gujarat	Jamnagar	1.8(0.7,4.5)	10.5(10.1,10.9)	5.9(2.5,13.4)	9.3(8.9,9.6)	0.0(0.0,0.0)	3.7(3.5,4.0)
Gujarat	Porbandar	4.9(1.9,12.3)	9.9(9.2,10.6)	6.6(3.2,12.9)	7.2(6.6,7.8)	1.3(0.3,5.2)	2.8(2.5,3.2)
Gujarat	Junagadh	25.1(12.3,44.4)	18.5(18.1,18.9)	12.9(5.4,27.7)	14.7(14.3,15.1)	11.8(4.0,30.2)	5.8(5.5,6.0)
Gujarat	Amreli	16.0(8.8,27.3)	19.4(18.8,20.0)	12.3(6.0,23.8)	14.9(14.4,15.4)	2.4(0.9,6.3)	5.2(4.9,5.6)
Gujarat	Bhavnagar	21.9(12.4,35.7)	16.9(16.5,17.3)	14.6(7.2,27.2)	12.0(11.7,12.4)	0.4(0.1,3.1)	3.4(3.2,3.6)
Gujarat	Anand	11.0(5.6,20.4)	13.8(13.4,14.2)	15.3(8.6,25.8)	9.7(9.3,10.0)	1.6(0.5,5.0)	2.4(2.2,2.6)
Gujarat	kheda	14.0(9.4,20.3)	12.9(12.6,13.3)	12.5(6.8,21.8)	8.3(8.0,8.6)	4.4(1.1,16.8)	2.1(1.9,2.2)
Gujarat	Panch Mahals	12.4(7.0,21.1)	13.4(13.0,13.8)	9.8(5.1,18.0)	10.8(10.4,11.1)	1.9(0.4,8.1)	1.8(1.7,2.0)
Gujarat	Dohad	21.9(13.8,33.0)	22.7(22.2,23.2)	15.9(10.0,24.3)	14.1(13.7,14.5)	3.1(1.0,9.5)	5.7(5.4,6.0)
Gujarat	Vadodara	8.8(4.4,17.0)	10.2(9.9,10.5)	2.7(1.1,6.4)	7.9(7.6,8.1)	0.5(0.1,3.1)	2.6(2.5,2.7)
Gujarat	Narmada	13.4(6.9,24.5)	14.2(13.4,15.0)	1.2(0.3,4.3)	7.4(6.8,8.0)	1.1(0.3,3.5)	2.6(2.3,3.0)
Gujarat	Bharuch	8.0(4.1,15.2)	12.3(11.8,12.8)	5.2(1.8,13.9)	8.3(7.9,8.6)	0.0(0.0,0.0)	1.7(1.5,1.9)
Gujarat	The Dangs	14.2(7.7,24.7)	15.8(14.4,17.2)	14.1(8.1,23.3)	11.0(9.8,12.2)	3.8(1.7,8.4)	3.5(2.8,4.2)
Gujarat	Navsari	20.7(11.3,34.9)	13.6(13.0,14.1)	19.0(10.1,33.0)	11.4(11.0,11.9)	2.8(0.6,12.8)	3.4(3.1,3.6)
Gujarat	Valsad	14.1(7.8,24.3)	15.1(14.6,15.6)	13.8(7.7,23.4)	12.5(12.1,13.0)	4.3(1.9,9.4)	4.0(3.7,4.2)
Gujarat	Surat	16.8(8.3,30.9)	9.8(9.6,10.0)	14.3(6.2,29.6)	7.6(7.4,7.8)	7.1(2.3,20.2)	1.8(1.7,1.9)
Gujarat	Tapi	7.8(4.8,12.3)	13.0(12.3,13.6)	5.2(3.1,8.8)	7.2(6.7,7.7)	3.1(1.3,7.1)	2.2(2.0,2.5)
Daman & Diu	Diu	3.1(0.9,10.2)	11.8(9.2,14.5)	4.5(2.1,9.7)	10.8(8.2,13.3)	0.0(0.0,0.0)	4.1(2.5,5.7)
Daman & Diu	Daman	26.3(16.6,39.1)	17.6(15.9,19.2)	13.7(7.4,23.9)	7.5(6.4,8.7)	5.1(2.1,11.6)	1.4(0.9,1.9)
Dadra & Nagar Haveli	Dadra & Nagar Haveli	16.9(12.9,22.0)	16.9(15.8,18.1)	11.3(5.3,22.3)	11.3(10.3,12.2)	2.6(1.0,6.6)	2.6(2.1,3.1)
Maharashtra	Nandurbar	13.3(7.2,23.3)	16.6(16.0,17.1)	10.3(5.5,18.6)	8.1(7.8,8.5)	1.2(0.2,8.2)	1.6(1.4,1.8)
Maharashtra	Dhule	27.5(12.7,49.7)	17.6(17.2,18.1)	21.6(8.8,44.1)	10.4(10.0,10.7)	1.0(0.1,6.4)	2.7(2.5,2.9)
Maharashtra	Jalgaon	10.8(6.3,17.9)	14.9(14.6,15.2)	4.5(2.6,7.8)	8.1(7.8,8.3)	3.0(0.8,10.4)	1.7(1.6,1.9)
Maharashtra	Buldana	18.9(12.9,26.7)	15.3(14.9,15.6)	7.2(2.7,17.8)	5.8(5.6,6.1)	3.3(0.9,11.6)	1.5(1.4,1.7)
Maharashtra	Akola	18.1(8.7,34.0)	14.8(14.3,15.3)	11.2(5.9,20.2)	7.0(6.6,7.3)	1.1(0.2,6.8)	1.4(1.2,1.5)
Maharashtra	Washim	18.1(9.9,30.8)	14.9(14.3,15.4)	9.9(5.0,18.5)	8.2(7.7,8.6)	4.0(1.7,9.5)	1.3(1.1,1.5)
Maharashtra	Amravati	13.9(8.8,21.4)	15.3(14.9,15.7)	2.8(0.6,12.3)	7.2(7.0,7.5)	1.0(0.1,7.0)	1.3(1.2,1.5)
Maharashtra	Wardha	11.1(4.8,23.5)	18.6(18.0,19.2)	3.1(0.6,14.0)	11.2(10.7,11.7)	2.4(0.3,15.4)	3.6(3.3,3.9)
Maharashtra	Nagpur	7.7(3.1,18.0)	15.2(14.9,15.4)	4.6(1.7,11.7)	5.4(5.3,5.6)	0.0(0.0,0.0)	1.6(1.5,1.7)
Maharashtra	Bhandara	9.4(3.2,24.7)	13.9(13.3,14.5)	3.4(1.1,10.5)	5.5(5.1,5.8)	1.8(0.4,7.3)	1.4(1.2,1.6)
Maharashtra	Gondiya	18.6(6.8,41.4)	19.5(18.9,20.2)	11.3(5.2,23.0)	6.8(6.4,7.2)	1.6(0.2,10.3)	1.5(1.3,1.7)
Maharashtra	Gadchiroli	13.2(9.3,18.4)	18.1(17.4,18.7)	6.5(4.3,9.6)	7.6(7.2,8.1)	0.0(0.0,0.0)	2.2(2.0,2.5)
Maharashtra	Chandrapur	7.8(3.4,16.8)	14.9(14.5,15.3)	0.0(0.0,0.0)	9.3(9.0,9.7)	0.7(0.1,5.1)	2.4(2.3,2.6)
Maharashtra	Yavatmal	17.7(10.5,28.3)	15.1(14.8,15.5)	7.0(3.8,12.2)	5.9(5.6,6.1)	1.7(0.5,6.4)	1.8(1.7,2.0)
Maharashtra	Nanded	22.5(13.1,36.0)	17.8(17.4,18.2)	9.2(4.9,16.7)	10.1(9.8,10.4)	0.0(0.0,0.0)	1.5(1.4,1.6)
Maharashtra	Hingoli	30.0(20.8,41.2)	19.9(19.3,20.6)	7.5(2.9,17.9)	8.0(7.5,8.4)	5.0(1.9,12.4)	2.2(2.0,2.5)
Maharashtra	Parbhani	20.2(11.9,32.3)	20.1(19.6,20.6)	5.2(3.3,8.3)	7.6(7.2,7.9)	0.6(0.1,3.8)	2.2(2.0,2.4)
Maharashtra	Jalna	19.1(10.5,32.2)	15.4(15.0,15.9)	11.1(4.8,23.6)	9.8(9.4,10.2)	0.0(0.0,0.0)	2.0(1.8,2.1)
Maharashtra	Aurangabad	23.1(15.9,32.3)	18.1(17.8,18.5)	10.9(6.3,18.3)	9.3(9.0,9.5)	9.1(5.3,15.1)	2.2(2.1,2.3)
Maharashtra	Nashik	12.9(8.0,19.9)	21.4(21.1,21.7)	5.7(2.0,15.0)	8.7(8.5,8.9)	1.9(0.4,8.1)	1.5(1.4,1.5)
Maharashtra	Thane	7.9(1.7,29.3)	14.6(14.4,14.7)	9.5(4.2,20.1)	8.5(8.4,8.7)	0.0(0.0,0.0)	2.2(2.1,2.2)
Maharashtra	Mumbai Suburban	12.1(4.3,29.6)	12.0(11.8,12.2)	10.6(4.9,21.3)	6.4(6.2,6.5)	1.2(0.2,7.3)	0.5(0.5,0.6)
Maharashtra	Mumbai	22.5(12.4,37.2)	11.1(10.8,11.4)	7.4(2.6,19.1)	5.5(5.3,5.7)	0.0(0.0,0.0)	0.5(0.4,0.6)
Maharashtra	Raigarh	7.8(1.7,29.3)	12.1(11.8,12.5)	2.9(0.7,10.4)	6.8(6.5,7.1)	1.3(0.2,9.3)	2.3(2.1,2.4)
Maharashtra	Pune	12.2(5.1,26.5)	12.6(12.4,12.8)	5.3(1.5,16.4)	6.4(6.3,6.6)	0.8(0.1,5.7)	1.8(1.7,1.8)
Maharashtra	Ahmadnagar	17.5(9.4,30.3)	15.6(15.3,15.9)	9.3(3.9,20.6)	8.9(8.7,9.1)	3.3(1.1,9.4)	1.6(1.5,1.7)
Maharashtra	Bid	24.5(11.3,45.1)	16.9(16.5,17.3)	14.9(8.1,26.0)	6.8(6.5,7.0)	7.6(2.7,19.2)	1.9(1.8,2.1)
Maharashtra	Latur	20.7(11.4,34.5)	18.3(17.9,18.8)	10.4(5.1,20.2)	7.9(7.6,8.2)	2.2(0.4,10.2)	1.7(1.6,1.9)
Maharashtra	Osmanabad	16.3(8.0,30.4)	17.9(17.4,18.5)	8.6(4.7,15.3)	13.8(13.4,14.3)	2.4(0.7,8.3)	2.1(1.9,2.3)
Maharashtra	Solapur	17.9(10.9,27.9)	18.7(18.4,19.1)	8.1(2.8,21.4)	8.4(8.1,8.6)	0.0(0.0,0.0)	2.9(2.8,3.1)
Maharashtra	Satara	10.5(5.8,18.2)	14.0(13.6,14.3)	6.1(3.8,9.6)	9.1(8.8,9.4)	0.8(0.1,5.7)	1.3(1.2,1.4)
Maharashtra	Ratnagiri	16.8(11.7,23.5)	10.4(10.0,10.8)	12.0(4.9,26.8)	5.0(4.7,5.3)	2.9(0.4,17.9)	2.5(2.3,2.7)
Maharashtra	Sindhudurg	6.0(2.8,12.4)	14.5(13.8,15.2)	0.0(0.0,0.0)	7.4(6.9,7.9)	2.3(0.5,10.2)	2.6(2.3,3.0)
Maharashtra	Kolhapur	16.4(10.4,25.0)	16.2(15.9,16.5)	7.7(3.2,17.5)	7.3(7.1,7.5)	0.0(0.0,0.0)	1.9(1.8,2.0)
Maharashtra	Sangli	20.2(13.9,28.5)	21.0(20.5,21.4)	11.6(5.6,22.4)	9.3(9.0,9.6)	1.5(0.2,9.4)	2.4(2.2,2.5)

Table A2: Direct-survey based estimates and model-based estimates of physical,emotional and sexual violence in districts of India, 2015-16.

Continued…

Table A2: Direct-survey based estimates and model-based estimates of physical,emotional and sexual violence in districts of India, 2015-16.

State name	District name	Physical violence		Emotional violence		Sexual violence	
		Direct-survey based estimates	Model-based estimates	Direct-survey based estimates	Model-based estimates	Direct-survey based estimates	Model-based estimates
		Estimates (Lower bound, Upper bound)	Estimates (Lower bound, Upper bound)	Estimates (Lower bound, Upper bound)	Estimates (Lower bound, Upper bound)	Estimates (Lower bound, Upper bound)	Estimates (Lower bound, Upper bound)
Telangana	Adilabad	25.3(15.0,39.4)	36.3(35.8,36.8)	14.5(5.6,32.6)	16.7(16.3,17.1)	4.4(1.0,17.9)	5.6(5.3,5.8)
Telangana	Nizamabad	30.6(20.0,43.8)	36.9(36.4,37.5)	11.7(4.9,25.3)	19.2(18.7,19.6)	10.1(3.8,24.0)	7.7(7.4,8.0)
Telangana	Karimnagar	18.1(9.2,32.6)	28.0(27.6,28.4)	13.6(5.2,30.9)	14.9(14.6,15.3)	3.7(0.8,15.7)	5.7(5.5,5.9)
Telangana	Medak	35.8(25.5,47.6)	35.4(34.9,35.9)	21.6(17.3,26.5)	23.5(23.1,23.9)	12.4(7.5,19.9)	7.1(6.9,7.4)
Telangana	Hyderabad	26.9(13.4,46.8)	27.9(27.5,28.3)	8.0(3.3,18.4)	14.8(14.5,15.1)	0.0(0.0,0.0)	1.9(1.7,2.0)
Telangana	Rangareddy	43.7(27.2,61.8)	35.0(34.6,35.3)	10.8(5.7,19.4)	14.1(13.9,14.4)	1.7(0.9,3.0)	4.6(4.5,4.8)
Telangana	Mahbubnagar	36.7(26.3,48.4)	33.4(33.0,33.8)	33.5(21.3,48.3)	22.0(21.7,22.4)	1.3(0.2,8.7)	4.0(3.8,4.2)
Telangana	Nalgonda	47.8(33.6,62.5)	32.9(32.5,33.4)	22.4(13.6,34.7)	15.0(14.6,15.3)	10.9(5.9,19.2)	6.9(6.7,7.1)
Telangana	Warangal	34.7(22.2,49.7)	35.4(35.0,35.9)	20.2(11.7,32.5)	16.8(16.5,17.1)	6.9(2.7,16.5)	5.9(5.6,6.1)
Telangana	Khammam	36.7(25.3,49.8)	35.2(34.7,35.6)	14.0(7.7,24.3)	13.2(12.9,13.6)	1.2(0.2,8.5)	3.4(3.2,3.6)
Andhra Pradesh	Srikakulam	25.2(15.0,39.1)	29.3(28.8,29.8)	7.9(3.2,18.4)	12.5(12.1,12.8)	3.2(0.7,13.0)	4.4(4.2,4.6)
Andhra Pradesh	Vizianagaram	39.7(21.6,61.2)	33.7(33.2,34.2)	18.0(10.7,28.6)	12.3(12.0,12.7)	3.4(0.7,16.1)	2.5(2.4,2.7)
Andhra Pradesh	Visakhapatnam	23.3(18.0,29.6)	33.6(33.2,34.0)	16.0(10.0,24.7)	20.1(19.8,20.4)	10.8(5.1,21.5)	4.9(4.7,5.0)
Andhra Pradesh	East Godavari	43.6(30.2,58.0)	38.6(38.3,39.0)	24.7(17.4,33.8)	22.1(21.8,22.4)	4.4(1.0,16.8)	5.5(5.3,5.7)
Andhra Pradesh	West Godavari	36.3(22.9,52.2)	33.4(33.0,33.8)	16.7(7.3,33.9)	16.3(16.0,16.6)	3.8(0.5,22.4)	3.5(3.3,3.7)
Andhra Pradesh	Krishna	18.5(8.2,36.7)	33.5(33.1,33.8)	10.4(3.1,29.8)	16.3(16.0,16.6)	2.2(0.3,14.6)	2.7(2.6,2.8)
Andhra Pradesh	Guntur	30.6(18.3,46.5)	25.1(24.7,25.4)	12.7(5.9,25.3)	12.0(11.8,12.2)	4.0(1.4,10.7)	3.2(3.0,3.3)
Andhra Pradesh	Prakasam	19.7(10.1,34.9)	30.9(30.5,31.3)	5.0(2.5,9.7)	13.4(13.1,13.7)	4.8(2.1,10.4)	4.8(4.6,5.0)
Andhra Pradesh	Sri Potti Sriramulu Nellore	44.0(31.4,57.4)	37.9(37.4,38.4)	27.8(18.8,39.0)	22.3(21.9,22.7)	4.2(1.5,11.0)	6.7(6.5,7.0)
Andhra Pradesh	Y.S.R.	30.7(17.0,48.9)	36.4(35.9,36.8)	10.2(3.9,24.1)	16.2(15.8,16.5)	1.2(0.3,4.6)	4.3(4.1,4.5)
Andhra Pradesh	Kurnool	40.4(28.8,53.2)	32.2(31.8,32.6)	14.0(6.8,26.5)	15.5(15.2,15.8)	1.2(0.2,7.5)	2.5(2.4,2.7)
Andhra Pradesh	Anantapur	44.0(31.2,57.6)	35.7(35.3,36.1)	23.5(15.7,33.6)	17.2(16.8,17.5)	4.8(2.1,10.7)	4.8(4.6,5.0)
Andhra Pradesh	Chittoor	43.0(29.5,57.5)	38.7(38.3,39.1)	28.4(13.6,49.9)	19.0(18.7,19.3)	6.0(1.1,26.8)	4.2(4.0,4.4)
Karnataka	Belgaum	10.4(5.4,19.1)	22.3(22.0,22.6)	3.5(1.0,11.6)	12.2(11.9,12.4)	0.9(0.1,6.5)	5.1(4.9,5.2)
Karnataka	Bagalkot	16.1(8.9,27.4)	14.7(14.3,15.2)	14.2(9.2,21.2)	11.4(11.0,11.8)	4.9(1.7,13.3)	4.0(3.7,4.2)
Karnataka	Bijapur	19.9(7.8,42.0)	16.8(16.3,17.3)	11.1(3.3,31.1)	13.1(12.7,13.5)	10.4(3.4,28.1)	4.7(4.5,5.0)
Karnataka	Bidar	9.3(3.3,23.4)	21.2(20.6,21.8)	4.1(1.0,15.0)	10.7(10.2,11.1)	2.6(0.3,17.6)	5.5(5.2,5.8)
Karnataka	Raichur	13.9(5.6,30.5)	20.4(19.9,20.9)	6.8(2.6,16.8)	13.4(12.9,13.8)	4.6(0.9,19.7)	8.2(7.8,8.5)
Karnataka	Koppal	12.0(4.9,26.6)	15.4(14.9,16.0)	4.4(1.7,10.7)	12.3(11.8,12.8)	2.6(0.6,10.6)	4.1(3.8,4.4)
Karnataka	Gadag	31.9(12.0,61.6)	15.6(15.0,16.2)	15.5(5.3,37.7)	8.8(8.3,9.3)	5.7(0.7,34.4)	5.0(4.6,5.4)
Karnataka	Dharwad	20.1(11.5,32.9)	18.5(18.0,19.0)	6.9(2.9,15.6)	9.4(9.1,9.8)	2.5(0.5,11.5)	3.3(3.1,3.6)
Karnataka	Uttara Kannada	11.5(6.2,20.2)	18.0(17.5,18.6)	6.3(2.4,15.7)	10.1(9.7,10.6)	8.8(3.2,21.9)	4.9(4.6,5.2)
Karnataka	Haveri	12.3(5.3,25.9)	16.8(16.3,17.4)	3.9(0.9,15.3)	9.4(9.0,9.8)	7.0(1.9,22.7)	5.5(5.2,5.9)
Karnataka	Bellary	20.7(9.8,38.5)	21.0(20.6,21.5)	23.0(9.0,47.4)	15.4(15.0,15.8)	10.6(3.0,31.4)	8.5(8.2,8.8)
Karnataka	Chitradurga	26.4(10.5,52.4)	18.1(17.6,18.6)	21.6(8.1,46.3)	9.9(9.5,10.3)	9.8(2.7,29.7)	3.5(3.3,3.7)
Karnataka	Davanagere	15.9(11.6,21.3)	17.3(16.8,17.8)	19.5(10.6,33.2)	10.8(10.4,11.1)	4.7(1.3,16.0)	5.5(5.2,5.7)
Karnataka	Shimoga	9.9(4.6,20.0)	15.8(15.3,16.3)	14.1(6.9,26.8)	10.1(9.7,10.5)	3.7(0.5,22.5)	3.0(2.8,3.2)
Karnataka	Udupi	8.0(5.0,12.4)	10.0(9.5,10.5)	8.2(2.7,22.6)	6.1(5.7,6.5)	0.9(0.1,6.4)	2.5(2.3,2.8)
Karnataka	Chikmagalur	13.2(5.1,30.1)	19.2(18.6,19.9)	10.4(3.0,30.2)	11.7(11.2,12.2)	3.0(0.6,14.3)	4.9(4.6,5.3)
Karnataka	Tumkur	27.0(17.3,39.6)	15.2(14.8,15.5)	4.4(1.3,14.1)	7.9(7.6,8.2)	0.0(0.0,0.0)	2.2(2.0,2.3)
Karnataka	Bangalore	12.8(4.3,32.7)	7.4(7.2,7.5)	17.9(4.6,49.5)	10.6(10.4,10.8)	9.6(2.9,27.6)	5.0(4.9,5.1)
Karnataka	Mandya	20.1(9.3,38.2)	16.5(16.0,16.9)	6.4(2.2,17.2)	10.3(9.9,10.7)	9.6(2.0,36.0)	4.4(4.2,4.7)
Karnataka	Hassan	10.1(3.9,24.0)	13.5(13.0,13.9)	8.0(3.7,16.6)	9.0(8.6,9.3)	1.5(0.5,4.4)	2.3(2.1,2.5)
Karnataka	Dakshina Kannada	5.0(1.3,17.0)	11.8(11.4,12.2)	5.7(1.4,20.3)	8.5(8.1,8.8)	2.9(0.3,21.0)	3.2(3.0,3.4)
Karnataka	Kodagu	16.8(9.9,27.2)	17.0(16.1,17.9)	12.4(4.9,27.7)	10.9(10.1,11.6)	3.9(1.2,12.3)	3.3(2.9,3.8)
Karnataka	Mysore	20.3(11.1,34.0)	17.0(16.6,17.3)	21.2(8.2,44.8)	10.2(9.9,10.5)	5.2(1.5,16.7)	3.9(3.8,4.1)
Karnataka	Chamarajanagar	12.6(7.0,21.5)	17.4(16.8,18.1)	7.4(4.0,13.3)	10.0(9.5,10.5)	1.1(0.2,7.0)	2.6(2.4,2.9)
Karnataka	Gulbarga	26.3(8.8,56.7)	19.5(19.0,19.9)	8.6(3.3,20.4)	10.9(10.5,11.2)	3.3(0.7,14.7)	5.0(4.7,5.2)
Karnataka	Yadgir	24.5(10.5,47.5)	19.3(18.6,20.0)	13.3(6.5,25.3)	12.6(12.0,13.1)	2.7(0.6,11.5)	5.9(5.5,6.3)
Karnataka	Kolar	18.7(10.8,30.3)	15.9(15.4,16.4)	15.3(10.6,21.6)	12.6(12.1,13.1)	1.6(0.4,6.7)	3.1(2.9,3.4)
Karnataka	Chikkaballapura	21.9(10.9,39.2)	21.2(20.6,21.8)	5.4(2.1,13.0)	12.2(11.7,12.7)	0.0(0.0,0.0)	5.2(4.9,5.5)
Karnataka	Bangalore Rural	19.7(11.0,32.6)	17.4(16.8,18.1)	12.3(6.9,20.8)	10.7(10.2,11.2)	2.0(0.3,13.3)	4.1(3.8,4.5)
Karnataka	Ramanagara	20.1(11.4,33.1)	17.3(16.6,17.9)	10.1(4.6,20.6)	10.9(10.3,11.4)	5.5(1.8,15.5)	3.4(3.1,3.7)
Goa	North Goa	6.1(3.3,11.2)	9.9(9.3,10.5)	4.0(2.0,7.7)	4.8(4.4,5.2)	0.0(0.0,0.0)	0.1(0.1,0.2)
Goa	South Goa	12.6(7.6,20.3)	8.8(8.2,9.5)	5.3(3.4,8.2)	4.5(4.0,4.9)	0.3(0.0,2.0)	0.2(0.1,0.4)
Lakshadweep	Lakshadweep	4.2(1.6,10.8)	4.3(2.9,5.7)	3.1(0.9,10.1)	3.2(1.9,4.4)	0.6(0.1,3.8)	0.9(0.2,1.5)
Kerala	Kasaragod	7.0(3.1,14.8)	9.7(9.3,10.2)	2.7(0.9,7.5)	7.7(7.3,8.1)	0.8(0.1,4.9)	4.5(4.2,4.8)
Kerala	Kannur	4.4(0.8,19.6)	7.0(6.7,7.3)	1.7(0.4,6.8)	5.3(5.0,5.5)	0.0(0.0,0.0)	2.3(2.2,2.5)
Kerala	Wayanad	11.0(6.4,18.3)	11.9(11.3,12.5)	12.0(7.8,17.9)	9.1(8.6,9.7)	7.5(3.5,15.4)	4.6(4.2,5.0)

Continued…

Table A2: Direct-survey based estimates and model-based estimates of physical,emotional and sexual violence in districts of India, 2015-16.

State name	District name	Physical violence		Emotional violence		Sexual violence	
		Direct-survey based estimates	Model-based estimates	Direct-survey based estimates	Model-based estimates	Direct-survey based estimates	Model-based estimates
		Estimates (Lower bound, Upper bound)	Estimates (Lower bound, Upper bound)	Estimates (Lower bound, Upper bound)	Estimates (Lower bound, Upper bound)	Estimates (Lower bound, Upper bound)	Estimates (Lower bound, Upper bound)
Kerala	Kozhikode	9.9(3.8,23.4)	7.0(6.7,7.2)	8.7(3.3,21.2)	5.5(5.2,5.7)	5.5(2.0,14.0)	3.7(3.5,3.8)
Kerala	Malappuram	6.3(2.0,18.2)	7.0(6.8,7.2)	2.2(0.5,9.2)	5.5(5.3,5.7)	2.8(0.8,9.7)	2.2(2.1,2.4)
Kerala	Palakkad	7.3(3.9,13.0)	9.0(8.7,9.3)	3.4(1.2,9.2)	6.8(6.6,7.1)	1.5(0.5,4.4)	3.7(3.5,3.9)
Kerala	Thrissur	8.8(3.8,18.8)	5.9(5.7,6.1)	6.7(3.0,14.3)	5.3(5.0,5.5)	3.9(1.1,12.8)	2.6(2.5,2.8)
Kerala	Ernakulam	3.5(1.4,8.2)	8.2(8.0,8.5)	6.5(3.4,12.3)	7.9(7.6,8.1)	0.7(0.1,5.2)	1.5(1.4,1.6)
Kerala	Idukki	8.0(3.9,15.8)	13.5(13.0,14.1)	5.2(2.2,11.9)	9.8(9.3,10.3)	1.1(0.1,8.0)	3.3(3.0,3.6)
Kerala	Kottayam	10.4(6.0,17.6)	8.3(7.9,8.7)	5.8(2.1,14.8)	8.2(7.8,8.5)	2.5(0.6,10.6)	4.0(3.7,4.2)
Kerala	Alappuzha	8.2(4.2,15.2)	9.3(8.9,9.6)	8.7(4.7,15.7)	5.5(5.3,5.8)	4.2(1.5,11.0)	2.4(2.3,2.6)
Kerala	Pathanamthitta	12.0(5.4,24.4)	8.1(7.7,8.5)	16.9(10.0,27.1)	8.4(7.9,8.8)	8.8(3.0,22.9)	3.9(3.6,4.2)
Kerala	Kollam	11.6(7.6,17.3)	4.5(4.3,4.8)	8.0(4.1,14.9)	5.8(5.5,6.0)	2.9(0.8,9.7)	1.9(1.8,2.1)
Kerala	Thiruvananthapuram	9.3(5.2,16.2)	8.0(7.7,8.3)	8.9(5.5,14.3)	6.8(6.5,7.0)	2.5(0.4,14.8)	4.0(3.8,4.1)
Tamil Nadu	Thiruvallur	30.1(20.3,42.2)	33.2(32.8,33.6)	10.6(4.4,23.5)	19.0(18.6,19.3)	4.6(1.4,13.8)	6.5(6.3,6.7)
Tamil Nadu	Chennai	17.8(13.4,23.2)	20.8(20.5,21.1)	21.5(14.0,31.6)	20.1(19.8,20.4)	13.9(8.1,22.9)	10.3(10.1,10.5)
Tamil Nadu	Kancheepuram	33.8(21.8,48.3)	30.5(30.1,30.8)	25.9(14.5,42.0)	17.7(17.4,18.0)	4.6(1.9,10.4)	4.0(3.9,4.2)
Tamil Nadu	Vellore	30.1(17.6,46.6)	38.9(38.5,39.3)	7.2(4.0,12.5)	21.3(20.9,21.6)	3.0(1.1,8.1)	7.3(7.1,7.5)
Tamil Nadu	Tiruvannamalai	46.6(36.4,57.1)	43.5(43.0,44.1)	22.8(13.8,35.2)	25.7(25.3,26.2)	5.3(2.5,10.7)	7.3(7.0,7.6)
Tamil Nadu	Viluppuram	49.6(43.2,56.0)	47.8(47.3,48.2)	32.2(20.3,47.1)	27.7(27.3,28.1)	11.9(5.7,23.2)	10.7(10.4,11.0)
Tamil Nadu	Salem	40.9(24.6,59.4)	31.4(30.9,31.8)	34.9(19.8,53.8)	18.7(18.3,19.0)	16.4(7.5,32.3)	7.3(7.0,7.5)
Tamil Nadu	Namakkal	31.4(21.4,43.5)	33.0(32.4,33.6)	18.1(12.1,26.1)	19.6(19.1,20.1)	3.5(1.0,11.4)	6.1(5.8,6.4)
Tamil Nadu	Erode	26.5(18.8,36.0)	31.3(30.8,31.8)	22.2(15.1,31.4)	17.9(17.4,18.3)	5.1(2.2,11.1)	4.8(4.5,5.0)
Tamil Nadu	The Nilgiris	46.9(35.1,59.0)	35.4(34.5,36.4)	28.4(18.5,41.0)	23.9(23.1,24.8)	11.4(4.0,28.4)	7.9(7.4,8.4)
Tamil Nadu	Dindigul	23.8(14.3,37.0)	30.8(30.3,31.3)	7.5(3.4,15.7)	15.9(15.5,16.3)	2.1(0.6,7.6)	5.3(5.0,5.5)
Tamil Nadu	Karur	34.9(23.9,47.9)	33.8(33.0,34.6)	24.9(16.6,35.6)	21.8(21.1,22.5)	4.4(1.8,10.4)	6.1(5.7,6.5)
Tamil Nadu	Tiruchirappalli	40.9(27.9,55.2)	32.9(32.4,33.4)	9.5(5.4,16.3)	21.5(21.0,21.9)	3.5(1.3,8.7)	6.7(6.5,7.0)
Tamil Nadu	Perambalur	40.4(29.4,52.5)	39.2(38.1,40.3)	29.8(21.8,39.3)	24.2(23.2,25.2)	5.7(1.9,15.7)	7.9(7.3,8.5)
Tamil Nadu	Ariyalur	42.4(30.5,55.1)	37.4(36.4,38.4)	31.6(21.9,43.2)	25.1(24.2,25.9)	10.7(4.3,24.2)	9.4(8.8,10.0)
Tamil Nadu	Cuddalore	41.6(27.0,57.9)	45.1(44.6,45.6)	20.1(10.2,35.7)	25.1(24.6,25.6)	11.8(4.7,26.9)	8.6(8.3,8.9)
Tamil Nadu	Nagapattinam	33.1(24.8,42.5)	32.7(32.1,33.4)	21.4(14.6,30.4)	19.0(18.4,19.5)	6.0(2.7,12.9)	5.4(5.1,5.7)
Tamil Nadu	Thiruvarur	37.2(23.2,53.7)	36.0(35.2,36.7)	19.0(8.6,37.0)	17.3(16.7,17.9)	8.4(3.6,18.1)	6.2(5.8,6.6)
Tamil Nadu	Thanjavur	43.3(29.9,57.7)	36.0(35.5,36.6)	16.3(8.9,27.9)	20.7(20.3,21.2)	4.5(1.8,10.5)	6.1(5.8,6.3)
Tamil Nadu	Pudukkottai	34.8(24.2,47.1)	35.8(35.1,36.4)	29.8(19.7,42.2)	27.4(26.8,28.0)	7.9(4.3,14.3)	6.5(6.2,6.8)
Tamil Nadu	Sivaganga	39.6(23.8,57.8)	40.1(39.4,40.8)	29.2(23.3,35.9)	30.2(29.5,30.8)	10.0(4.6,20.4)	11.0(10.5,11.4)
Tamil Nadu	Madurai	26.7(18.4,37.1)	37.8(37.3,38.2)	17.8(13.2,23.7)	16.5(16.1,16.8)	9.9(5.8,16.4)	6.9(6.6,7.1)
Tamil Nadu	Theni	35.6(22.5,51.3)	36.2(35.4,36.9)	21.0(10.9,36.5)	19.4(18.8,20.0)	6.8(2.8,15.8)	6.9(6.6,7.3)
Tamil Nadu	Virudhunagar	19.3(6.3,45.9)	23.4(22.9,23.9)	25.8(9.0,55.1)	17.7(17.2,18.1)	4.9(1.4,16.0)	3.8(3.5,4.0)
Tamil Nadu	Ramanathapuram	41.1(33.1,49.7)	37.1(36.4,37.8)	27.5(17.8,39.9)	20.7(20.2,21.3)	6.7(2.1,19.3)	5.8(5.5,6.2)
Tamil Nadu	Thoothukkudi	24.6(17.3,33.6)	38.5(37.8,39.1)	9.4(5.7,15.2)	15.7(15.2,16.2)	4.9(2.5,9.4)	4.5(4.2,4.7)
Tamil Nadu	Tirunelveli	30.5(21.1,42.0)	27.3(26.9,27.8)	11.6(5.2,23.6)	17.1(16.7,17.5)	4.7(2.5,8.5)	7.1(6.9,7.4)
Tamil Nadu	Kanniyakumari	24.7(17.3,34.0)	23.0(22.5,23.6)	15.4(8.8,25.6)	13.7(13.2,14.1)	6.4(2.7,14.2)	6.3(6.0,6.6)
Tamil Nadu	Dharmapuri	50.6(39.4,61.8)	36.0(35.3,36.6)	23.6(15.5,34.2)	20.4(19.9,21.0)	2.4(0.8,7.0)	4.9(4.6,5.2)
Tamil Nadu	Krishnagiri	28.5(19.7,39.2)	35.0(34.4,35.6)	14.6(8.7,23.5)	17.7(17.2,18.2)	4.4(1.9,9.8)	6.0(5.7,6.3)
Tamil Nadu	Coimbatore	28.8(21.4,37.6)	30.0(29.6,30.4)	14.7(9.3,22.4)	14.7(14.4,15.0)	5.7(2.8,11.1)	5.7(5.5,5.9)
Tamil Nadu	Tiruppur	22.4(15.7,30.8)	28.7(28.3,29.2)	5.2(1.8,14.3)	16.5(16.1,16.9)	0.6(0.1,3.7)	3.1(2.9,3.2)
Puducherry	Yanam	33.3(19.1,51.4)	33.0(29.6,36.4)	15.9(7.9,29.6)	16.9(14.2,19.6)	1.1(0.3,4.3)	3.4(2.0,4.7)
Puducherry	Puducherry	23.8(12.9,39.8)	25.1(24.4,25.9)	23.0(12.4,38.6)	19.3(18.6,20.0)	11.3(5.5,21.8)	5.5(5.1,5.9)
Puducherry	Mahe	12.8(6.9,22.4)	16.2(13.2,19.2)	6.9(3.1,14.8)	10.1(7.7,12.5)	0.0(0.0,0.0)	2.6(1.3,3.8)
Puducherry	Karaikal	33.6(20.3,50.1)	29.2(27.5,31.0)	20.0(10.6,34.5)	19.7(18.1,21.2)	4.7(2.1,10.1)	5.7(4.8,6.6)
Andaman & Nicobar Islands	Nicobars	7.0(1.1,32.7)	10.2(7.2,13.1)	7.3(3.2,15.7)	7.5(4.9,10.1)	1.5(0.2,10.1)	1.9(0.5,3.2)
Andaman & Nicobar Islands	North & Middle Andaman	11.5(6.4,19.8)	10.8(9.1,12.4)	2.2(0.6,8.1)	6.4(5.1,7.7)	2.0(0.5,7.9)	1.7(1.0,2.4)
Andaman & Nicobar Islands	South Andaman	11.8(5.7,22.8)	9.4(8.4,10.4)	10.9(3.8,27.5)	6.7(5.8,7.5)	0.9(0.1,6.2)	1.2(0.8,1.6)

Table A3: Within state variation in estimate of IPV using direct survey-based and model-based estimates in India, 2015-16							
State	No of districts	Physical Violence		Emotional Violence		Sexual Violence	
		Direct survey-based CV	Model-based CV	Direct survey-based CV	Model-based CV	Direct survey-based CV	Model-based CV
Andaman & Nicobar Islands	3	0.267	0.067	0.642	0.082	0.379	0.215
Andhra Pradesh	13	0.285	0.116	0.458	0.213	0.587	0.300
Arunachal Pradesh	16	0.441	0.191	0.498	0.207	0.849	0.454
Assam	27	0.365	0.225	0.664	0.274	0.543	0.412
Bihar	38	0.201	0.129	0.270	0.134	0.480	0.268
Chandigarh	1	0.000	0.000	0.000	0.000	0.000	0.000
Chhattisgarh	18	0.216	0.158	0.516	0.228	0.599	0.260
Dadra and Nagar Haveli	1	0.000	0.000	0.000	0.000	0.000	0.000
Daman and Diu	1	1.118	0.277	0.710	0.249	1.414	0.697
Goa	2	0.490	0.078	0.202	0.051	1.414	0.403
Gujarat	26	0.511	0.231	0.516	0.257	1.067	0.389
Haryana	21	0.287	0.139	0.393	0.161	0.626	0.338
Himanchal Pradesh	12	0.601	0.223	0.634	0.296	0.786	0.303
Jammu & Kashmir	22	0.505	0.200	0.645	0.308	1.091	0.418
Jharkhand	24	0.405	0.170	0.592	0.205	0.775	0.303
Karnataka	30	0.383	0.193	0.536	0.170	0.737	0.342
Kerala	14	0.306	0.274	0.593	0.223	0.811	0.310
Lakshadweep	1	0.000	0.000	0.000	0.000	0.000	0.000
Madhya Pradesh	50	0.336	0.173	0.455	0.169	0.692	0.308
Maharashtra	35	0.368	0.170	0.534	0.232	1.091	0.328
Manipur	9	0.226	0.195	0.338	0.145	0.502	0.234
Meghalaya	7	0.532	0.251	0.491	0.310	1.090	0.413
Mizoram	8	0.325	0.175	0.183	0.178	0.500	0.264
Nagaland	11	0.971	0.242	0.490	0.180	0.729	0.538
NCT of Delhi	9	0.315	0.213	0.536	0.139	0.843	0.223
Odisha	30	0.278	0.183	0.356	0.203	0.763	0.286
Puducherry	4	0.381	0.279	0.424	0.269	1.190	0.367
Punjab	20	0.424	0.225	0.508	0.156	0.834	0.451
Rajasthan	33	0.410	0.121	0.652	0.247	0.791	0.313
Sikkim	4	0.345	0.242	0.763	0.079	0.670	0.324
Tamil Nadu	32	0.256	0.174	0.401	0.200	0.553	0.285
Telangana	10	0.261	0.096	0.440	0.204	0.859	0.345
Tripura	4	0.193	0.211	0.113	0.083	0.151	0.106
Uttar Pradesh	71	0.356	0.143	0.505	0.205	0.733	0.318
Uttarakhand	13	0.433	0.184	1.030	0.265	0.723	0.399
West Bengal	19	0.412	0.241	0.540	0.358	0.777	0.425

Figure A1 Geospatial estimation of Border Proximity Factor (BPF).

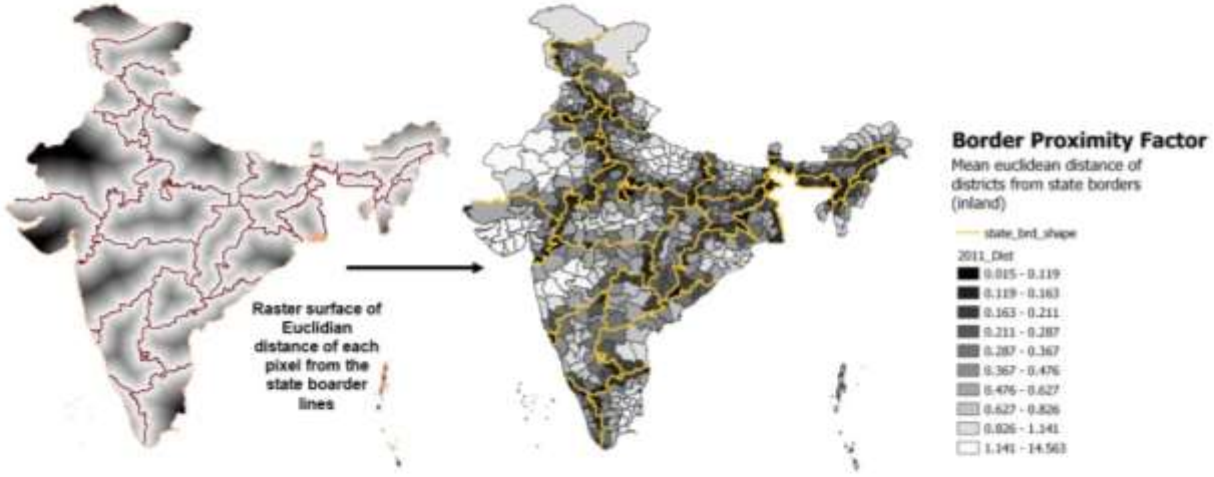

Supplement: Supplementary appendix [file mmc1.pdf]
